# The structural basis of Cdc7-Dbf4 kinase dependent targeting and phosphorylation of the MCM2-7 double hexamer

**DOI:** 10.1038/s41467-022-30576-1

**Published:** 2022-05-25

**Authors:** Almutasem Saleh, Yasunori Noguchi, Ricardo Aramayo, Marina E. Ivanova, Kathryn M. Stevens, Alex Montoya, S. Sunidhi, Nicolas Lopez Carranza, Marcin J. Skwark, Christian Speck

**Affiliations:** 1 DNA Replication Group, Institute of Clinical Sciences, Faculty of Medicine, Imperial College London, Du Cane Road, London, W12 0NN UK; 2 MRC London Institute of Medical Sciences (LMS), Du Cane Road, London, W12 0NN UK; 3 Proteomics and Metabolomics Facility, MRC London Institute of Medical Sciences, Imperial College London, Hammersmith Hospital Campus, Du Cane Road, London, W12 0NN UK; 4 InstaDeep Ltd, 5 Merchant Square, London, W2 1AY UK

**Keywords:** Cryoelectron microscopy, Kinases

## Abstract

The controlled assembly of replication forks is critical for genome stability. The Dbf4-dependent Cdc7 kinase (DDK) initiates replisome assembly by phosphorylating the MCM2-7 replicative helicase at the N-terminal tails of Mcm2, Mcm4 and Mcm6. At present, it remains poorly understood how DDK docks onto the helicase and how the kinase targets distal Mcm subunits for phosphorylation. Using cryo-electron microscopy and biochemical analysis we discovered that an interaction between the HBRCT domain of Dbf4 with Mcm2 serves as an anchoring point, which supports binding of DDK across the MCM2-7 double-hexamer interface and phosphorylation of Mcm4 on the opposite hexamer. Moreover, a rotation of DDK along its anchoring point allows phosphorylation of Mcm2 and Mcm6. In summary, our work provides fundamental insights into DDK structure, control and selective activation of the MCM2-7 helicase during DNA replication. Importantly, these insights can be exploited for development of novel DDK inhibitors.

## Abstract (web summary)

Here the authors describe multiple structures of the replicative helicase MCM2-7 in complex with Dbf4-dependent kinase (DDK). These structures reveal why the kinase specifically recognizes the MCM2-7 double-hexamer over the single-hexamer and explain how DDK can phosphorylate distantly located Mcm tails.

## Introduction

The Cdc7 serine/threonine kinase has a wide range of cellular targets and it is involved in the regulation of several nuclear processes such as: DNA replication, DNA repair, chromatin organization, gene expression, chromosome cohesion and centriole/centrosome function^1–4^. Notably, Cdc7 is considered one of the most crucial regulators of the cell cycle, and it is one of only two kinases that are essential for the initiation of DNA replication^5–10^. The key target of Cdc7 is the MCM2-7 helicase, specifically the unstructured N-terminal tails of Mcm2, Mcm4 and Mcm6 which are known to be phosphorylated at multiple sites^11,12^. Mcm4 and Mcm2 have been reported to contain docking sites for DDK: the N-terminal A-domain of Mcm4 (aa175–333)^13^, the unstructured N-terminal tail of Mcm2 (aa2–63)^14^/(aa2–127)^15^, and the N-terminal A-domain of Mcm2 (aa204–278)^16^. Cdc7 is highly conserved in eukaryotes: indeed, Cdc7 is essential in yeast and its homozygous deletion causes embryonic lethality in mice^17–19^. Cdc7 on its own is not functional but requires a partner, dumbbell former 4 (Dbf4), for its kinase activity. When Dbf4 is bound to Cdc7 the resulting complex is referred to as Dbf4-dependent Cdc7 kinase (DDK). The complex is regulated through Dbf4, as Dbf4 protein stability is strictly cell cycle controlled, with low abundance in G1 phase, reaching maximum abundance in S-phase and staying high throughout mitosis^20,21^.

MCM2-7 is the catalytic core of the replicative helicase. In the G1 phase of the cell cycle, the helicase complex is loaded by ORC, Cdc6 and Cdt1 onto the origin DNA. In this multi-step reaction, two MCM2-7 hexamers are joined together to form a head-to-head double-hexamer, where both hexamers become connected via their N-terminal interfaces and encircle double-stranded DNA (dsDNA)^22,23^. This helicase complex is initially inactive until DDK dependent phosphorylation of the MCM2-7 double hexamer (DH) during the G1-S transition overcomes the autoinhibitory activity of the Mcm4 N-terminal tail^13,24,25^. This, in turn, then allows the next helicase activation complex, Sld3-Sld7, to interact with the phosphorylated N-terminal tails of Mcm4 and Mcm6^26^. Subsequently, Sld3-Sld7 promotes the binding of Cdc45, while Sld2, Dpb11, GINS and Polymerase ε (Polε) become recruited upon S-phase specific cyclin-dependent kinase (CDK) activation^27,28^. These steps yield two Cdc45-MCM2-7-GINS (CMG) helicase complexes, which represent the core of the replication fork^29–31^.

Eukaryotic genomes have hundreds to thousands of replication origins. It is imperative to limit their activation, as it would otherwise overwhelm the cells’ ability to produce sufficient deoxy-nucleotides and deplete the limited levels of available replication protein A (RPA), resulting in replication fork stalling or leaving stretches of single-stranded DNA (ssDNA) unprotected from DNA damage, respectively^32,33^. Two mechanisms contribute towards the control of DNA synthesis: (1) it has been shown that Dbf4 has a very low abundance in comparison to many other replication factors and that Dbf4 protein levels dictate the rate of DNA replication during S-phase by restricting the number of replication origins that can fire at any given time, and in this way the cell is protected from the stresses of RPA and nucleotide depletion^34,35^. (2) moreover, interaction studies have suggested that DDK docks onto the MCM2-7 substrate^13,14^. Thus, substoichiometric abundance of Dbf4 over MCM2-7^34^ and a stable MCM2-7-DDK interaction would mean that the levels of free DDK are limited, consequently preventing DDK from reaching many of its other targets and in this way restraining DNA replication in the early S-phase^36^.

Both DDK subunits, Cdc7 and Dbf4, are frequently found to be overexpressed in cancer and this is correlated with cancer development and poor prognosis^37–39^. Inhibition of DDK activity causes apoptosis in cancer cells, but not in normal cells, and therefore Cdc7 is seen as an attractive therapeutic target. Indeed, crystal structures of the human Cdc7 core in complex with small conserved regions of Dbf4^40,41^ have led to a number of drug discovery projects and currently several inhibitors are in various stages of clinical development^6,42,43^.

Despite extensive research into DDK, many molecular mechanisms are still poorly understood. How DDK can dock on MCM2-7 and then reach its distant target sites in Mcm2, Mcm4 and Mcm6 still remain unknown. It is also unclear when and how DDK disengages from MCM2-7. Here, we have used cryogenic electron microscopy (cryo-EM) and molecular dynamics simulations to investigate the DDK-MCM2-7 DH complex^13,14,16^ in order to understand how DDK recruitment, specificity for the MCM2-7 DH^25^ and phosphorylation of MCM2-7 works at a structural level.

## Results

### Reconstitution and structural analysis of the MCM2-7-DDK complex

We have developed an approach to reconstitute the full-length *Saccharomyces cerevisiae* MCM2-7 double-hexamer - DDK (MD) complex (Supplementary Fig. 1a–e). Using origin DNA coupled to magnetic beads we assembled the MCM2-7 DH helicase complex, released the complex from the beads, added DDK and performed glycerol gradient sedimentation and simultaneous mild fixation by glutaraldehyde (GraFix) (Supplementary Fig. 1a). The glycerol gradient peak fractions showed the presence of a near stoichiometric MD complex, which indicated that the complex is stable (Supplementary Fig. 1c). In order to limit ATP-hydrolysis dependent structural changes, we used ATPγS, a slowly hydrolysable analogue form of ATP. To understand whether DDK can use ATPγS to thio-phosphorylate proteins, we performed proteomic analysis of the sample. This revealed thio-phosphorylation of previously characterised serine and threonine residues in Dbf4, Mcm2, Mcm4, Mcm6 and Mcm7 (Supplementary Fig. 2 and Supplementary Table 1), indicating that ATPγS does not affect the overall DDK specificity^12^. Moreover, we found that DDK phosphorylation of MCM2-7 is inhibited by the human Cdc7 inhibitors: XL413, PHA767491 and TAK931 (Supplementary Fig. 1f), suggesting that the ATP binding pockets of yeast and human Cdc7 are conserved.

**Fig. 1:**
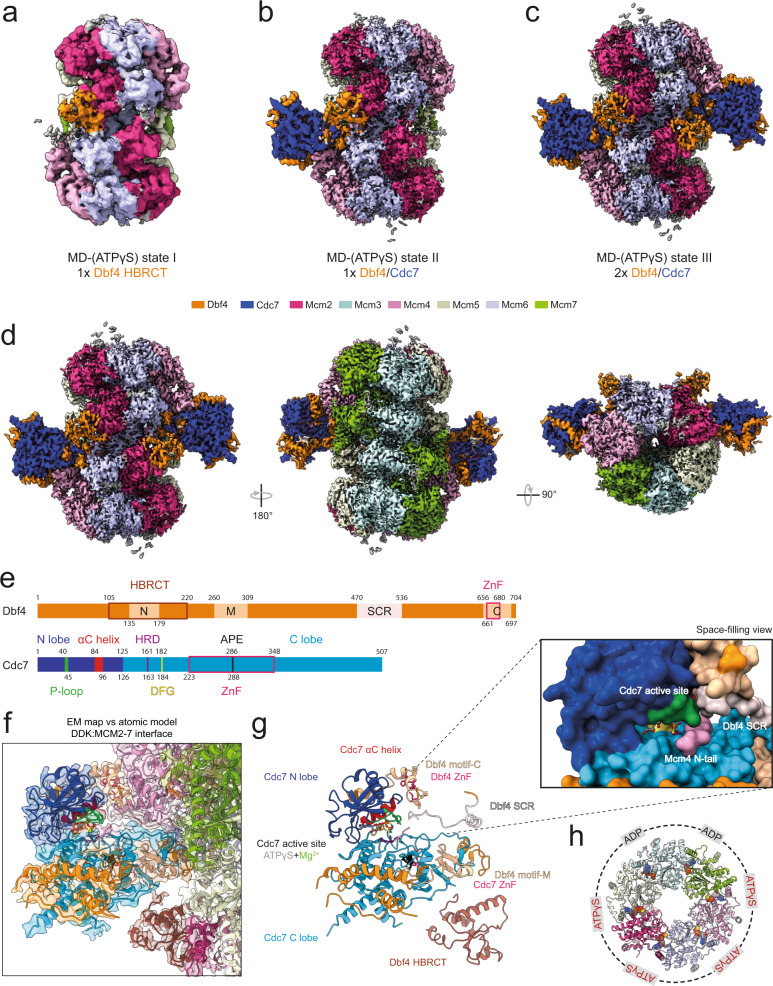
Structure of DDK bound to the MCM2-7 double hexamer in the presence of ATPγS. **a**–**d** Three different structural states (I-III) derived from the same MD-(ATPγS) cryo-EM data set. **a** Cryo-EM 3D auto-refined map (see Methods) of MD-(ATPγS) state I. **b** Composite map (see Methods) of MD-(ATPγS) state II. **c**, **d** Composite map (see Methods) of MD-(ATPγS) state III with side and top views. DH at 3.2 Å mean resolution and DDK at 3.6 Å mean resolution. The map density corresponding to each protein subunit component of the complex is coloured according to the key shown. **e** A schematic diagram illustrating the 2D domain organization the 2D domain organization of Dbf4 and Cdc7. **f** Comparison of the MD-(ATPγS) atomic model to the cryo-EM map to show the quality of fit. EM map and atomic model are coloured according to key shown in (**e**). **g** Same view as (**f**), but focused only on DDK. The structural features of Cdc7 and Dbf4 are indicated, and a close-up view of the active site is shown. **h** Overview of the nucleotide occupancy and type in each Mcm subunit within the MD-(ATPγS) complex.

Next, we employed single-particle cryo-EM analysis to study the structure of the full-length MD-(ATPγS) complex. 3D classification of the MD-(ATPγS) data revealed three different structural states (state I-III) of the complex (Fig. 1a–c, Supplementary Movie 1). In state I, we observed predominantly an individual Dbf4 Helix-BRCA1 C-terminal (HBRCT) domain, which also harbours Dbf4 motif-N, bound to the Mcm2 N-terminal domain of the DH, while the remaining sections of DDK were not well resolved. This state suggests that the Dbf4 HBRCT domain makes initial contact with the DH or represents DDK release intermediate. In state II, we observe only one DDK being fully recruited to the MCM2-7 DH in an Mcm4-targeted DDK conformation, while the second DDK binding site is left unoccupied. In state III, two DDK molecules are bound to the MCM2-7 DH interface, again in an Mcm4 targeted fashion. However, the binding of the second DDK molecule was not associated with structural change in MCM2-7 or DDK. The three structural states together show that DDK recruitment is a dynamic process. In particular: we observed that the Dbf4 HBRCT domain is sufficient for stable MCM2-7 interaction, identified a hinge between the Dbf4 HBRCT and the core of the DDK kinase and finally the data demonstrate that two DDK molecules can bind simultaneously to the DH. In these three structural states, the binding of one DDK does not appear to induce any structural changes at the second DDK binding site, and due to this we propose that the two DDK molecules can bind independently to the MCM2-7 DH.

Out of the three MD complexes, state III was resolved to the highest mean resolution of 3.1 Å, with DDK having a local resolution of 3.5 Å (Fig. 1d and Supplementary Fig. 3). We resolved density for 87% of Cdc7, including all conserved kinase motifs, 41% of Dbf4, including the conserved N, M and C motifs and 72% of MCM2-7 (Fig. 1c–e and Supplementary Fig. 4). In the following sections, we will use state III to describe the overall structure of the MD complex.

### The Dbf4 HBRCT domain is required to make initial contact with MCM2-7

**Fig. 2:**
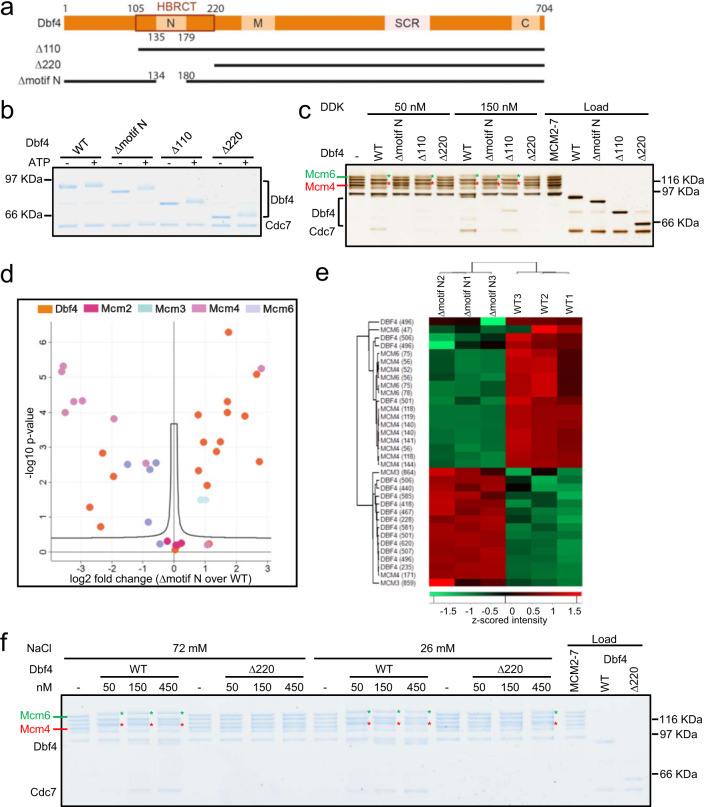
The Dbf4 N-terminus is important for DDK dependent phosphorylation of MCM2-7. **a** Schematic of Dbf4 N-terminal mutants. The Dbf4 regulatory protein features three unique motifs and a modified domain: motif-N, motif-M and motif-C and α-helix-BRCA1 C-terminal (HBRCT) domain. The substrate coordinating region (SCR) is a newly identified domain. **b** Analysis and comparison of Dbf4-Cdc7 autophosphorylation ability using Dbf4 wild-type and N-terminal mutants. Similar results were obtained in two independent experiments. **c** Analysis of Dbf4 N-terminal mutants assessing the interaction of DDK with MCM2-7 DH and MCM2-7 phosphorylation (using 50 and 150 nM DDK concentration). Similar results were obtained in three independent experiments. **d** Volcano plot comparing the DDK phosphorylation profile of the MCM2-7 DH of WT and Δmotif N Dbf4. Two-sample Student’s *t*-test carried out with three replicate intensities considered per group. *P*-values were corrected for multiple comparisons to an FDR of 0.05 (permutation-based FDR). **e** Volcano plot significant phosphosites visualised using Hierarchical Clustering Analysis (HCA) coupled to a heatmap of z-scored site intensities. **f** Analysis of WT and Δ220 Dbf4 using different protein and salt concentrations. The results highlight that DDK regions within Δ220 Dbf4 do not support binding to the MCM2-7 DH and only very weakly support MCM2-7 phosphorylation activity. Similar results were obtained in two independent experiments.

To address whether the Dbf4 N-terminus has a role in DDK recruitment to the MCM2-7 DH or its release, we generated three mutants: Dbf4 Δ110 [missing the unstructured N-terminus - Δ2-110], Dbf4 Δmotif N [missing motif-N HBRCT domain – aa134–190] and Dbf4 Δ220 [missing the first 220 aa of Dbf4 – aa2–220] (Fig. 2a). All mutants were competent for Dbf4 autophosphorylation, but Dbf4 Δ220 abrogated recruitment to the MCM2-7 DH and its phosphorylation, while Dbf4 Δmotif N was defective for recruitment and MCM2-7 phosphorylation at 50 nM DDK and less so at the elevated protein concentration of 150 nM DDK (Fig. 2b, c). Moreover, the removal of motif-N resulted in an increase in autophosphorylation and a reduction in phosphorylation of Mcm4 and Mcm6 (Fig. 2d, e and Supplementary Table 1). On the other hand, Dbf4 Δ110 showed near WT DDK activities, especially at 150 nM DDK concentration. Interestingly, increasing the concentration of Dbf4 Δ220 or lowering the ionic strength allowed us to identify residual Dbf4 Δ220 dependent phosphorylation of MCM2-7 (Fig. 2f), which is consistent with the observation that this domain is not essential for viability^44^. In summary, the biochemical and structural data strongly argue that the Dbf4 110–220, encompassing the HBRCT domain, functions for DDK recruitment to the MCM2-7 DH and its phosphorylation.

### The MCM2-7 DH conformation in context of ATPγS and DDK binding

The MCM2-7 subunits in the MD-(ATPγS) structure were found to adopt the same arrangement and conformation compared to that of the previously reported DH structure (PDB 5BK4)^45^, with the exception of a short additional resolved region of Mcm2 at the DDK-MCM2-7 interface. Thus, we conclude that the presence of DDK does not cause any large conformational changes to the rigid core of MCM2-7. We observed ATPγS in four Mcm subunit interfaces, with ADP present at the Mcm5/3 and Mcm3/7 interfaces (Fig. 1f). This contrasts the result seen with the DNA bound DH structure, which had no nucleotide at the Mcm7/4 and Mcm4/6 interfaces and ADP at the remaining nucleotide-binding sites. Thus, our data suggest that ATPγS did bind to the two sites that are empty in the DNA-bound DH structure and lead to ADP-to-ATPγS exchange at the two neighbouring Mcm6/2 and Mcm2/5 interfaces, which are occupied by ADP in the DNA-bound DH^45^. This contrasts previous biochemical observations^46^, which did not detect significant ADP release, potentially due to the longer incubation time or the addition of DDK in our experiments.

### The Cdc7 active site binds a short peptide

In the MD-(ATPγS) structure (Fig. 1e–g), Cdc7 features a bilobal shape that is common amongst eukaryotic protein kinases^47^, with an active site located in a deep cleft between its N- and C-terminal lobe. Within the active site, we detected ATPγS, a Mg^2+^ ion and a short peptide fragment positioned within the substrate-binding region (Fig. 1g). The fragment is located closest to the most N-terminal resolved region of Mcm4 and the Cdc7 active site is directed towards Mcm4, away from Mcm2 and Mcm6, suggesting that the peptide belongs to the flexible N-terminal tail of Mcm4. The peptide amino acid sequence was not resolved, but contains a bulky side chain at the P + 1 site, consistent with the DDK preference of an acidic or phosphorylated residue in that position^48^.

### Cdc7 adopts an active conformation and makes no contact with the core of the DH

**Fig. 3:**
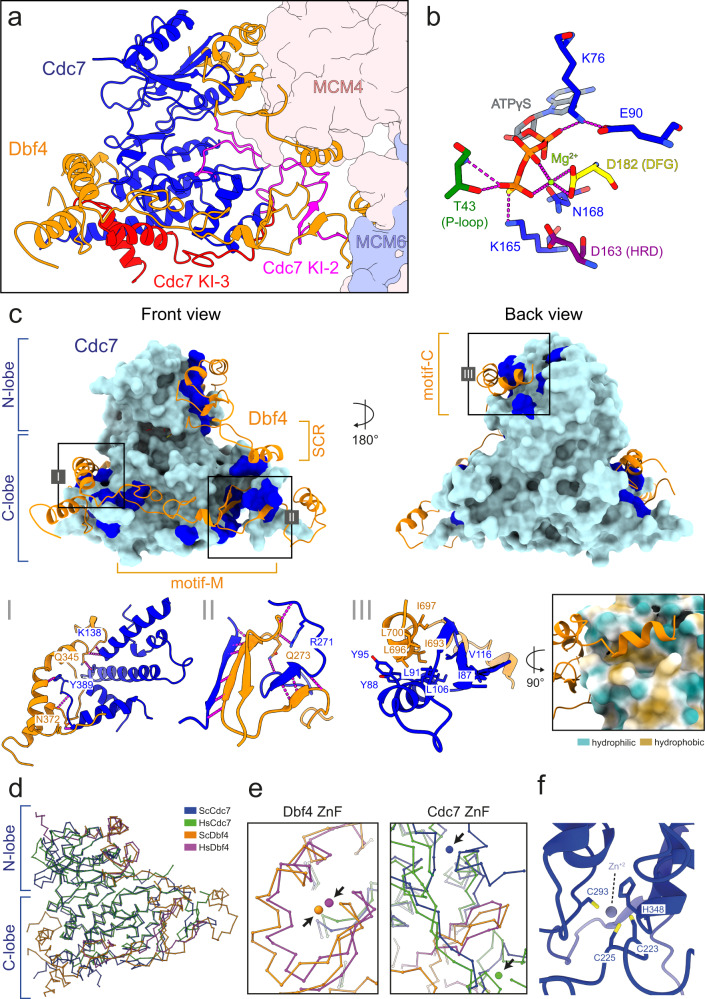
DDK organization and intra-molecular interactions. **a** Front view of DDK bound to the DH. The Cdc7 kinase insert (KI) regions are indicated, KI-2 in magenta and KI-3 in red. **b** Zoomed view of the Cdc7 active site bound to ATPγS and a Mg^2+^ ion. The Cdc7 residues surrounding the nucleotide are shown. D182 (DFG motif), T43 (P-loop), and D163 (HRD motif) are coloured in yellow, dark green and purple, respectively and other Cdc7 residues are coloured in blue. **c** Front and back view of the core of DDK. The surface of Cdc7 is coloured in cyan and regions which contact Dbf4 are highlighted in blue. Zoomed view of the interaction interfaces between: I Dbf4 motif-M and a helical bundle within the Cdc7 C-lobe, II Dbf4 motif-M and Cdc7 KI-3 and III Dbf4 motif-C and a hydrophobic groove on the surface of the Cdc7 N-lobe. **a**–**c** Cdc7 and Dbf4 are coloured in blue and orange, respectively and hydrogen bond interactions are represented by dotted magenta-coloured lines. **d** Overall 3D organization of the core of human DDK (PDB:6YA7) and budding yeast DDK (MD-(ATPγS)). Both kinase structures are highly similar and display an active kinase conformation. **e** Overlay of the zinc finger (ZnF) domains of Dbf4 motif-C and Cdc7 from human and budding yeast. The Dbf4 ZnF is at same 3D position and the Cdc7 ZnF is located at the back of Cdc7 but deviates by 21 Å. **f** Zoomed view of the budding yeast Cdc7 ZnF and the residues involved in zinc ion coordination.

The mainly α-helical C-lobe of Cdc7 contains multiple conserved amino acid sequence motifs (Figs. 1e–g and 3b): I.; a conserved HRD motif (aa161–163), consisting of a histidine (H) that anchors the region in close proximity to the kinase active site, and an arginine (R) that is adjacent to the key catalytic aspartate (D) that facilitates the phosphotransfer, II.; the DFG motif (aa182–184) is observed in its active conformation with the phenylalanine (F) being positioned away from the ATP binding pocket and the aspartate (D) interacting with a Mg^2+^ ion, and lastly III.; the APE motif (aa286–288), which helps to position the activation loop^47,49^.

The activation loop region is defined by the start of the DFG motif and the end of APE motif, with the kinase insert 2 located in-between the two motifs^41^. Here, we have resolved large sections of the activation loop, which makes direct contacts with Dbf4 and Mcm4 and may contribute to anchoring the activation loop in the active site (Fig. 3a and Supplementary Fig. 4).

At the active site itself, ATPγS is held from the top by K76 and E90 of the N-lobe, which together stabilise the α phosphate of ATP during catalysis (Fig. 3b). In addition, T43 of the P-loop/GEGTFS motif (aa40–45) and invariant K165 contacts the thio-phosphate. Below ATPγS, D182 positions the Mg^2+^ ion from one side and N168 from the other side. Notably, Cdc7 adopts an active configuration that is primed for phosphorylation and, with the exception of the substrate-binding site, makes little to no contacts with the core of MCM2-7.

### The Dbf4–Cdc7 interface and two zinc-binding sites restrain the Cdc7 conformation

Cdc7 and Dbf4 form a stable complex in the cell, with the overall DDK activity being controlled by Dbf4 protein levels^7^. The MD structure allowed us to resolve extended parts of Dbf4. There are three different contact points (I–III) between Dbf4 and Cdc7 (Fig. 3c; Supplementary Table 2): (I) the Dbf4 motif-M binds to kinase insert 3 of the Cdc7 C-lobe with the N-terminal part of the motif-M forming together a three-stranded beta-sheet (Fig. 3a, c). (II) a helix bundle composed of Dbf4 aa310–391 binds to another section of the KI-3 in the Cdc7 C-lobe (Fig. 3a, c). (III) finally, the Dbf4 motif-C binds Cdc7 at the back of the N-lobe, and in this way provides an additional connector between the C- and N-lobe of Cdc7 (Fig. 3a, c). In summary, Dbf4 is enfolding Cdc7 by gripping it from three sides and thereby restraining the Cdc7 structure. Comparing budding yeast DDK to the structure of human Cdc7 in complex with Dbf4 motifs -M and -C reveals highly similar 3D organisation (Fig. 3d). The zinc-binding site formed by yeast and human Dbf4 proteins is highly conserved and helps to keep the Cdc7 αC helix and kinase insert 2 in their “kinase active” state, as described in the context of human Cdc7^41^ (Fig. 3e, f). Even though residues forming a second zinc-binding site are not conserved between human and yeast Cdc7, a zinc ion is observed in a nearby alternative location relative to the structure of the human Cdc7, and may therefore stabilise a similar section of the kinase^41^.

### Multiple weak Dbf4–MCM interactions are arranged across the DH interface

Kinases mainly form transient, weak interactions with their substrates involving a small region along the cleft of the kinase active site^47^. Weak interactions allow for quick release of the substrate once phosphorylation has occurred. We have observed that DDK is an unusual kinase, as it forms a stable complex with its primary substrate, MCM2-7, as seen in glycerol gradient sedimentation experiments performed at 4 °C and bead-based origin assembly assays performed at 24 °C (Supplementary Fig. 1c, g). This stable interaction offers the opportunity to characterise the large DDK-substrate interaction surface and investigate how various structurally disordered Mcm N-termini are targeted for phosphorylation.

**Fig. 4:**
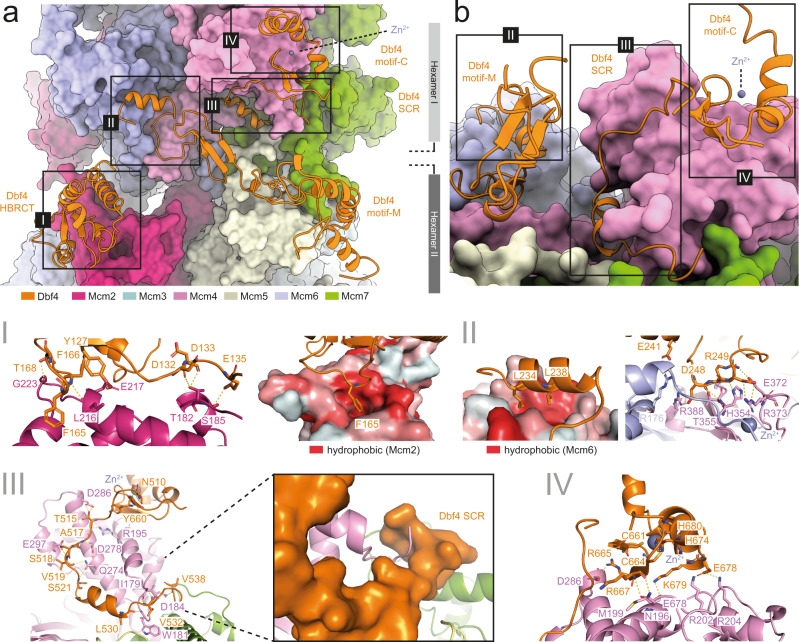
Analysis of the DDK:MCM2-7 interaction interface. **a** Cartoon view of Dbf4 and space-filling view of the MCM2-7 DH. Dbf4 forms four independent interaction interfaces along the surface of the MCM-2-7 DH. **b** Enlarged and rotated view of (**a**) showing interaction interfaces II–IV. I–IV Detailed view of each of the four Dbf4:Mcm interaction interfaces. The residues involved in making contacts are labelled. The hydrophobic surface shown is coloured according to the Eisenberg hydrophobicity scale.

We have found that Dbf4 is solely responsible for DDK substrate recognition and forms four independent interactions (SI-IV) with the substrate—MCM2-7 (Fig. 4; Supplementary Table 3).

Substrate interaction region I (SI) consists of the Dbf4 N-terminal section (aa111–220), which corresponds to the Dbf4 HBRCT domain (aa105–220) and interacts with the Mcm2 N-terminal domain (NTD) (aa179–285). Interaction I involves an invariant Dbf4 phenylalanine (F165) (Supplementary Fig. 5b) that binds into a deep hydrophobic pocket on the surface of Mcm2 of hexamer 1 (Fig. 4a, b; Supplementary Fig. 6). In addition, there are a series of polar interactions that hold the Dbf4 HBRCT domain at the Mcm2 surface (Fig. 4b–i; Supplementary Fig. 6).

Substrate interaction II (SII) involves Dbf4 motif-N and -M connector (aa231–309) (Fig. 4), that binds to an interface of Mcm4 and Mcm6 (Fig. 4b-II) and to the zinc finger (ZnF) domain of Mcm4 on hexamer 2 (Fig. 4b; Supplementary Fig. 6).

Substrate interaction III (SIII) involves a Dbf4 motif -M and -C connector (aa509–538), which is highly conserved between different species of budding yeast (Fig. 4b-III, Supplementary Fig. 5b). The start of this Dbf4 region is held in place by interactions between Dbf4 motif-C and Cdc7, which involve a series of polar and ionic contacts; while the most C-terminal end of this region is held by a hydrophobic patch made up by Mcm4 (Fig. 4b; Supplementary Fig. 6). Interestingly, a section of region III (aa509–538; labelled as SCR in Fig. 1e–g) is located in close proximity to the Cdc7 P-loop and generates a surface along the active site cleft of Cdc7 directly opposite the substrate-binding site. Based on structural features, in particular its proximity to the active site and the Mcm4 N-terminal tail, and the sequence alignment (Supplementary Fig. 5b), we tentatively named the region as the substrate coordinating region (SCR, aa470–536).

The Dbf4 sections N-terminal and C-terminal of the SCR were not resolved, but we propose that these regions may form contacts with the substrate and could trap the substrate by encircling it within the Cdc7 active site.

Moreover, the Dbf4 SCR forms a lasso around the most N-terminal resolved region of Mcm4 and in this way may contribute towards the specificity of Cdc7 to Mcm4 (Fig. 4b-III). A large number of backbone-to-backbone contacts, in the lasso structure latching on Mcm4, could accommodate similar binding modes when binding and phosphorylating alternative DDK substrates.

Substrate interaction IV (SIV) involves the highly conserved motif-C of Dbf4 (aa656–697), which contains a Zn binding region and latches onto the surface of the Mcm4 NTD of hexamer II via a series of polar and backbone interactions (Fig. 4b; Supplementary Fig. 6).

Together these Dbf4-Mcm interactions position Cdc7 on the MCM2-7 double-hexamer in a unique way (Fig. 1d), which shields the kinase domain from N-terminal extensions of other Mcm proteins apart from the Mcm4-N-terminal tail. Thus, the observed kinase-substrate interaction is enhancing substrate selection for Mcm4.

Importantly, since Dbf4 initial docks to hexamer I via Mcm2, but then contacts Mcm4, Mcm6 and Mcm7 of hexamer 2 and also phosphorylates Mcm4 of hexamer II, the structure explains how DDK can specifically target the MCM2-7 double-hexamer over the single-hexamer (Fig. 4a). However, although Dbf4 contacts several Mcm subunits, each interaction surface is limited—frequently involving backbone interactions interspersed with few ionic or hydrophobic contacts that are only partially conserved between species (Supplementary Figs. 5b and 6).

### The SCR region provides substrate specificity

**Fig. 5:**
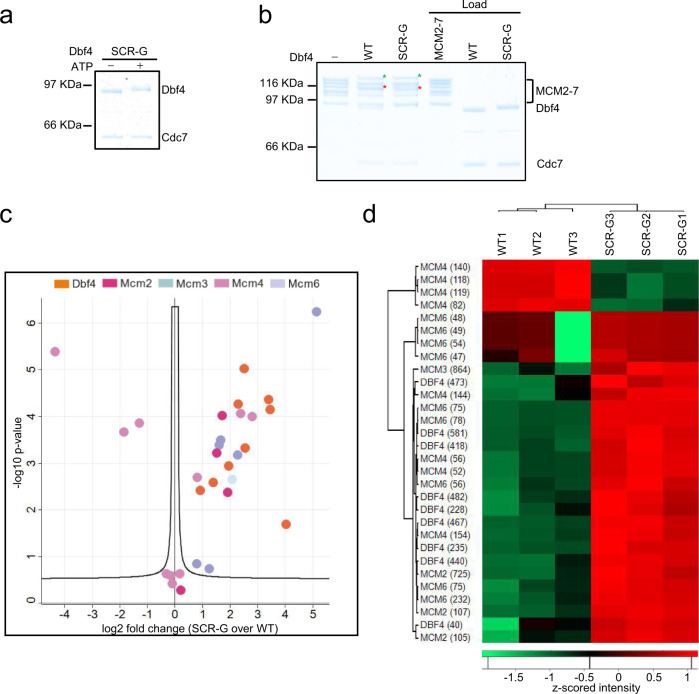
Biochemical analysis of the Dbf4 substrate coordinating region (SCR). **a** Dbf4 autophosphorylation analysis of Dbf4 SCR-G. Similar results were obtained in two independent experiments. **b** The Dbf4 SCR-G mutant was analysed for its interaction of DDK with MCM2-7 DH. 150 nM DDK was used in the reaction. Similar results were obtained in three independent experiments. **c** Volcano plot comparing WT and SCR-G DDK phosphorylation of the MCM2-7 DH. Two-sample Student’s *t*-test carried out with three replicate intensities considered per group. *P*-values were corrected for multiple comparisons to an FDR of 0.05 (permutation-based FDR). **d** Volcano plot significant phosphosites visualised using HCA coupled to a heatmap of z-scored site intensities.

As we observed that the Dbf4 SCR region was cladding part of the cleft that lines the active side of Cdc7, we generated a mutant to address the specific function of this region. We replaced Dbf4 aa 509–538 with a 30 aa glycine/serine linker sequence. The mutant was competent for Dbf4 autophosphorylation (Fig. 5a) and MCM2-7 phosphorylation (Fig. 5b), although we observed changes in the phospho-shift of Dbf4 and Mcm4. Phospho-proteomic analysis revealed substantial changes in the phosphorylation of Mcm4, Mcm2, Mcm6 and Dbf4 (Fig. 5c, d and Supplementary Table 1). Overall, in the SCR mutant, we observed reduced phosphorylation of Mcm4 and enhanced phosphorylation of Dbf4, Mcm2 and Mcm6, although a few specific sites displayed the opposite behaviour. The effect was not the same for all sites, arguing that the SCR region modulates the selection of DDK phosphorylation sites.

### Conformational flexibility of the MD-(ATPγS) complex

**Fig. 6:**
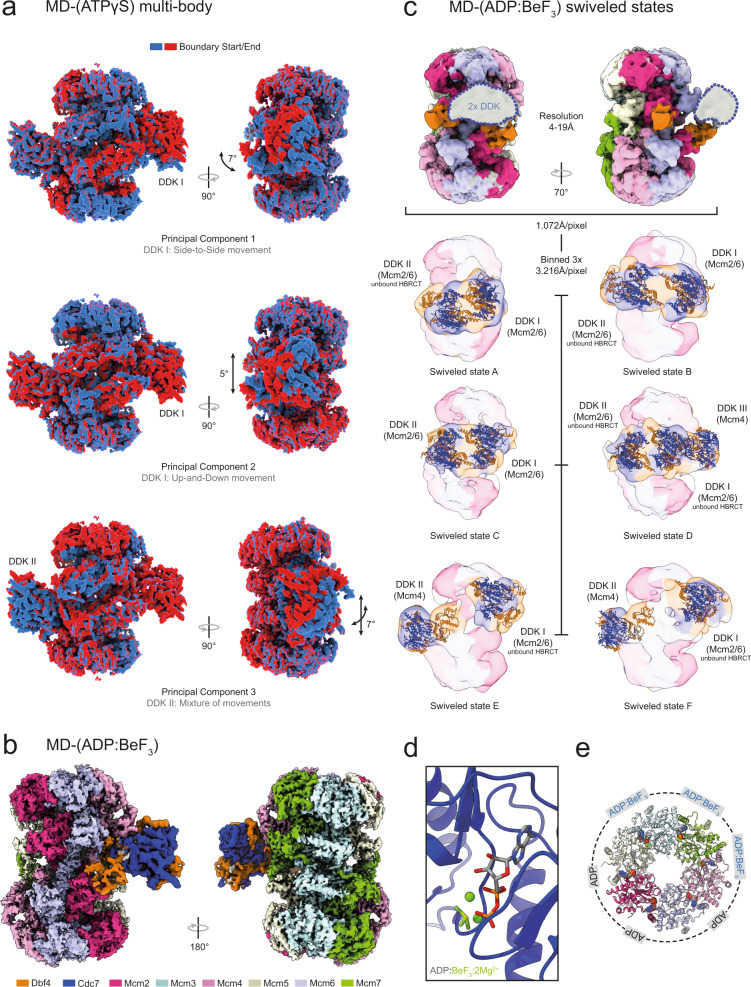
DDK dynamics revealed through multi-body refinement and flexible analysis and alternative MD complex conformations in the presence of ADP:BeF_3_. **a** Flexible analysis of the multi-body refinement of MD-(ATPγS) state III shows distinct movements of DDK relative to the MCM2-7 DH. In addition to DDK movement, the DH C-terminal domain also shows rotational movements. **b** Side and top views of the cryo-EM composite map (see Methods) of the MD complex in the presence of ADP:BeF_3_. DH at 3.8 Å mean resolution and DDK at 4.4 Å mean resolution. **c** The alternative swiveled structural states of the MD complex in the presence of ADP:BeF_3_. The EM map derived from the unbinned data displayed density large enough to fit two DDK subunits in a swiveled conformation, but the data had to be Fourier binned 3 × 3 to obtain easier to interpret EM maps. The data revealed a range of DDK binding modes, that feature in some cases either or both Mcm2/6 and Mcm4 targeted DDK conformations. The Mcm subunit which is targeted by each DDK is labelled. In some states, the HBRCT domain of Dbf4 is not bound to Mcm2. The local resolution EM maps of the different MD-(ADP:BeF_3_) swiveled states are shown, coloured according to the key shown in (**b**). The atomic model of DDK/Dbf4-HBRCT, derived from the map shown in (**b**), was manually docked into the EM maps. **d** Zoomed view of the Cdc7 active site bound to ADP:BeF_3_ and 2 Mg^2+^ ions. **e** Overview of the nucleotide occupancy and type in each Mcm subunit within the MD-(ADP:BeF_3_) complex.

The MD-(ATPγS) state III 3D-refined map suffered from conformational heterogeneity, particularly in regions corresponding to DDK. To alleviate this, we employed the use of multi-body refinement and flexibility analysis. The multi-body refinement improved the MD-(ATPγS) map significantly and allowed us to determine three different principal component movements characterised by side-to-side, up and down and a mixed-mode motion (Fig. 6a, Supplementary Fig. 7 and Supplementary Movie 2). These alternative structural states could reflect the coupling of DDK to Mcm4 through different interaction motifs or the tracking of the kinase along the Mcm4 tail in order to reach alternative sites for phosphorylation. Moreover, this binding mode involving multiple interaction sites is probably important for supporting the eventual release of the kinase from the helicase complex.

### Mechanism of Mcm subunit phosphorylation by DDK

The MD-(ATPγS) structure can readily explain how Cdc7 can reach the Mcm4 N-terminal extension and phosphorylate it, as the Mcm tail is in direct proximity to the kinase. However, DDK also phosphorylates Mcm2 and Mcm6, which are distant from the observed binding site. We wondered whether alternative structures could exist and therefore screened ATP analogues for different conformational states (Supplementary Fig. 1d). We discovered that in the presence of ADP-BeF_3_, DDK adopts two different conformations on the DH, namely MD-(ADP-BeF_3_) state I and MD-(ADP-BeF_3_) swiveled state, in a ~70/30 distribution, respectively (Fig. 6b, c; Supplementary Fig. 8). The first conformation is similar to conformer II of MD-(ATPγS), where only one DDK is bound to the MCM2-7 DH (Figs. 6b and 1b). In the alternative swivel state of ADP-BeF_3_, DDK is bound to the helicase predominantly via its Mcm2 N-terminal section, with the main body of DDK being rotated by 180 degrees, repositioning Cdc7 just above the Mcm6 and Mcm2 N-terminal regions (Fig. 6c). In this Dbf4 swivel state (Fig. 6c; swivel state A, B, C and D), two DDK molecules are observed in the proximity to each other. Many kinases employ dimerization via their kinase domain^50^ and genetic, biochemical and structural data highlight a Cdc7 self-interaction ability^40,51,52^. However, our structural data were not of sufficient resolution (local resolution of DDK between 11 and 25 Å) to prove Cdc7 dimerization unambiguously. Moreover, we observed additional structural states with mixed conformations (Fig. 6c; swivel state E and F), one where DDK adopts a conformation compatible with Mcm4 phosphorylation, while the other adopts the Mcm2/6 phosphorylation state. Another conformer indicated the presence of more than 2 DDK molecules, which could become recruited via Mcm4, Mcm2 and Cdc7 dimerization (Fig. 6c; swivel state D). Interestingly, this is reminiscent of recent single-molecule results, which indicated that up to six GINS become recruited to the MCM2-7 DH during helicase activation^53^. In summary, the observed structural states highlight an unexpected plasticity of the DDK kinase, with the HBRCT domain representing an anchorage point, and hinge that acts together with a DDK dimerization surface to support interactions between Cdc7 and the N-terminal tails of Mcm2/6 or Mcm4 (Fig. 6c).

### Structural variant of MD bound to DNA

In order to understand whether a natural adenosine nucleotide would promote a different DDK-MCM2-7 organisation, we assembled complexes with ATP (Supplementary Fig. 1b, e). Moreover, we crosslinked the complex immediately after release from the magnetic beads. This procedure fixes complexes at a very early time point and stops DNA sliding out of the MCM2-7 DH, but results in a slightly higher level of protein aggregation and therefore a lower concentration of the final complex. Due to the low particle number, the resolution of the map was limited to 9.1 Å, however, the MCM2-7 DH structure was nearly identical to MD-(ATPγS) (Supplementary Figs. 9 and 10). Early fixation of the MD ATP complex prevented DNA sliding out of the central MCM2-7 channel and allowed us to detect DNA within the MD ATP complex (Supplementary Fig. 10b), however, the presence of DNA did not significantly alter the overall DH complex conformation, consistent with previous observations (Supplementary Fig. 10a)^45,54,55^. Therefore, the MD-(ATP) DNA structure strongly suggests that the MD-(ATPγS) structure reflects the true conformation of the DDK-MCM2-7 complex. Interestingly, multibody analysis of MD-(ATPγS) and MD–(ATP) revealed similar side-to-side and up-and-down movements of DDK. However, in MD-(ATP) we also observed a more dramatic rotation of the MCM C-terminal ATPase domain, which might reflect MCM2-7 ATP-hydrolysis (Supplementary Fig. 7), which has previously been observed in the context of the CMG complex^56^. As the DH is not capable of ATP-hydrolysis^45,46^, the observed changes could be due to loss of DNA in a subfraction of MD-(ATP) molecules.

### MD structures reveal a structural change near the Mcm2/5 gate

To our surprise, in the MD-(ATPγS) structure we observed an ATPγS molecule bound to the Oligonucleotide/Oligosaccharide-Binding (OB)-fold interface of Mcm2/6, a non-canonical nucleotide-binding site (Supplementary Fig. 11a). This pocket has a positive charge and could therefore represent a potential DNA binding site. Docking of double-stranded (ds) and fork DNA from the MCM2-7 DH^45^ and CMG^57^, respectively, indicate proximity to ds DNA (Supplementary Fig. 11b). This indicates that the positively charged surface may attract the ATPγS molecule, although it could also have a role in channelling ATP into the helicase motor.

**Fig. 7:**
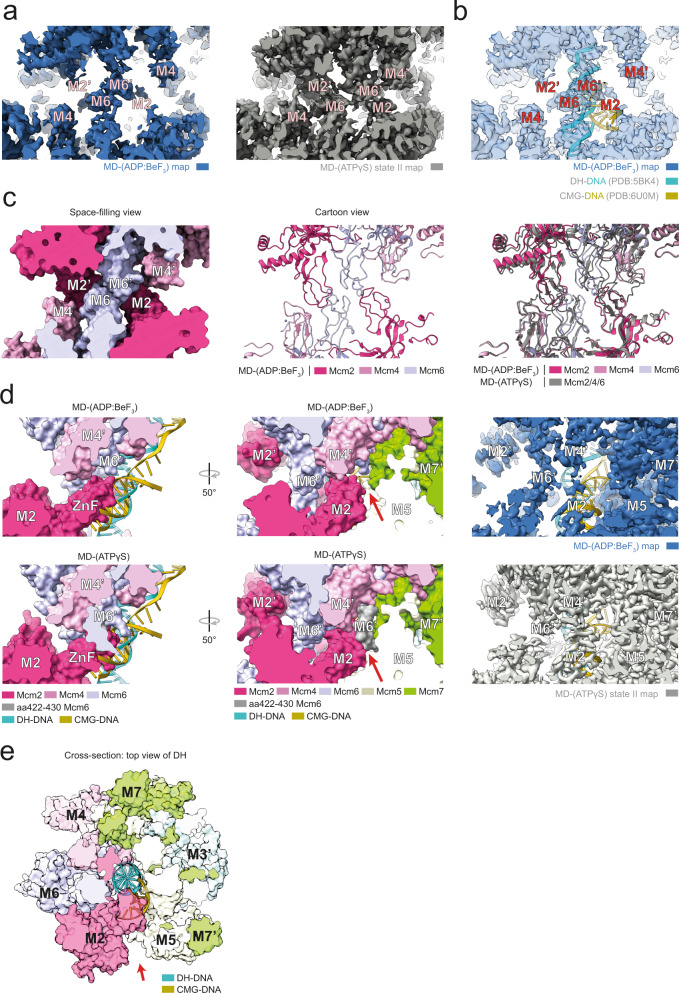
Analysis of structural differences within the MD complex in the presence of different nucleotides. **a** Zoomed view of the DH central region and comparison between MD-(ADP:BeF_3_) state I and MD-(ATPγS) state III cryo-EM maps. The MD-(ADP:BeF_3_) map displays stronger density at the Mcm4/6 zinc finger domains and weaker density at the Mcm2 zinc finger domain compared to MD-(ATPγS). **b** MD-(ADP:BeF_3_) state I map and DNA from DH and CMG docked structures. **c** Comparison between MD-(ADP:BeF_3_) state I and MD-(ATPγS) state III atomic models. The models show global structural differences at Mcm2/4/6 zinc finger domain regions. **d** Zoomed view of the Mcm2:Mcm5 interface and comparison between MD-(ADP:BeF_3_) and MD-(ATPγS) atomic models. A short Mcm6 region (aa422-430) blocks this channel in the MD-(ATPγS) and is disordered in MD-(ADP:BeF_3_) leaving a 15-30 Å channel opening. **e** Cross-section of the MD-(ADP:BeF_3_) atomic model showing the trajectory of DH and CMG DNA between Mcm2:Mcm5. **d**, **e** Red arrows indicate channel opening region.

In the MD-(ADP-BeF_3_) structure we did not observe non-canonical nucleotide binding, however, in comparison to MD-(ATPγS), an alternative canonical nucleotide-binding state was observed. ADP-BeF_3_ was only present in three Mcm ATP binding pockets, while ADP was seen at the Mcm5/2, Mcm2/6 and Mcm6/4 interfaces (Fig. 6d, e). This suggests that in our MD-(ADP-BeF_3_) structure, three interfaces adopt a post ATP-hydrolysis state. This structural change had an allosteric effect on the MCM2-7 double-hexamer interface: we observed that the Mcm6 zinc finger of one hexamer was reorienting to the Mcm6 zinc finger of the opposite double-hexamer (Fig. 7a–c). This generated a small gap at the Mcm2/5 interface (Fig. 7d). However, when comparing the cryo-EM maps of the MD-(ADP-BeF_3_) and MD-(ATPγS) using similar thresholds (Fig. 7a, b), one can additionally observe a reorganisation of the Mcm2 zinc finger, which adopts a more flexible conformation and therefore is only visible at a lower threshold.

Interestingly, by docking dsDNA of the MCM2-7-DH and forked DNA of the CMG into the MD-(ADP-BeF_3_) complex one can observe that the lagging strand of the CMG-fork DNA can almost pass through the Mcm2/5 gate, but is clashing with the flexible Mcm2 zinc finger (Fig. 7d, e). While our previous work had identified that the lagging strand in the context of the DH is pressed against the Mcm2/5 interface^56^, our data here suggest that the Mcm ATP/ADP binding state is communicated allosterically with the DH-interface, inducing a reorganisation of Mcm6 and Mcm2 zinc fingers that may occur during helicase activation and could support the escape of single-stranded DNA through the Mcm2/5 gate.

### Molecular dynamics analysis reveals the binding mode of an extended Mcm4 N-tail

**Fig. 8:**
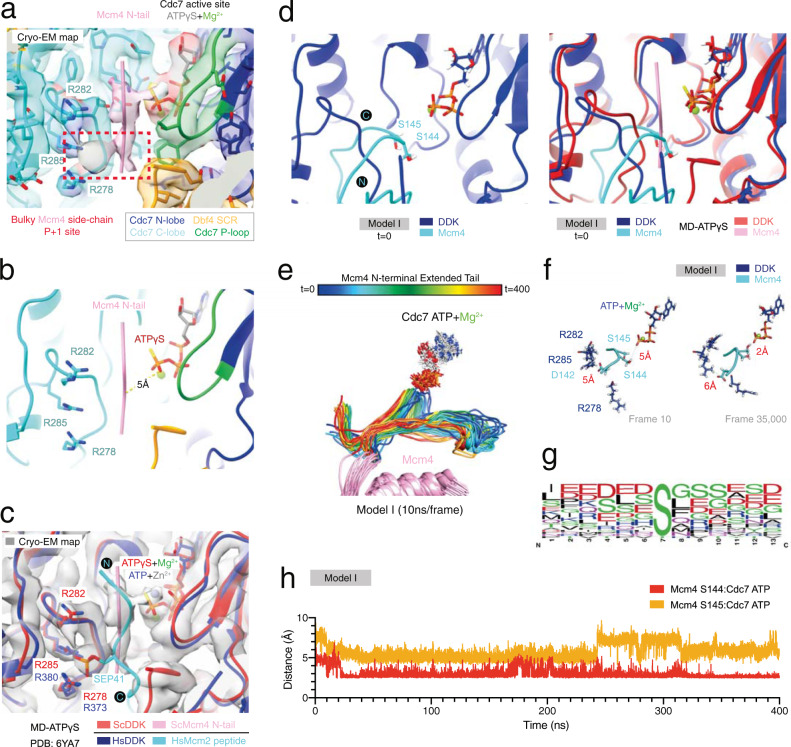
Molecular dynamics simulation reveals the binding mode of an extended Mcm4 N-terminal tail substrate at the Cdc7 active site. **a** Zoomed view of the MD-(ATPγS) state III atomic model DDK active site and associated cryo-EM density map. The cryo-EM map features density reminiscent of a bulky Mcm4 tail residue side chain at the P + 1 site. This Mcm4 tail unknown residue is also surrounded by Cdc7 R278, R282 and R285. **b** Simplified view of (**a**) displaying the distance measurement between the Mcm4 N-terminal tail (backbone CA atom) and Cdc7 ATPγS (O3G atom). **c** Overlay of the core of human DDK (PDB:6YA7) and budding yeast DDK atomic models. The position of the phospho-serine residue (SEP) in the human Mcm2 peptide matches with the bulky residue side chain density position observed in the MD-(ATPγS) cryo-EM map. **d** Zoomed view of the DDK active site of Model I (molecular dynamics starting model I, featuring an extended Mcm4 N-terminal tail) and comparison with the MD-(ATPγS) atomic model. **e** Snapshots of model trajectory, sampled every 10 ns, during a 400 ns GROMACS molecular dynamics simulation. The successive order of the models is represented by rainbow colours (red to blue). The long flexible Mcm4 tail adopts multiple conformations throughout the simulation. **f** Simplified view of the DDK active site, showing the distance measurements between the nearest DDK Mcm4 target residue (S144, OG atom) and Cdc7 ATPγS (O3G atom) and Mcm4 D142 (CG atom) at the P-2 position and R285 (NE atom). **g** Sequence logo, generated using WebLogo 3, displaying the frequency (N) of different amino acid residues at atypical human DDK target sites^1^. The residues preceding the target DDK residue feature in a high proportion of cases acidic residues (D/E). **h** Plot of distance measurement between the Mcm4 N-terminal tail (backbone CA atom) and Cdc7 ATPγS (O3G atom) throughout the entire simulation.

Although our cryo-EM structures failed to visualise large sections of the flexible Mcm4 N-tail, we were able to determine the position of a short region of the Mcm4 tail at the Cdc7 active site and found that the residue at P + 1 featured density reminiscent of a bulky side chain (Fig. 8a–c). Intriguingly, the residue at the P + 1 is in close proximity to form contacts with Cdc7 R278, R282 and R285. The use of in silico modelling and molecular dynamics simulations, together with our cryo-EM findings, have the potential to allow us to assess the mode of binding of an extended Mcm4 N-tail and explore further DDK function. Therefore, we modelled the Mcm4 N-tail (aa. 134–176) ab initio using Rosetta^58^ and then performed molecular dynamics simulations using three different trajectory Mcm4 N-tail starting models (I-III) (Supplementary Figs. 12 and 13; Supplementary Movie 3–5). The three models were chosen based on proximity of the Mcm4 tail with the DDK active site and the tail making minimal contact with core regions of Mcm4 and Mcm6 (Supplementary Fig. 12b, c). As expected, the N-terminal section of the tail of each model displayed great flexibility during the 400 ns simulation (Supplementary Fig. 12d). Out of the three models, model I featured the tail closest to the Cdc7 active site and featured distances favourable for phosphorylation (Fig. 8d–h). The other models (II-III) formed close contacts with DDK but failed to reach a stable conformation near the Cdc7 active site within the time window (Supplementary Fig. 13e–g). Furthermore, model I positioned DDK target serine 144 towards the ATP molecule and satisfied the requirement for the presence of an acidic residue neighbouring the target serine, surprisingly, via D142 (located at P-2) (Fig. 8f). The position of an acidic residue preceding target DDK sites could explain how DDK targets atypical sites that do not feature acidic residues succeeding the target residue. Indeed, a large proportion of atypical DDK target sites do feature acidic residues preceding the target residue (Fig. 8g)^1,12^. In summary, the molecular dynamics data further agree with the positioning of the Mcm4 N-tail based on the cryo-EM data. The data also indicates that the flexibility of the Mcm4 tail alone may not be sufficient for substrate binding and provides a model for target selection of DDK atypical sites.

## Discussion

Kinases frequently form fleeting interactions with their substrates. DDK is unusual, as it forms a stable complex with MCM2-7 DH, which supports efficient phosphorylation of N-terminal tails of Mcm2, Mcm4 and Mcm6^12,13^. The DDK–MCM2-7 interactions stabilise flexible regions in Dbf4 and Cdc7, which greatly supported the structural analysis of the overall kinase complex and allowed us to describe the dynamic substrate engagement in immense detail.

Our biochemical and structural analysis revealed how DDK kinase becomes recruited to the helicase through the Dbf4 HBRCT domain, which anchors on Mcm2 (Figs. 1a–c, 2c and 6). Recently, it was shown that the unstructured tail of Mcm2 is also required for DDK recruitment^14,15^, but the mechanism is still unclear. Although, it is not known which part of Dbf4 binds to N-terminal tail of Mcm2, we speculate that unstructured sections of Dbf4 could be involved in binding to the Mcm2 tail, as protein interactions between unstructured domains are ideal to promote initial contact between protein complexes^59^. As such we suggest that the unstructured tail acts as an initial contact, which consequently becomes complemented or replaced by the Mcm2-HBRCT-Dbf4 interaction. This dual recruitment mechanism can potentially explain why deletion of the Dbf4 HBRCT domain is lethal in embryonic mouse cells^60^ and not in yeast^44^.

**Fig. 9:**
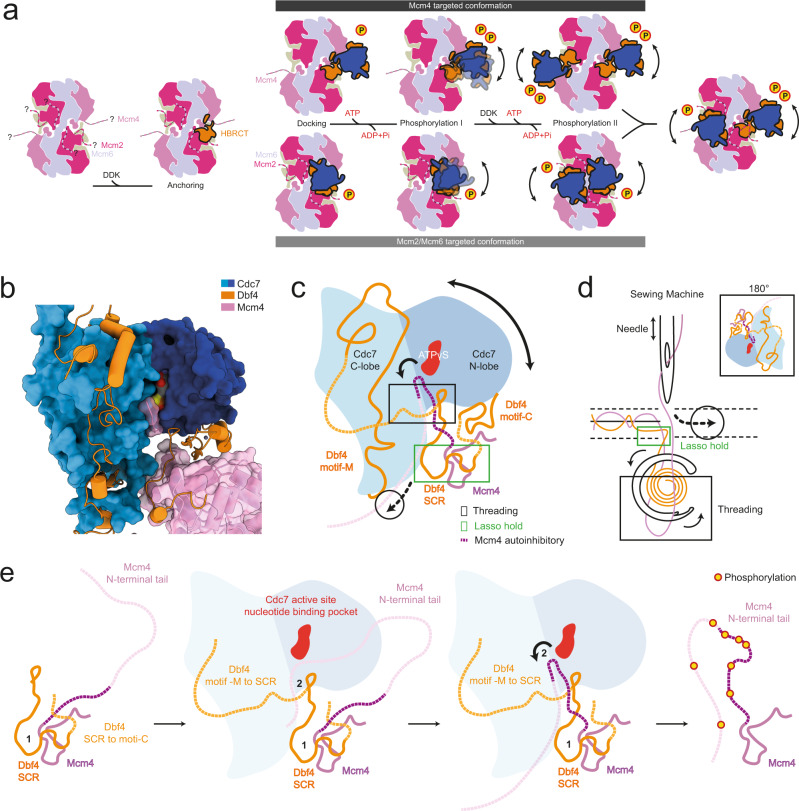
Mechanism of DDK substrate localisation and processive phosphorylation of multiple Mcm N-terminal tails. **a** Schematic illustration of the different stages of DDK recruitment to the MCM2-7 DH and different modes of binding. **b** DDK bound to the Mcm4. **c** Cartoon drawing of (**b**), with missing structural regions extended as dotted lines. DDK forms a lasso around the most N-terminal resolved region of Mcm4. The missing region between Dbf4 motif-M and Dbf4 SCR traps the Mcm4 flexible tail, encircling it at or close to the Cdc7 active site. **d** DDK is likened to a sewing machine. The main principle being that Mcm4 gets threaded by DDK, and this process allows the kinase to reach the most C-terminal end of the flexible tail. **e** Simplified version of (**c**) showing a step-wise hypothetical mechanism of Mcm4 substrate localisation and processive phosphorylation. The region labelled 1, features a part of Dbf4 SCR which forms a hook (lasso) around the rigid surface of Mcm4. The region labelled 2, features an encircled Mcm4 flexible tail that gets threaded through the kinase. The missing structural regions are represented as dotted lines and resolved regions as solid lines.

So far it was unknown how DDK docking could support Mcm2, Mcm4 and Mcm6 phosphorylation. Here, we speculate that a hinge region between the HBRCT and the other sections of DDK allows flexible engagement of Mcm4 or Mcm2/6 (Figs. 6c and 9a). These interactions are characterised by patches of weak contacts (Fig. 4a), which support several alternative swivel states (Figs. 6c and 9a), but may also reflect tracking of phosphorylation sites along the Mcm N-terminal tails.

Specific DDK dependent phosphorylation of the MCM2-7 DH is crucial to direct limiting DDK amounts to replication origins that need to be activated at a given time point. Our structural data now provide insights into how DDK targets the MCM2-7 DH over the single hexamer. Specifically, the MD ATPγS structure revealed that docking of DDK via the Mcm2-HBRCT-Dbf4 interaction surface on hexamer 1 directs Cdc7 kinase activity to Mcm4 of hexamer 2 (Fig. 1d). On the other hand, in the swivel state, Mcm2 docking directs phosphorylation to Mcm2 and Mcm6. Here, trans-hexamer phosphorylation will depend on either Cdc7-dimerisation, which was previously observed^40,51,52^, or the orientation of the Cdc7 active site to the hexamer opposite of its docking site. However, due to the low resolution of the swivel state MD complexes, we cannot fully conclude if one or both of these mechanisms are used. As such, our data provide the structural basis for DDK’s preference for phosphorylating the MCM2-7 double-hexamer over the single-hexamer at the Mcm4 N-terminus and provide two possible mechanisms for specific trans-hexamer phosphorylation of the Mcm2 and Mcm6 N-termini.

Binding across the double-hexamer interface, may also suggest that splitting of the DH during helicase activation may be required to release DDK^46^. This would connect helicase activation to DDK release and therefore limit DNA replication. Moreover, if DDK stays attached to the MCM2-7 DH and partially covers the Mcm4-N-terminal tail, this could shield the Mcm4 tail from phosphatases, which are known to negatively regulate replication initiation, and in this way suppress the auto-inhibitory activity of the Mcm4 tail.

It is clear that Mcm2-HBRCT has a positive role in DDK binding. In this context, it is of interest that Rad53 also binds to the Dbf4-HBRCT domain, which could destabilise the MD complex^3,15,52^. Moreover, Sld3 binds both the Mcm2 N-terminus and the Dbf4 HBRCT^61^, which could further regulate MD stability. Besides the Dbf4 HBRCT hotspot for protein interactions, the Dbf4 C-terminal section has been shown to bind the transcription factor Fkh1^62^, which could promote early activation at a subset of origins, potentially by stabilising the MD complex.

In the presence of DDK, MCM2-7 itself appears relatively static, fitting with the observation that the MCM2-7 DH phosphorylated by DDK has no large conformational changes when compared to the unphosphorylated DH^54^. We did not observe the inhibitory domain of Mcm4 (aa74–174), which becomes relieved upon DDK dependent MCM2-7 phosphorylation, so we cannot conclude on its structure^24^. Surprisingly, with ADP-BeF_3_ we observed a structural change in the double-hexamer interface, with the Mcm6 zinc finger becoming repositioned and the Mcm2 zinc finger adopting greater flexibility, resulting in a gap near the Mcm2/5 gate (Fig. 7). However, our data show that the Mcm6 zinc finger can still restrain initial DNA unwinding (Fig. 7e). Mcm10, which is known to induce initial DNA unwinding, has been shown to bind specifically to the Mcm2 and Mcm6 N-terminal domains^63,64^ and therefore we speculate that Mcm10 could support further reorganisation of the Mcm2 and Mcm6 zinc fingers to induce initial DNA unwinding. As such, the MD-(ADP-BeF_3_) structure may emulate changes that occur during the assembly of the replication fork and hint at a potential DNA unwinding mechanism.

The stable DDK-helicase interaction greatly supports the efficient and regulated phosphorylation of MCM2-7. The MD structure and the biochemical analysis revealed several features that explain how the phosphorylation process works. In addition to the HBRCT domain, which we discussed above, we observed that a yeast specific long insertion in Dbf4 between its motif-M and motif-C contains a structured section, which is cladding the path between the active site of Cdc7 and the last resolved N-terminal section of Mcm4. The biochemical and proteomic analysis of a mutant, where the region was replaced with a flexible linker, showed a significantly altered phosphorylation profile (Fig. 5c, d). As such, the name “substrate coordinating region” is fitting for this domain. The unstructured section N-terminal to the SCR is enriched for DDK phosphorylation sites, suggesting that it may have a regulatory role. In addition, we noticed the unusual structural configuration of Cdc7, Dbf4 and Mcm4. Here the Mcm4 tail is threaded through a Dbf4 loop, which may help, together with the SCR, to restrain and guide the Mcm4 N-terminus during the phosphorylation process (Fig. 9b–e). This speculative mechanism may explain how the Mcm4 N-terminal tail becomes phosphorylated in a more efficient manner. In addition, the molecular dynamics simulation indicated that the flexibility of the N-tail of Mcm4 alone is not sufficient for DDK substrate searching, adding more weight to the observed DDK global movements (Fig. 6 and Supplementary Fig. 7), which will be required for DDK to find its substrate in a timely manner. The simulation also reiterates the importance of a negatively charged acidic residue near the target serine and explains how even a preceding acidic residue to the target site might help achieve phosphorylation.

Finally, as the DDK-MCM2-7 interface is characterised by weak protein-protein interactions, it appears possible to use the new information to inhibit DDK phosphorylation of the MCM2-7 DH by small molecules that block the interaction between helicase and kinase. In contrast to a Cdc7 kinase inhibitor, this mode of inhibition may allow increased specificity for the inhibition of helicase activation.

## Methods

### Protein purification

ORC, Cdc6, Cdt1, and MCM2-7 were purified as previously described^23^. Plasmids generated in this study are described in Supplementary Table 4. For expression of the Dbf4 mutants, the plasmids were generated by GenScript.

### DDK purification

DDK was expressed in budding yeast cells as previously described^65^. Cell powder was resuspended with 1 volume of buffer D (50 mM HEPES/KOH pH 7.5, 1 mM EDTA, 1 mM EGTA, 10% glycerol, 0.02% NP40, 1 mM DTT) and 0.4 M NaCl (buffer D + 0.4 M NaCl) and protease inhibitor cocktail without EDTA (Sigma, S8830-20TAB) and centrifuged at 27,600 × *g* for 1 h at 4 °C. The supernatant was incubated with amylose resin (NEB, E8021L) for 2 h. Following this, the resin was washed: with buffer D + 0.4 M NaCl, with buffer D + 1.0 M NaCl, and with buffer D + 0.2 M NaCl, and then the resin was incubated with lambda phosphatase (NEB, P0753S) in buffer D + 0.4 M NaCl + 1 mM MnCl_2_ for 1 h at 4 °C. The MBP tag was cleaved with PreScission Protease for 1 h and the eluate was applied to a HiTrap Heparin column and then a Superdex 200 gel filtration column pre-equilibrated with buffer D + 0.4 M NaCl.

### DH-DDK interaction assay

MCM2-7 DH was prepared using 10xARS DNA as previously described^45^. 80 nM ORC, 160 nM Cdc6, 160 nM Cdt1, and 160 nM MCM2-7 were assembled on 7.2 nM 10xARS DNA-beads for 1 h at 24 °C in pre-RC buffer (25 mM HEPES/KOH pH 7.5, 100 mM KGlu, 10 mM MgAc, 50 μM ZnAc, 3 mM ATP, 5 mM DTT, 0.1% Triton X-100, and 10% glycerol). After high salt washes with pre-RC buffer + 300 mM NaCl, 400 nM DDK, 150 nM DDK for proteomics, or DDK as indicated in the figures was added to the mixture and incubated for 30 min at 24 °C. The beads were washed with pre-RC buffer and the proteins still bound to DNA were eluted by DNase I. The eluate was analysed by SDS-PAGE stained with silver or coomassie blue. The SDS-PAGE results were used to determine DH-DDK interaction.

### MD-(ATP) sample preparation

MCM2-7 DH was prepared as described above. The DH was phosphorylated by 400 nM DDK for 30 min at 24 °C in pre-RC buffer. After washing with pre-RC buffer, the DH-DDK complex was eluted by AluI (NEB, R0137L) in buffer C (25 mM HEPES/KOH pH 7.5, 5 mM MgAc, 100 mM KAc) + 5% pre-RC buffer + 3 mM ATP. The eluate was crosslinked with 0.1% glutaraldehyde (agar scientific, R1020) for 15 min on ice.

### MD-(ATPγS) and MD-(ADP-BeF_3_) sample preparation

MCM2-7 DH was prepared as described above. After the DH was eluted by AluI, 250 nM DH was incubated with 750 nM DDK for 10 min at 24 °C in buffer C containing 5% pre-RC buffer and 3 mM ATPγS or ADP:BeF_*x*_ (3 mM ADP, 15 mM NaF and 3 mM BeCl_2_). The sample was applied to a 15–45% glycerol, 0–0.1% glutaraldehyde and 0–3 mM nucleotide (ATPγS or ADP:BeF_*x*_) gradient in buffer C. In parallel, the sample was also applied to a gradient without glutaraldehyde for SDS-PAGE analysis. The sample was centrifuged at 48,477 × *g* (20,000 rpm using an SW 55 Ti rotor (Beckman)) for 16 h at 4 °C and then 32 μl fractions were manually collected. The fractions containing the MD complex were pooled, and glycerol was removed from the sample using a Microcon DNA Fast Flow Centrifugal Filter Unit (Merck).

### Cryo-EM grid preparation

Prior to cryo-EM grid preparation all samples, or complex containing sample fractions from glycerol gradients, were checked by negative stain-EM to confirm good particle number and particle homogeneity. The cryo-EM grids were all prepared by applying 3.5 μl of the sample onto glow discharged (15 mA for 25 sec), 2 nm amorphous carbon-coated, Quantifoil R2/2 300-mesh copper grids or, for MD-(ATP), Quantifoil R1.2/1.3 300-mesh copper grids. The sample was allowed to absorb onto the grid for 30 s at 4 °C and 100% humidity, before being blotted for 1.5 s with a blot force of +2 and plunged into liquid ethane cooled by liquid nitrogen using an FEI Vitrobot Mark IV. The grids were stored in liquid nitrogen until imaging.

The cryo-EM grids were loaded into Titan Krios electron microscopes (Thermo Fischer Scientific) equipped with a field emission gun and a direct electron detection (DED) camera. The microscopes were operated at 300 kV and images were acquired using EPU automated data collection software. The MD-(ATPγS) data were collected using a K3 DED (Gatan, Inc.) in electron-counting super-resolution mode at a nominal magnification of ×81,000 and a final pixel size of 0.530 Å using defocus values ranging between −1.0 and −3.0 μm and imaging 3 shots per hole. A total of 9909 multi-frame movies were recorded, each with a total dose of 45.9 electrons/Å² over 54 frames. The MD-(ADP:BeF_3_) data were collected using a K3 DED (Gatan, Inc.) in electron-counting super-resolution mode at a nominal magnification of ×81,000 and a final pixel size of 0.536 Å using defocus values ranging between −1.7 and −3.7 μm and imaging 3 shots per hole. A total of 13,470 multi-frame movies were recorded, each with a total dose of 48.4 electrons/Å² over 40 frames. The MD-(ATP) data were collected using a Falcon 3EC DED (Thermo Fischer Scientific) in electron-counting mode at a nominal magnification of ×75,000 and a pixel size of 1.085 Å using defocus values ranging between −1.6 and −3.2 μm and imaging 2 shots per hole. A total of 3416 multi-frame movies were recorded, each with a total dose of 50.0 electrons/Å² over 19 frames. A summary of the cryo-EM data collection settings used is shown in Supplementary Table 5.

### Cryo-EM image processing

MD-(ATPγS)—The cryo-EM super-resolution multi-frame movies of the MD-(ATPγS) complex were motion corrected, dose weighted, Fourier binned 2 × 2 (1.06 Å/pixel) and summed using MotionCor2^66^. The micrographs were gain-corrected using a gain reference file (flipped upside down) and the frame alignment was performed using a B-factor of 150, averaging in groups of 3 frames and in patches (X, Y) of 5 × 5. CTF estimation was then performed on non-dose weighted summed micrographs using CTFFIND4^67^. Micrographs were then inspected; crystalline ice contaminated micrographs were discarded and only micrographs which featured thorn rings at 5 Å or better were used for further processing. A set of 3000 particles were manually picked and used to generate reference-free 2D class averages for reference-based auto-picking. A total of 2,572,593 particles were automatically picked from 9772 dose-weighted micrographs using RELION^68^. The particles were Fourier binned 3 × 3 (3.18 Å/pixel) and extracted with a 120-pixel particle box size. The particles were subjected firstly to reference-free 2D classification (*K* = 50, *T* = 2) without CTF to remove ethane-like contamination and large junk particles, followed by another round of 2D classification (*K* = 25, *T* = 2) with CTF. The 2D class averages selected (containing 1,633,199 particles) showed secondary structure features for the DH core and blurred regions that matched the expected size and shape of DDK. A sub-set of 370,560 particles were separately extracted, subjected to several rounds of 2D classification, ab initio model generation, several rounds of 3D classification and 3D auto-refinement. The final map (at a mean resolution of 6.5 Å (FSC = 0.143)) derived from the sub-set of particles was low-pass filtered by 30 Å and used as the input initial reference model for the complete particle set. The particles were subjected to 3D classification (*K* = 12, *T* = 3) and the data was split three ways. The classes that displayed particles which featured the DH core with only one DDK were grouped together, in total these contained 258,684 particles and represent the input particles used for MD-(ATPγS) state I processing. The classes that displayed particles which featured one detailed DDK bound to the DH were grouped together, in total these contained 138,647 particles and represent the input particles used for MD-(ATPγS) state II processing. The classes that displayed two or partially two DDK molecules bound to the DH were group together, in total these contained 1,235,868 particles and represent the input particles used for MD-(ATPγS) state III processing.

The MD-(ATPγS) state I grouped particles were subjected to 3D classification (*K* = 5, *T* = 4) and the class with the most detailed structural map features, which contained 55,655 particles, was selected. The particles were then subjected to 3D auto-refinement, re-centered and re-extracted with a pixel size of 1.06 Å and a 360-pixel particle box size and then an additional round of 3D auto-refinement was performed. The 3D auto-refined particles were then used for per-particle CTF refinement and Bayesian polishing and this was followed by 3D auto-refinement. The particles were then subjected to a final round of 3D classification (*K* = 3, *T* = 4), in which two of the selected 3D classes, containing 12,800 particles, featured detailed and similar structures. The models displayed weak density for the core of DDK but showed strong density for the HBRCT domain of Dbf4 (bound to the surface of Mcm2). The particles were then subjected to 3D auto-refinement (C1 symmetry), yielding a 7.1 Å mean resolution map (FSC = 0.143 cut-off). Local resolution estimation of each map was then determined using the last iteration of split output of unfiltered half-maps as input in RELION.

The MD-(ATPγS) state II grouped particles were subjected to 3D classification (*K* = 5, *T* = 4) and two classes, containing 56,530 particles, which displayed detailed structural map features for the DH, were selected. The particles were then subjected to 3D classification (*K* = 5, *T* = 4) and surprisingly some of the classes started to display detailed structural map features for one DDK. The classes which displayed strong density for DDK were selected and subjected to 3D auto-refinement. The 3D auto-refined particles were then re-centered and re-extracted with a pixel size of 1.06 Å and a 360-pixel particle box size and an additional round of 3D auto-refinement was performed. The particles were subjected to multiple rounds of 3D refinement to improve the density of DDK until secondary structure features could be observed. 3D auto-refinement (C1 symmetry) was performed, using 38,012 particles, yielding a 4.5 Å mean resolution map (FSC = 0.143 cut-off) which featured distinguishable secondary structure elements for the DH and DDK. The 3D auto-refined particles were then used for per-particle CTF refinement and Bayesian polishing and this was followed by 3D auto-refinement. The refined map was used to generate a soft mask around the well-resolved DDK (extend: 12 pixel, soft-edge: 6 pixel) and this was used to perform masked 3D classification (*K* = 5, *T* = 4) without image alignment. The classes (containing 22,459 particles) which displayed detailed map features for DDK were selected and the particles were subjected to a final round of 3D auto-refinement (C1 symmetry), yielding a 4.3 Å mean resolution map (FSC = 0.143 cut-off). Notably, at lower threshold levels the MD MD-(ATPγS) state II map displayed density at the second DDK region for only the HBRCT domain of Dbf4 and not the core of DDK. To further reduce the conformational heterogeneity of DDK, the map was segmented into two bodies, one encompassing the well-resolved DDK and the other the DH core. The auto-refined particles were subjected to 3D multi-body refinement and flexible analysis using two soft masked bodies (extend: 10, soft-edge: 8). The multi-body refinement yielded mean resolutions of 3.7 Å for the DH and 5.2 Å for DDK I. Local resolution estimation of each map was then determined using the last iteration of split output of unfiltered half-maps as input in RELION. The multi-body maps were combined to generate a composite map using an atomic model (derived from MD-(ATPγS) state III), with the program combine_focused_maps in Phenix^69^.

The MD-(ATPγS) state III grouped particles were used for 3D auto-refinement, yielding a 6.5 Å mean resolution map (FSC = 0.143 cut-off) which featured the DH bound to two DDK molecules. The DDK density however appeared to suffer from conformational heterogeneity. The 3D auto-refined particles were subjected to 3D classification (*K* = 5, *T* = 4) without image alignment, and using a soft mask (derived from the 3D auto-refined map) encompassing both DDK subunits (extend: 3 pixel, soft-edge: 6 pixel). The 3D classes which displayed blurry DDK density on both sides of the DH were discarded and the classes that displayed strong DDK density on either side or both sides (containing 402,860 particles) were selected. The particles were subjected to another round of 3D classification (*K* = 5, *T* = 4) with image alignment and one class, containing 395,256 particles, which featured the most detailed structural features, was selected. The particles were then subjected to multiple rounds of 3D classification, some classifications were performed without image alignment and with a mask encompassing each DDK on either side of the DH (DDK I/II). The particles were then subjected to 3D auto-refinement, re-centered and re-extracted with a pixel size of 1.06 Å and a 360-pixel particle box size and then an additional round of 3D auto-refinement was performed. The particles were again subjected to 3D classification (*K* = 10, *T* = 4) without image alignment, and using a soft mask encompassing both DDK subunits (extend: 6 pixel, soft-edge: 8 pixel), followed by multiple rounds of 3D classification. The selected classes featured secondary structure elements for the DH and both DDK subunits, a total of 105,672 particles were used for 3D auto-refinement and two rounds of per-particle CTF refinement and Bayesian polishing and 3D auto-refinement. The 3D auto-refined particles were then subjected to several rounds of 3D classification without image alignment and 3D auto-refinement, with either a soft mask encompassing both DDK and local MCM2-7 subunits (extend: 12 pixel, soft-edge: 8 pixel) or a soft mask encompassing only the DDK core (extend: 12 pixel, soft-edge: 8 pixel). The masked classification significantly improved the DDK density in resulting maps and the complex featured C2 symmetry. The remaining 73,093 particles were subjected to 3D auto-refinement with C2 symmetry and C1 symmetry, yielding a 3.5 Å and 3.7 Å mean resolution map, respectively. There were no visual differences between the C2 and C1 map, the C1 auto-refined particles were then used for multi-body refinement. C2 symmetry was then exploited by performing C2 symmetry particle expansion mid-way through auto-refinement and the refinement was continued with local searches only. The auto-refined particles were subjected to 3D multi-body refinement and flexible analysis using three masked bodies (extend: 10, soft-edge: 8). The multi-body refinement yielded mean resolutions of 3.2 Å for the DH and 3.6 Å for both DDK I and II. Local resolution estimation of each map was then determined using the last iteration of split output of unfiltered half-maps as input in RELION. The multi-body maps were combined to generate a composite map using an atomic model (derived from MD-(ATPγS) state III), with the program combine_focused_maps in Phenix.

An overview of the cryo-EM image processing work-flow of the MD-(ATPγS) data is shown in Supplementary Fig. 3 and summarized in Supplementary Table 6.

MD-(ADP:BeF_3_)—The cryo-EM super-resolution multi-frame movies of the MD-(ADP:BeF_3_) complex were motion corrected, dose weighted, Fourier binned 2 × 2 (1.072 Å/pixel) and summed using MotionCor2. The micrographs were gain-corrected using a gain reference file and the frame alignment was performed using a B-factor of 150, averaging in groups of one frame and in patches (X, Y) of 5 × 5. CTF estimation was then performed on non-dose weighted summed micrographs using CTFFIND4. A sub-set of 966 micrographs were selected for initial small-scale processing of the data. A set of 600 particles were manually picked and used to generate reference-free 2D class averages for reference-based auto-picking. A total of 316,690 particles were automatically picked from 966 dose-weighted micrographs using RELION. The particles were Fourier binned 3 × 3 (3.216 Å/pixel), extracted with a 120-pixel particle box size and subjected to multiple rounds of 2D classification to separate out junk particles and improve the 2D classes. In the final 2D classification (*K* = 10, *T* = 3) round, seven classes were selected (containing 74,632 particles) and used as references for reference-based auto-picking of the complete data set. A total of 4,255,881 particles were automatically picked from 13,470 dose-weighted micrographs using RELION. The particles were Fourier binned 3 × 3 (3.216 Å/pixel) and extracted with a 120-pixel particle box size. The particles were subjected, firstly, to reference-free 2D classification (*K* = 50, *T* = 2) without CTF to remove contamination and large junk particles, followed by another round of 2D classification (*K* = 25, *T* = 2) with CTF. The 2D class averages selected (containing 1,935,820 particles) showed secondary structure features for the DH core and blurred regions that matched the expected size and shape of DDK. The particles were subjected to 3D classification (*K* = 12, *T* = 3) and the resulting classes were separated into two different groups based on visually determined conformational heterogeneity: state I group and swiveled state group.

The MD-(ADP:BeF_3_) state I group consisted of two sub-groups: group 1 DDK (213,410 particles) and group 2 DDK (565,019 particles) based on classes which featured density for either one DDK or two DDK bound to the DH, respectively. The sub-groups were initially separated but later combined. The group 1 DDK separated particles were subjected to 3D auto-refinement, followed by 3D classification (*K* = 10, *T* = 4) without image alignment, and using a soft mask (derived from the 3D auto-refined map) encompassing the one DDK subunit (extend: 10 pixel, soft-edge: 6 pixel). The particles were then subjected to 3D auto-refinement, and then the 3D auto-refined particles were used to re-center and re-extract the particles with a pixel size of 1.072 Å and a 360-pixel particle box size. The particles were subjected to 3D auto-refinement, yielding a 4.1 Å mean resolution map (FSC = 0.143 cut-off) that clearly featured the DH bound to one DDK subunit. The DDK density however appeared to still suffer from conformational heterogeneity. The particles were further subjected to multiple rounds of 3D classification (with and without image alignment and masking of DDK) and 3D auto-refinement. The remaining 29,929 particles were then combined with the processed group 2 DDK sub-group particles. The group 2 DDK separated particles were subjected to 3D auto-refinement, and then the 3D auto-refined particles were used to re-center and re-extract the particles with a pixel size of 1.072 Å and a 360-pixel particle box size. The particles were then subjected to multiple rounds of 3D classification and 3D refinement. In some 3D classifications, a loose soft mask around the DDK density (extend: 10, soft-edge: 6) was used and the classification was performed without alignment using 3D auto-refined particles. The classes that started to display detailed structural map features for DDK were selected. The final 3D auto-refinement (using 41,449 particles), prior to combining the particles, yielded a 4.8 Å mean resolution map (FSC = 0.143 cut-off) which featured the DH bound to two DDK molecules with detailed features around one DDK but poor density around the other DDK. It was not attempted to improve the other DDK density due to the cost of low particle number, instead, the particles were combined with the processed group 1 DDK sub-group particles. A combined total of 71,378 particles were subjected to 3D auto-refinement and the 3D auto-refined particles were then used for per-particle CTF refinement and Bayesian polishing and another round of 3D auto-refinement. The particles were yet again subjected to multiple rounds of 3D classification (with and without image alignment and masking of DDK) and 3D auto-refinement. The classes (containing 30,807 particles) which displayed improved detailed map features for DDK were selected and the particles were subjected to a final round of 3D auto-refinement (C1 symmetry), yielding a 4.5 Å mean resolution map (FSC = 0.143 cut-off). To further reduce the conformational heterogeneity of DDK, the map was segmented into two bodies, one encompassing the well-resolved DDK and the other the DH core. The auto-refined particles were subjected to 3D multi-body refinement and flexible analysis using two soft masked bodies (extend: 13, soft-edge: 8). The multi-body refinement yielded mean resolutions of 3.8 Å for the DH and 4.4 Å for DDK I. Local resolution estimation of each map was then determined using the last iteration of split output of unfiltered half-maps as input in RELION. The multi-body maps were combined to generate a composite map using an atomic model (derived from MD-(ADP:BeF_3_) state I group), with the program combine_focused_maps in Phenix.

The swiveled state group consisted of one class (containing 261,463 particles, 13.5% of input), the class was found to display a completely different conformation for DDK, where the density for DDK appeared to be at the ‘back’ of the DH forming a head-to-head configuration instead of facing opposing sides. The micrographs were filtered so that micrographs that displayed thorn rings at 5 Å or worse were discarded and then the 3D auto-refined particles were used to re-center and re-extract 231,320 particles with a pixel size of 1.072 Å and a 360-pixel particle box size. The unbinned particles were subjected to 3D auto-refinement, yielding a 4.4 Å mean resolution map (FSC = 0.143 cut-off) which featured the DH bound to two DDK molecules in a swiveled configuration MD-(ADP:BeF_3_) swiveled state). In this conformation, we observed the HBRCT domain of Dbf4 bound to Mcm2 on both hexamers, and density that matches the size expected for two DDK molecules facing each other. The density for the DDK core was too poor to formulate further conclusions other than relative position to the DH. It was not possible to improve the local density of DDK with further processing due to, ultimately, low particle number. Instead, the 3 × 3 binned particles prior to extraction were used to understand the heterogeneity of the data. The binned particles were subjected to 3D classification (*K* = 20, *T* = 4) and classes featuring diverse swiveled DDK conformations were selected. The classes were split into six distinct groups and the particles were either subjected to 3D auto-refinement or further subjected to another round of 3D classification (*K* = 3, *T* = 4) to improve the 3D map features and reduce the conformational heterogeneity and then subjected to 3D auto-refinement. The 3D auto-refinements (C1 symmetry) yielded a range between 11 and 20 Å mean resolution (FSC = 0.143 cut-off) for each different swiveled state (A-F) map. Local resolution estimation of each map was then determined using the last iteration of split output of unfiltered half-maps as input in RELION. The local resolution filtered maps allowed better map interpretation and manual docking of atomics models of the DH and DDK/Dbf4 HBRCT domain derived from MD-(ADP:BeF_3_) state I.

An overview of the cryo-EM image processing work-flow of the MD-(ADP:BeF_3_) data is shown in Supplementary Fig. 8 and summarized in Supplementary Table 7.

MD-(ATP)—The cryo-EM multi-frame movies of the MD-(ATP) complex were motion corrected, dose weighted and summed using MotionCor2. The frame alignment was performed using a B-factor of 150 and in patches (X, Y) of 5 × 5. CTF estimation was then performed on non-dose weighted summed micrographs using CTFFIND4. Micrographs were then inspected; crystalline ice contaminated micrographs were discarded and only micrographs which featured thorn rings at 5 Å or better were used for further processing. A set of 1500 particles were manually picked and used to generate reference-free 2D class averages for reference-based auto-picking. A total of 311,395 particles were automatically picked from 2,957 dose-weighted micrographs using RELION. The particles were Fourier binned 3 × 3 (3.255 Å/pixel) and extracted with a 120-pixel particle box size. The particles were subjected to 2D classification, first without CTF (*K* = 25, *T* = 2) to separate out junk particles and contaminants and then with CTF (*K* = 30, *T* = 2). The 2D class averages selected (containing 213,194 particles) showed secondary structure features for the DH core and blurred regions that matched the expected size and shape of DDK. The particles were then used to generate five ab initio models using RELION and the most featureful model was used for further processing. One round of 3D classification (*K* = 10, *T* = 4) was then performed and a class, containing 41,652 particles, that featured the DH (with DNA density in the center) bound to two DDK like subunits on the side, was selected. The particles were then subjected to 3D auto-refinement (C1 symmetry) and then re-centered and re-extracted with a pixel size of 1.085 Å and a 360-pixel particle box size. To capture only DH-DNA bound particles, the re-extracted particles were subjected to 3D auto-refinement and the resulting alignments were then used for 3D classification (*K* = 2, *T* = 4) without image alignment, and using a soft mask (derived from the 3D auto-refined map) encompassing the DNA density (extend:13 pixel, soft-edge: 6 pixel). The DNA density was clearly present in only one class, out of the two classes, which contained 28,527 particles (68% of input). The DNA containing particles were selected and used for 3D auto-refinement, yielding a 7.2 Å mean resolution map (FSC = 0.143 cut-off). The map featured two DDK molecules bound to the DH at the MCM2-7 N-N terminal interface, however, the density for DDK was asymmetric with one DDK featuring stronger density than the other. In addition, the density of each DDK seemed to suffer from conformational heterogeneity. To obtain a more homogeneous conformational state, the 3D auto-refined particles were further subjected to 3D classification (*K* = 5, *T* = 10) without image alignment, and using a soft mask surrounding the DDK subunit with the strongest density (extend: 13 pixel, soft-edge: 6 pixel). The density for DDK improved significantly in one of the classes, which featured a more detailed bi-lobal DDK structure and contained 5,528 particles (19% of input). The difference between the classes was easily distinguished from the 2D cross-sections of the 3D models. It was not attempted to improve the second DDK density due to the cost of low particle number. The particles were subjected to 3D auto-refinement, per-particle CTF refinement, Bayesian polishing and another round of 3D auto-refinement (C1 symmetry), yielding a 11.4 Å mean resolution map (FSC = 0.143 cut-off) with a well-defined density for one of the DDK molecules. To further reduce the conformational heterogeneity of DDK, the map was segmented into three bodies, separating each DDK and the DH core. The auto-refined particles were subjected to 3D multi-body refinement and flexible analysis using three soft masked bodies (extend: 15, soft-edge: 8). The multi-body refinement yielded mean resolutions of 8.3 Å, 11.0 Å and 9.8 Å for DH core, DDK I (defined shape) and DDK II, respectively. The map of the DDK II, although reporting higher resolution, was difficult to interpret. Local resolution estimation of each map was then determined using the last iteration of split output of unfiltered half-maps as input in RELION. An overview of the cryo-EM image processing work-flow of MD-(ATP) is shown in Supplementary Fig. 9 and summarized in Supplementary Table 8. The MD-(ATP) map was analysed and interpreted by rigid-body fitting of the DH-DNA structure from PDB: 5BK4 and the DDK structure from the MD-(ATPγS) (Fig. 6). The nucleotide and associated ions within the DDK active site were removed for the purpose of fitting.

### Atomic model building

The MD-(ATPγS) state III atomic model was built manually in Coot^70^, using as starting models: the re-refined budding yeast MCM2-7 single hexamer (PDB: 6EYC), a SWISS model^71^ predicated structure of DDK based on the crystal structure of human Cdc7-Dbf4 (PDB: 6YA7) and the crystal structure of the budding yeast Dbf4 HBRCT domain (PDB: 3QBZ). The starting models were first docked as rigid bodies into the composite MD-(ATPγS) state III map using UCSF Chimera^72^. The composite MD-(ATPγS) state III map featured the highest resolution MCM2-7 DH to date and so the DH was extensively re-built, and additional short regions not resolved before were modelled (see also Supplementary Fig. 4). The DDK subunits were extensively modelled and building of new regions was aided by secondary structure predication using PSIPRED^73^. In order to help with map interpretation and modelling, the map was auto-sharpened using the auto_sharpen program in Phenix. The Cdc7 aa424-475 segment was modelled using a SWISS model predicted structure, based on the crystal structure of human Serine/threonine-protein kinase 4 (PDB: 6YAT), as a starting model. The MD-(ATPγS) atomic model was treated as a C2 symmetric structure. The model was subjected to real-space refinement against a composite EM map (generated from multi-body auto-refined maps) using Phenix. The refinement was performed using global minimization with secondary structure restraints and NCS constraints. The MD-(ADP:BeF_3_) state I atomic model was built in a similar as that mentioned above, using the MD-(ATPγS) state III atomic model as a starting model. The MD-(ADP:BeF_3_) state I atomic model was treated as a C1 symmetric structure. There were several significant changes to the DH structure, particularly at the MCM2-7 zinc finger domains, and little to no difference to the overall DDK structure. For the modelled ADP:BeF_3_ molecules, the distance between the Be and O3B of ADP was restrained 1.6 Å and the distance between Mg^2+^ and F1 of BeF_3_ was restrained to 2.0 Å. The structure validation statistics for the MD-(ATPγS) state III atomic model and the MD-(ADP:BeF_3_) state I atomic model are shown in Supplementary Table 9. Molprobity^74^ was used for structure validation.

### Mass spectrometry sample processing

Protein samples (1 µg/sample) were processed using an in-solution digestion procedure. Briefly, samples were sequentially reduced and alkylated at room temperature and in the dark, using final concentrations of 10 mM dithiothreitol (DTT) and 50 mM 2-chloroacetamide (2-CAM), respectively. 250 ng of trypsin (Promega, V5280) was added to achieve an approximate 1:4 protease to protein ratio and samples were incubated at 37 °C overnight. The digestion was stopped by acidification with 1% trifluoroacetic acid (TFA) to a final concentration of 0.1% and protein digests were desalted using Glygen C18 spin tips (Glygen Corp, TT2C18.96). Tryptic peptides were eluted with 60% acetonitrile, 0.1% formic acid (FA) and eluents dried by vacuum centrifugation.

### Liquid chromatography-tandem mass spectrometry (LC-MS/MS) analysis

Dried tryptic digests were redissolved in 0.1% TFA by shaking (1200 rpm, 30 min) at room temperature, then sonicated on an ultrasonic water bath (5 min, degas setting), followed by centrifugation (13,000 rpm, 5 °C, 10 min). LC-MS/MS analysis was performed using an Ultimate 3000 RSLC nano liquid chromatography system (Thermo Scientific) coupled to a Q-Exactive HFX mass spectrometer (Thermo Scientific) via an EASY spray source (Thermo Scientific). Protein digests were injected and loaded onto a trap column (Acclaim PepMap 100 C18, 100 μm × 2 cm) for desalting and concentration at a flow rate of 8 μL/min (loading pump buffer: 2% acetonitrile, 0.1% TFA). Final on-column digest amount was 400 ng per injection. Peptides were then eluted on-line to an analytical column (Acclaim Pepmap RSLC C18, 75 μm × 50 cm) at a flow rate of 250 nl/min. Peptides were separated using a 90 min gradient, 1–22% of buffer A for 70 min followed by 22–42% buffer B for another 20 min (buffer A: 5% dimethylsulfoxide (DMSO), 0.1% FA in water; buffer B: 75% acetonitrile, 20% water, 5% DMSO, 0.1% FA) and subsequent column conditioning and equilibration. Eluted peptides were analysed by the mass spectrometer operating in positive polarity using a data-dependent acquisition mode. Ions for fragmentation were determined from an initial MS1 survey scan at 120,000 resolution, followed by HCD (Higher Energy Collision Induced Dissociation) fragmentation of the top 20 most abundant ions at 15,000 MS2 resolution. MS1 and MS2 scan AGC targets were set to 3e6 and 5e4, allowing maximum ion injection times of 25 ms and 100 ms, respectively. A survey scan *m/z* range of 350–1750 was used, normalised collision energy set to 27%, charge state exclusion enabled with unassigned and +1 charge states rejected and a minimal AGC target of 8e3. Dynamic exclusion was set to 50 s.

### Mass spectrometry data processing

Data were processed using the MaxQuant^13^ software platform (v1.6.10.43), with database searches carried out by the in-built Andromeda search engine against a Swissprot *S. cerevisiae* database (version 20200924, number of entries: 7905). A reverse decoy search approach was used at a 1% false discovery rate (FDR) for both peptide spectrum matches and protein groups. Search parameters included: maximum missed cleavages set to 2, fixed modification of cysteine carbamidomethylation and variable modifications of methionine oxidation, protein N-terminal acetylation, Carbamidomethyl-Thio-Phospho (STY) and Thio-Phospho (STY). Label-free quantification was enabled with an LFQ minimum ratio count of 2. ‘Match between runs’ function was enabled. Additional analysis was carried out using the Perseus (v1.6.15.0) software platform^75^. Phosphorylation sites identified were initially filtered to remove potential contaminants and hits to the reverse decoy database. Volcano plot analysis was performed with a pre-filter of intensities reported per experimental group (3 intensities per group). Two-sample *t*-test was carried out with multiple testing correction implemented at an FDR of 5%. Fold change was also considered in determining volcano plot significance thresholds (S0 parameter = 0.1). Hierarchical Clustering Analysis coupled to heat-map was used to visualise significantly changed sites between sample groups.

### Cdc7 and Dbf4 transition probability scores

Deep-learning transformer architectures have previously been trained on large protein sequence data sets^76,77^. These neural networks leverage the attention mechanism to extract evolutionary, functional, and structural information from sequence data alone. During training, a certain percentage of the sequence is masked, and the model must predict which amino acid belongs to each of the masked positions. The model gathers a knowledge base of protein language in terms of grammar and semantics of the data set on which it was trained on. The learned representations show significant improvement with increased data set size and model capacity. The use of a large pre-trained language model can aid in determining which amino acid residue(s) might be essential for protein function or stability. Here, we inferred the likelihood of a mutation at a given position using the evolutionary landscape of the original protein sequence. We obtained the transition probability scores for each amino acid residue at each position by applying a softmax function to the logit output of the transformer. The language model used for predicting the transition probabilities is a 650M-parameter transformer trained across 86 billion amino acids from 250 million sequences^76^.

### Preparation of atomic models for molecular dynamics simulation

The MD-(ATPγS) state III cryo-EM derived atomic model was used to generate starting initial models for molecular dynamics simulations. The MD-(ATPγS) state III atomic model was broken down into a more manageable structure featuring: Cdc7, Dbf4 (without aa111–220, harbouring the HBRCT domain), Mcm4 and Mcm6. The nucleotides and ions associated with these subunits were also included. The structure (M4-M6-DDK) still retained all the relevant core DDK interactions with the MCM2-7 DH. The core of DDK, in the Mcm4 targeted DDK conformation, only forms contacts with Mcm4 and Mcm6 on the same MCM2-7 hexamer. The HBRCT domain of Dbf4, which makes contacts with Mcm2 on the opposing MCM2-7 hexamer, is not part of the core of DDK and only serves as an anchor to locally recruit DDK to the DH. The M4-M6-DDK atomic model was used to reconstruct the flexible unresolved tail of Mcm4 (aa134-176). This stretch of residues was chosen since it was previously shown that the inhibitory activity of Mcm4 resides within aa74–174 and that aa146–174 are sufficient for exerting the inhibitory effect^24^. The tail was reconstructed using the Rosetta macromolecular modelling platform^58^, with a substantial part of the protocol implemented in PyRosetta^78^. Throughout the protocol, we have used the standard dual space energy function of Rosetta^79^, permitting for non-ideal protein geometry. The molecular dynamics simulation input atomic model was subjected to the robotics-based kinematic loop closure algorithm^80,81^, which allowed the bridging of extensive gaps in the structure, thus reducing the subsequent number of restraints necessary. The model was then subjected to a flexible loop rebuilding protocol, closely based on the FloppyTail subroutine^82^ of Rosetta, but implemented internally. The protocol progressively builds the missing chain of Mcm4, starting from the existing structure, by extending the structure in the N-terminal direction, and performing local conformational sampling. The sampling is directed at the newly built residue, the succeeding four residues, as well as their neighbourhood (which includes all the atoms of the residues for which at least one heavy atom is within 8 Å of the sampling target). This step interweaves sidechain packing with gradient-based minimization, and (optionally) small, local, torsion-space perturbations to the nascent structure, similar to Conway et al.^83^, which leads to locally optimal structures. This protocol is prone to be trapped in local energy minima, and proposing dead-end solutions which cannot feasibly result in a non-frustrated structure. These solutions have been automatically eliminated. Due to stochasticity of the method, we repeated the protocol over 1000 times, with slightly varying hyperparameters (such as the exhaustiveness of minimization, inclusion of local perturbations, variations in the scoring function values). The resulting models were then sorted with regard to the quality of the reconstructed tail, as measured by the sum of the scores of the new residues estimated by Rosetta’s all-atom energy function^79^. The lowest-energy reconstructed structures were manually visually inspected and three out of several hundred decoy models with different Mcm4 tail trajectories were selected. The models were chosen based on the criteria that the Mcm4 tail had to be positioned within close proximity to the DDK active site and that the tail would make little to no contact with core regions of Mcm4 and Mcm6. The selected extended Mcm4 N-terminal tail M4-M6-DDK atomic structures (M4(N)-M6-DDK) were then compared to ensure that the structures closely matched that of the cryo-EM resolved starting model, particularly at positions featuring nucleotides and ions. The atomic model.pdb file for each of the three structures were then altered so that each amino acid residue corresponded to non-overlapping residue numbers with a 3-residue gap inserted between each chain and any chain-breaks present within individual protein subunits were treated as separate chains. All ATPγS molecules within the structure were replaced with ATP for the purposes of the simulation.

### Molecular dynamics simulation

Molecular dynamics simulations were performed with GROMACS 2021.2^84^. Three separate simulations were performed, one for each selected initial model. The M4(N)-M6-DDK atomic models featuring: Mcm4, Mcm6, and DDK, along with three ATP molecules, four zinc ions, and three magnesium ions were used as initial models. Each system was parameterized with the CHARMM36-Jul2020 force field^85^. The initial conformation was placed in a dodecahedral box large enough to contain the complex with SPC/E water-occupied 1.0 nm distance from the box boundaries on all sides. Sodium (Na^+^) and chlorine (Cl^-^) ions were used to neutralize the system. Six positive counterions were used to replace an equal number of water molecules to produce a neutral simulation box, ready for minimization. The starting structure was subjected to a minimization protocol for a maximum of 50,000 steps or until the maximum force of the system was reduced to 1000.0 kJ/mol/nm, using the steepest descent method. This was followed by two sequential equilibration steps with position restraints on the protein atoms. First, an isothermal-isochoric equilibration was done for 100 ps which was followed by an isothermal-isobaric equilibration for 1000 ps. The zinc ions were positionally restrained throughout. Production simulations were performed at constant temperature (300 K) using Langevin dynamics. The pressure coupling was done by employing a Parrinello-Rahman barostat using 1 bar as reference pressure and a time constant of 2.0 ps with the compressibility of 4.5e−5 bar using an isotropic scaling scheme. The Particle Mesh Ewald algorithm was used to treat long-range electrostatics with a spline order of 4, a grid spacing of 0.12 nm, and both the direct space cut-off and Van der Waals cut-off set to 1.0 nm. The leap-frog integrator with a friction constant of 1.0 ps and a 2.0 fs time step for numerical integration of the equations of motion (integration time step) was used. Bonds involving hydrogen atoms were constrained by means of the LINCS algorithm, with a bond restraining order of 4. Coordinates were saved every 10.0 ps and the resulting simulations were ~400 ns each.

### Reporting summary

Further information on research design is available in the Nature Research Reporting Summary linked to this article.

## Supplementary information

Supplementary Information

Description of Additional Supplementary Files

Supplementary Movie 1

Supplementary Movie 2

Supplementary Movie 3

Supplementary Movie 4

Supplementary Movie 5

Reporting Summary

## Data Availability

The data that support this study are available from the corresponding author upon reasonable request. The EM maps and associated atomic models of MD-(ATPγS) state III and MD-(ADP:BeF3) state I reported in this study have been deposited to the Electron Microscopy Data Bank (EMDB) and the RCSB Protein Data Bank (PDB) under accession numbers EMD-13619 and 7PT6 [10.2210/pdb7PT6/pdb], and EMD-13620 and 7PT7 [10.2210/pdb7PT7/pdb], respectively. The EM maps of MD-(ATPγS) state I-II and additional maps of state I-III have been deposited under the accession numbers: EMD-13644, EMD-13640, EMD-13631, EMD-13635, EMD-13629, EMD-13624, EMD-13621, EMD-13622, EMD-13623. The EM maps of MD-(ADP:BeF3) swiveled states and additional maps of state I, have been deposited under the accession numbers: EMD-13647, EMD-13645, EMD-13646, EMD-13648, EMD-13649, EMD-13650, EMD-13651, EMD-13652, EMD-13653, EMD-13655. The EM maps of MD-(ATP) have been deposited under the accession numbers: EMD-13656, EMD-13657, EMD-13658, EMD-13659. Proteomics data have been deposited to PRIDE under the accession number PXD031315.

## Source data

Source Data
