# Supplementary material for: The structural basis of Cdc7-Dbf4 kinase dependent targeting and phosphorylation of the MCM2-7 double hexamer
Source: Nat Commun. 2022 May 25;13:2915. doi: 10.1038/s41467-022-30576-1 (PMC9133112; doi:10.1038/s41467-022-30576-1)
Supplement: Supplementary file 1 — Supplementary Information [file 41467_2022_30576_MOESM1_ESM.pdf]

Supplementary Information

The structural basis of Cdc7-Dbf4 kinase dependent targeting and phosphorylation of the MCM2-7 double hexamer

Almutasem Saleh[1,*] , Yasunori Noguchi[1,*], Ricardo Aramayo[1], Marina E. Ivanova[1], Kathryn M. Stevens[1,2], Alex Montoya[3], Sunidhi S[4], Nicolas Lopez Carranza[4], Marcin J. Skwark[4], and Christian Speck[1,2]

[1] DNA Replication Group, Institute of Clinical Sciences, Faculty of Medicine, Imperial College London, London W12 0NN, UK
[2] MRC London Institute of Medical Sciences (LMS), W12 0NN London, United Kingdom.
[3] Proteomics and Metabolomics Facility, MRC London Institute of Medical Sciences, Imperial College London, Hammersmith Hospital Campus, Du Cane Road, London, W12 0NN, UK
[4] InstaDeep Ltd, 2 Eastbourne Terrace, London, W2 6LG, UK

* These authors contributed equally
Correspondence: chris.speck@imperial.ac.uk

This Supplementary Information contains: 13 Supplementary Figures, 9 Supplementary Tables and 5 Supplementary Movies.

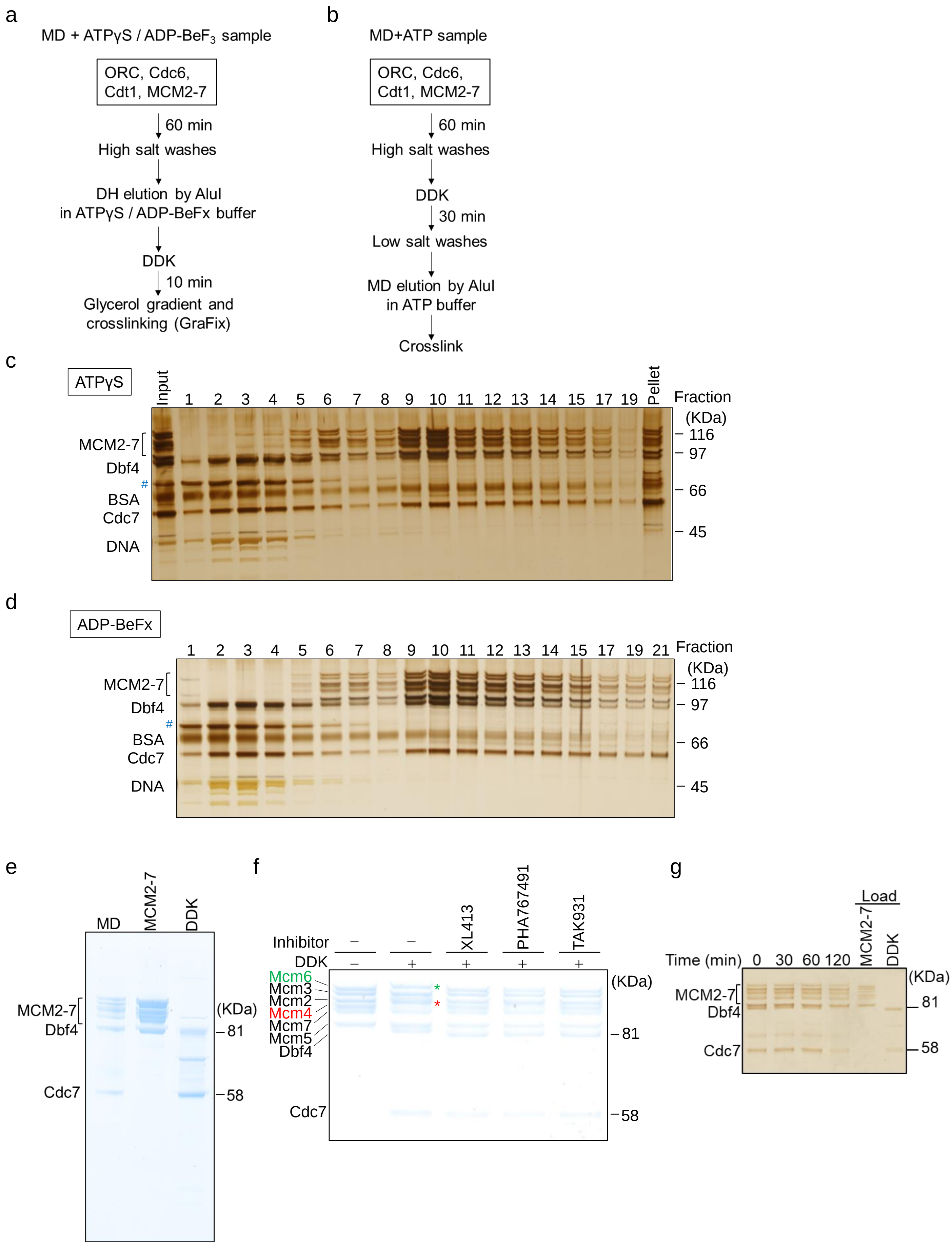
a
MD + ATPγS / ADP-BeF3 sample
ORC, Cdc6, Cdt1, MCM2-7
60 min
High salt washes
DH elution by AluI in ATPγS / ADP-BeFx buffer
DDK
10 min
Glycerol gradient and crosslinking (GraFix)
b
MD+ATP sample
ORC, Cdc6, Cdt1, MCM2-7
60 min
High salt washes
DDK
30 min
Low salt washes
MD elution by AluI in ATP buffer
Crosslink
c
ATPγS
Input 1 2 3 4 5 6 7 8 9 10 11 12 13 14 15 17 19 Pellet
Fraction
(KDa)
116
97
66
45
MCM2-7
Dbf4
#
BSA
Cdc7
DNA
d
ADP-BeFx
1 2 3 4 5 6 7 8 9 10 11 12 13 14 15 17 19 21
Fraction
(KDa)
116
97
66
45
MCM2-7
Dbf4
#
BSA
Cdc7
DNA
e
MD
MCM2-7
DDK
MCM2-7
Dbf4
Cdc7
(KDa)
81
58
f
Inhibitor – – XL413 PHA767491 TAK931
DDK – + + + +
Mcm6
Mcm3
Mcm2
Mcm4
Mcm7
Mcm5
Dbf4
Cdc7
*
*
(KDa)
81
58
g
Load
Time (min) 0 30 60 120 MCM2-7 DDK
MCM2-7
Dbf4
Cdc7
(KDa)
81
58

Supplementary Figure 1. *In vitro* reconstitution of the MD complex and kinase activity inhibition. a-b. Reaction scheme of the cryo-EM sample preparation method used for **(a)** MD-(ATPγS) and MD-(ADP:BeF_3) and **(b)** MD-ATP. **c-d.** SDS-PAGE of glycerol gradient fractions of the MD complex in the presence of **(c)** ATPγS and **(d)** ADP:BeF_3. The fractions marked with a red dotted square correspond to the same pooled fractions in the crosslinked gradient (GraFix) used for cryo-EM sample preparation. The band labelled # is a contamination originating from the DDK purification (heat shock protein Ssa1). **e.** SDS-PAGE of MD-(ATP) complex sample prior to crosslinking. **f.** SDS-PAGE of kinase activity inhibition assay. The MCM2-7 DH bound to DNA-beads was incubated in pre-RC buffer with 100 µM ATP and 300 nM DDK in the presence of 100 µM Cdc7 inhibitor. The DNA-beads were washed and the MD complex was eluted and then analysed by SDS-PAGE and stained by Coomassie blue. **g.** MD complex time course stability assay. The MD complex was reconstituted on DNA-beads, left at room temperature, washed with pre-RC buffer, eluted at different time points and then analysed by SDS-PAGE and silver staining. In each experiment similar results were obtained in at least two independent experiments.

a

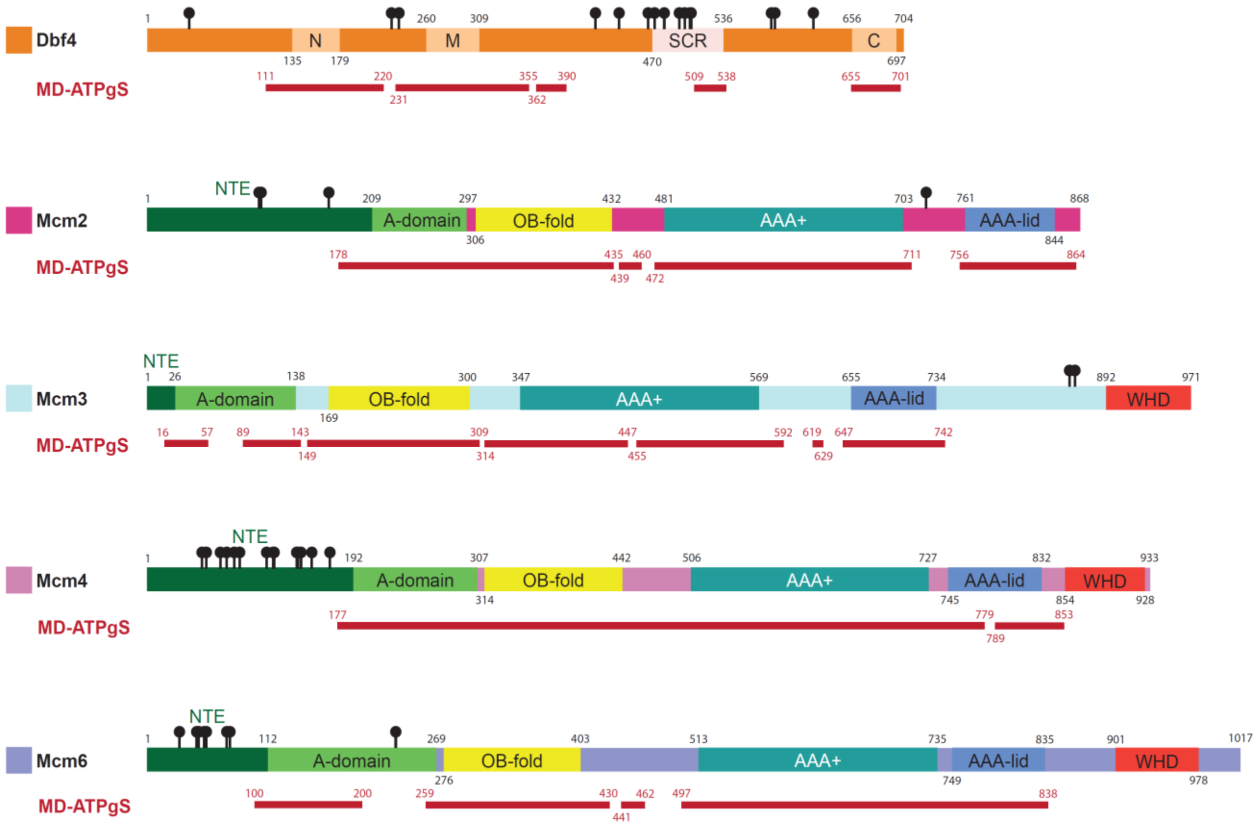

b

Mcm2 101 QQELSLSERR RIDAQLNERD RLLRNVAYID DEDEEQEGAA QLDEMGLPVQ 150
RRRRRRQYED LENSDDDLLS DMDIDPLREE 180

Mcm4 1 MSQQSSSPTK EDNNSSSPVV PNPDSVPPQL SSPALFYSSS SSQGDIYGRN 50
NSQNLSQGEG NIRAAIGSSP LNFPSSSQRQ NSDVFQSQGR QGRIRSSASA 100
SGRSRYHSDL RSDRALPTSS SSLGRNGQNR VHMRRNDIHT SDLSSPRRIV 150
DFDTRSGVNT LDTSSSSAPP SEASEPLRII 180

Mcm6 1 MSSPFPADTP SSNRPSNSSP PPSSIGAGFG SSSGLDSQIG SRLHFPSSSQ 50
PHVSNSQTGP FVNDSTQFSS QRLQTDGSAT 80

Supplementary Figure 2. DDK-dependent phosphorylation sites of MCM2-7 and DDK.
a. Schematic diagram to illustrate the domain organization and features of components of the MCM2-7-DDK (MD) complex from budding yeast (Sc, *Saccharomyces cerevisiae*). The structurally resolved regions of each protein subunit from data acquired in this study are indicated by red colored bars. The DDK-dependent thio-phosphorylation sites determined by phosphoproteomic are indicated by black pins. **b.** Sequence of the N-terminal region of Mcm2, Mcm4 and Mcm6. The DDK-dependent thio-phosphorylation sites within the MD complex are highlighted in red. Refer to Supplementary. Fig 4 for domain abbreviations.

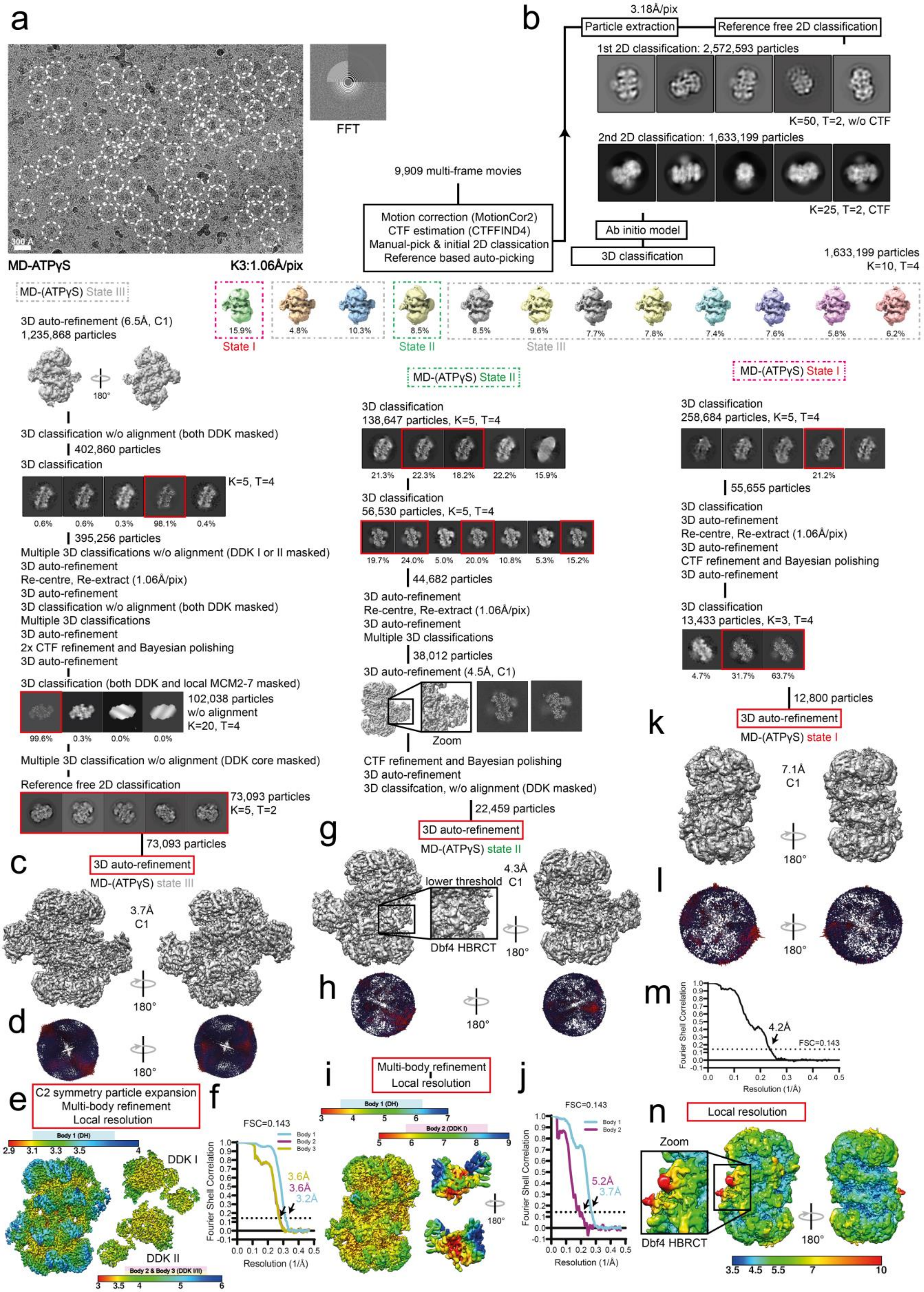

a
300 Å
FFT
MD-ATPγS
K3:1.06Å/pix
b
3.18Å/pix
Particle extraction
Reference free 2D classification
1st 2D classification: 2,572,593 particles
K=50, T=2, w/o CTF
2nd 2D classification: 1,633,199 particles
K=25, T=2, CTF
9,909 multi-frame movies
Motion correction (MotionCor2)
CTF estimation (CTFFIND4)
Manual-pick & initial 2D classication
Reference based auto-picking
Ab initio model
3D classification
1,633,199 particles
K=10, T=4
15.9%
4.8%
10.3%
8.5%
8.5%
9.6%
7.7%
7.8%
7.4%
7.6%
5.8%
6.2%
State I
State II
State III
MD-(ATPγS) State III
3D auto-refinement (6.5Å, C1)
1,235,868 particles
180°
3D classification w/o alignment (both DDK masked)
402,860 particles
3D classification
K=5, T=4
0.6%
0.6%
0.3%
98.1%
0.4%
395,256 particles
Multiple 3D classifications w/o alignment (DDK I or II masked)
3D auto-refinement
Re-centre, Re-extract (1.06Å/pix)
3D auto-refinement
3D classification w/o alignment (both DDK masked)
Multiple 3D classifications
3D auto-refinement
2x CTF refinement and Bayesian polishing
3D auto-refinement
3D classification (both DDK and local MCM2-7 masked)
102,038 particles
w/o alignment
K=20, T=4
99.6%
0.3%
0.0%
0.0%
Multiple 3D classification w/o alignment (DDK core masked)
Reference free 2D classification
73,093 particles
K=5, T=2
73,093 particles
c
3D auto-refinement
MD-(ATPγS) state III
3.7Å
C1
180°
d
180°
e
C2 symmetry particle expansion
Multi-body refinement
Local resolution
Body 1 (DH)
2.9
3.1
3.3
3.5
4
DDK I
DDK II
Body 2 & Body 3 (DDK I/II)
3
3.5
4
5
6
f
FSC=0.143
Body 1
Body 2
Body 3
3.6Å
3.6Å
3.2Å
Fourier Shell Correlation
Resolution (1/Å)
MD-(ATPγS) State II
3D classification
138,647 particles, K=5, T=4
21.3%
22.3%
18.2%
22.2%
15.9%
3D classification
56,530 particles, K=5, T=4
19.7%
24.0%
5.0%
20.0%
10.8%
5.3%
15.2%
44,682 particles
3D auto-refinement
Re-centre, Re-extract (1.06Å/pix)
3D auto-refinement
Multiple 3D classifications
38,012 particles
3D auto-refinement (4.5Å, C1)
Zoom
CTF refinement and Bayesian polishing
3D auto-refinement
3D classifcation, w/o alignment (DDK masked)
22,459 particles
g
3D auto-refinement
MD-(ATPγS) state II
4.3Å
C1
lower threshold
Dbf4 HBRCT
180°
h
180°
i
Multi-body refinement
Local resolution
Body 1 (DH)
3
4
5
6
7
Body 2 (DDK I)
5
6
7
8
9
180°
j
FSC=0.143
Body 1
Body 2
5.2Å
3.7Å
Fourier Shell Correlation
Resolution (1/Å)
MD-(ATPγS) State I
3D classification
258,684 particles, K=5, T=4
21.2%
55,655 particles
3D classification
3D auto-refinement
Re-centre, Re-extract (1.06Å/pix)
3D auto-refinement
CTF refinement and Bayesian polishing
3D auto-refinement
3D classification
13,433 particles, K=3, T=4
4.7%
31.7%
63.7%
12,800 particles
k
3D auto-refinement
MD-(ATPγS) state I
7.1Å
C1
180°
l
180°
m
4.2Å
FSC=0.143
Fourier Shell Correlation
Resolution (1/Å)
n
Local resolution
Zoom
Dbf4 HBRCT
180°
3.5
4.5
5.5
7
10

Supplementary Figure 3. Cryo-EM image processing work-flow and 3D reconstruction of the MD-(ATPγS) complex.

a. Representative cryo-EM micrograph and corresponding fast Fourier transform (FFT). The micrograph features monodisperse particles of MD-(ATPγS) which are indicated by dotted circles. Scale bar is also shown. **b.** Image data processing work-flow. **c.** 3D auto-refined map of MD-(ATPγS) state III at 3.7Å mean resolution. **d.** Euler angle particle distribution of map shown in **(c)**. **e.** Multibody-body auto-refined MD-(ATPγS) state III maps of the DH and DDK. The MD-(ATPγS) state III map was split into three separate bodies for multi-body refinement and the resulting local resolution estimation of the three different bodies is shown. **f.** Gold-standard Fourier Shell Correlation plot of masked multi-body MD-(ATPγS) state III maps. **g.** 3D auto-refined map of MD-(ATPγS) state II at 4.3Å mean resolution. **h.** Euler angle particle distribution of map shown in **(h)**. **i.** Multibody-body auto-refined MD-(ATPγS) state II maps of the DH and DDK. The MD-(ATPγS) state III map was split into two separate bodies for multi-body refinement and the resulting local resolution estimation of the two different bodies is shown. **j.** Gold-standard Fourier Shell Correlation plot of masked multi-body MD-(ATPγS) state II maps. **k.** 3D auto-refined map of MD-(ATPγS) state I at 7.1Å mean resolution. **l.** Euler angle particle distribution of map shown in (**k**). **m.** Gold-standard Fourier Shell Correlation plot of post-processed MD-(ATPγS) state I map with a loose soft mask and a B-factor of 0 applied. **n.** Local resolution estimation of the MD-(ATPγS) state I map. The Euler angle particle distribution plots shown were generated using the RELION bild file output (blue bar represents the presence of a particle 2D view at a defined angle and red bar indicates a relatively high number of particles with that angle view). The front and back views of the bodies featuring mainly DDK regions are shown. Resolution values shown are in angstroms.

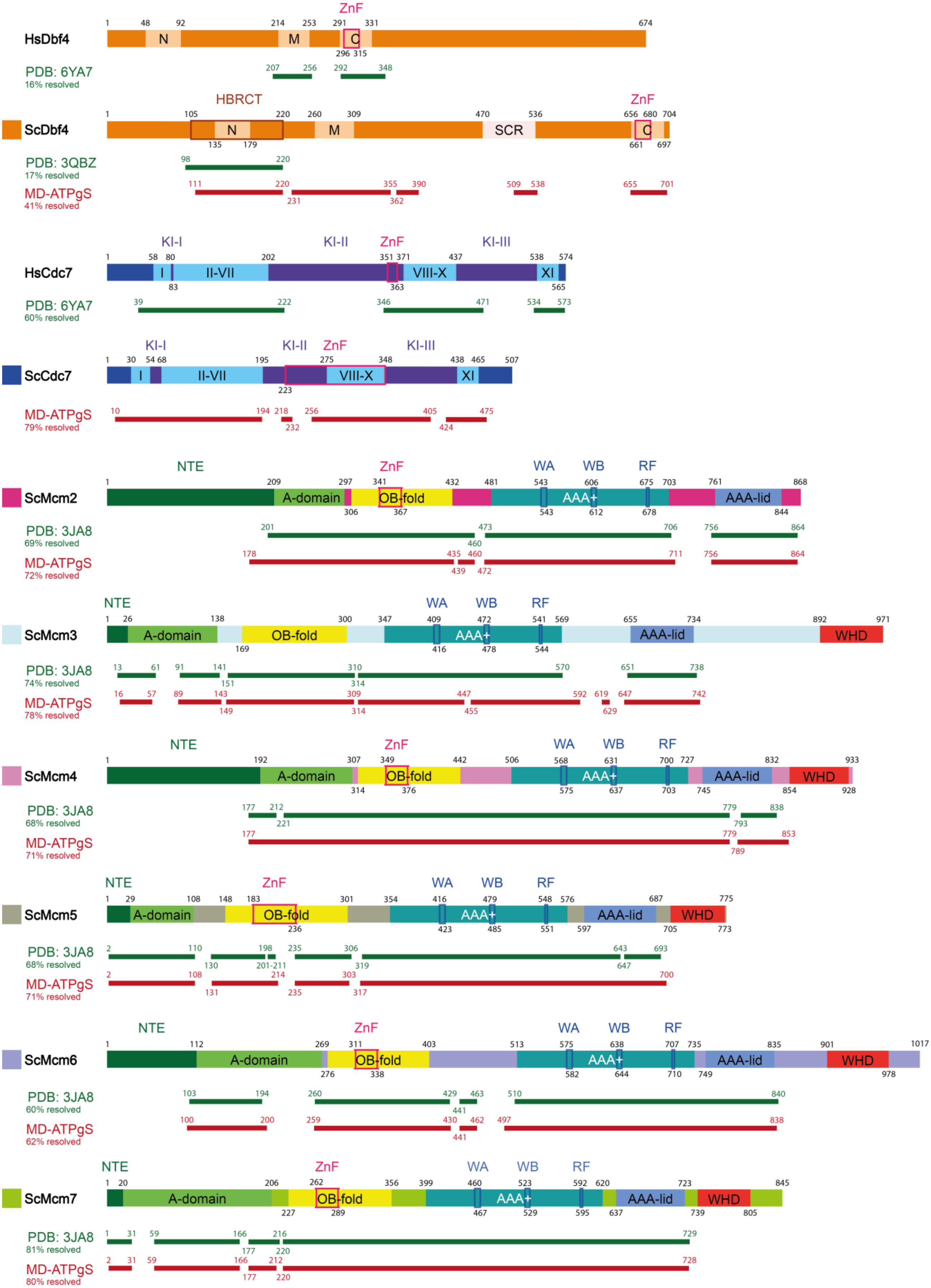

HsDbf4
1 48 92 214 253 291 331 674
N
M
ZnF
C
296 315
PDB: 6YA7
16% resolved
207 256 292 348
ScDbf4
HBRCT
1 105 220 260 309 470 536 656 680 704
N
M
SCR
ZnF
C
135 179 661 697
PDB: 3QBZ
17% resolved
98 220
MD-ATPgS
41% resolved
111 220 355 390 509 538 655 701
231 362
HsCdc7
KI-I KI-II ZnF KI-III
1 58 80 202 351 371 437 538 574
I II-VII VIII-X XI
83 363 565
PDB: 6YA7
60% resolved
39 222 346 471 534 573
ScCdc7
KI-I KI-II ZnF KI-III
1 30 54 68 195 275 348 438 465 507
I II-VII VIII-X XI
223
MD-ATPgS
79% resolved
10 194 218 256 405 475
232 424
ScMcm2
NTE ZnF WA WB RF
1 209 297 341 432 481 543 606 675 703 761 868
A-domain OB-fold AAA+ AAA-lid
306 367 543 612 678 844
PDB: 3JA8
69% resolved
201 473 706 756 864
460
MD-ATPgS
72% resolved
178 435 460 711 756 864
439 472
ScMcm3
NTE WA WB RF
1 26 138 300 347 409 472 541 569 655 734 892 971
A-domain OB-fold AAA+ AAA-lid WHD
169 416 478 544
PDB: 3JA8
74% resolved
13 61 91 141 310 570 651 738
151 314
MD-ATPgS
78% resolved
16 57 89 143 309 447 592 619 647 742
149 314 455 629
ScMcm4
NTE ZnF WA WB RF
1 192 307 349 442 506 568 631 700 727 832 933
A-domain OB-fold AAA+ AAA-lid WHD
314 376 575 637 703 745 854 928
PDB: 3JA8
68% resolved
177 212 779 838
221 793
MD-ATPgS
71% resolved
177 779 853
789
ScMcm5
NTE ZnF WA WB RF
1 29 108 148 183 301 354 416 479 548 576 687 775
A-domain OB-fold AAA+ AAA-lid WHD
236 423 485 551 597 705 773
PDB: 3JA8
68% resolved
2 110 198 235 306 643 693
130 201-211 319 647
MD-ATPgS
71% resolved
2 108 214 303 700
131 235 317
ScMcm6
NTE ZnF WA WB RF
1 112 269 311 403 513 575 638 707 735 835 901 1017
A-domain OB-fold AAA+ AAA-lid WHD
276 338 582 644 710 749 978
PDB: 3JA8
60% resolved
103 194 260 429 463 510 840
441
MD-ATPgS
62% resolved
100 200 259 430 462 497 838
441
ScMcm7
NTE ZnF WA WB RF
1 20 206 262 356 399 460 523 592 620 723 845
A-domain OB-fold AAA+ AAA-lid WHD
227 289 467 529 595 637 739 805
PDB: 3JA8
81% resolved
1 31 59 166 216 729
177 220
MD-ATPgS
80% resolved
2 31 59 166 212 728
177 220

Supplementary Figure 4. 2D domain organization of MCM2-7, Cdc7 and Dbf4.

Schematic diagram to illustrate the domain organization and features of components of the MCM2-7-DDK (MD) complex from budding yeast (Sc, *Saccharomyces cerevisiae*). (Hs, *Homo sapiens*) homolog proteins of Cdc7 and Dbf4 are shown for comparison. The structurally resolved regions of each protein subunit from previously available data (green colored bar) verses data acquired in this study (red colored bar) are indicated. The MCM2-7 proteins harbor the following domains: N-terminal extension (NTE) domain, A-domain, oligonucleotide binding fold (OB-fold) domain, AAA+ ATPase (AAA+) domain and winged helix (WHD) domain. The MCM OB domain has additional distinct motifs such as: Walker A (WA) motif, Walker B (WB) motif and arginine finger (RF) motif. The domain boundary of residues involved in the coordination of zinc ions is indicated by the zinc finger (ZF) domain. The Cdc7 kinase is a highly conserved protein among all eukaryotes. The highly conserved catalytic kinase domains are indicated by the roman numerals I-IV and the less conserved kinase insert domains as (KI). The Dbf4 regulatory protein features three unique motifs and a modified domain: motif-N, motif-M and motif-C and α-helix-BRCA1 C-terminal (HBRCT) domain. The substrate coordinating region (SCR) of Dbf4 is currently only attributed to yeast species.

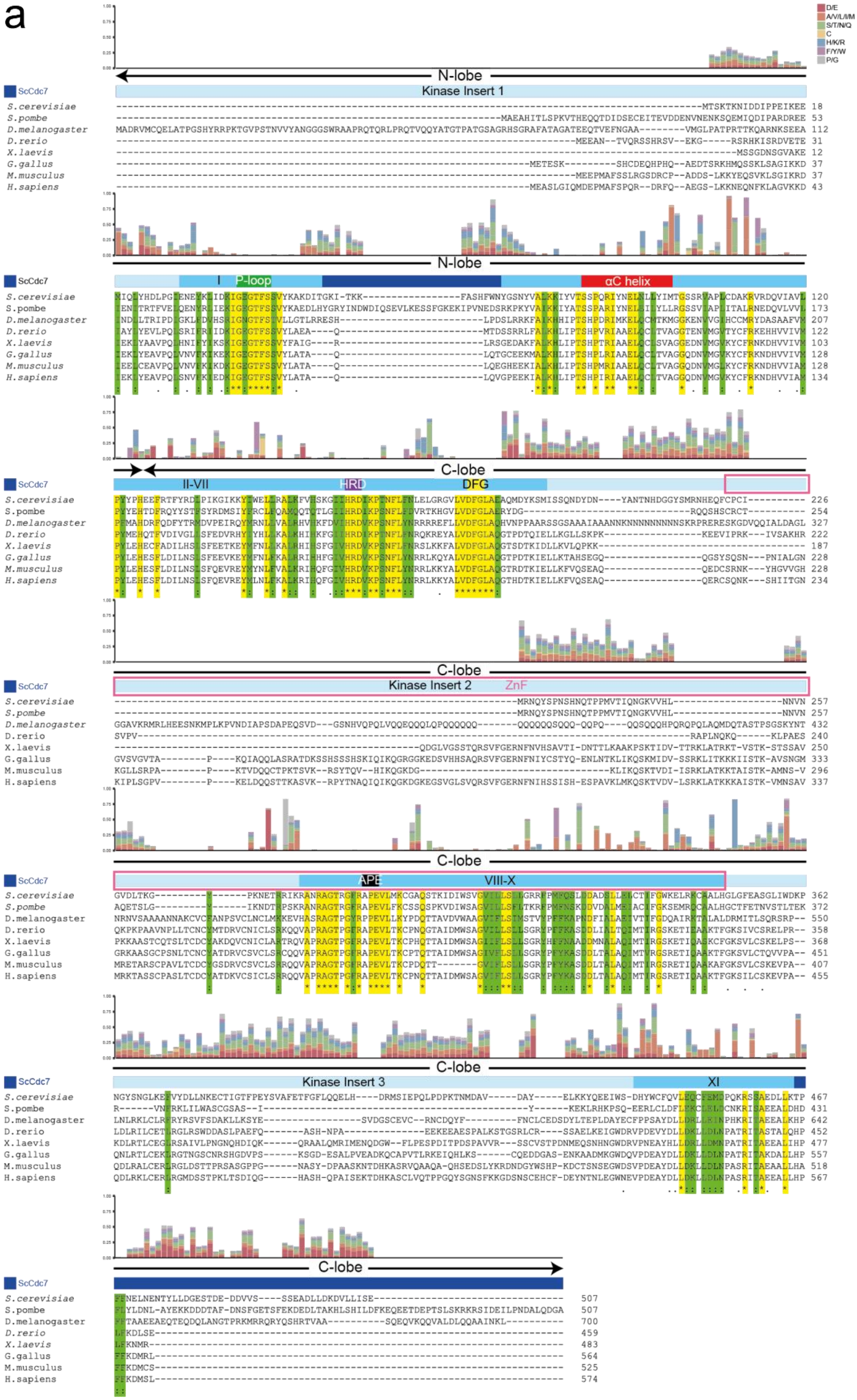
a
D/E
A/V/L/I/M
S/T/N/Q
C
H/K/R
F/Y/W
P/G
N-lobe
ScCdc7
Kinase Insert 1
S.cerevisiae
S.pombe
D.melanogaster
D.rerio
X.laevis
G.gallus
M.musculus
H.sapiens
I
P-loop
αC helix
C-lobe
II-VII
HRD
DFG
Kinase Insert 2
ZnF
APE
VIII-X
Kinase Insert 3
XI

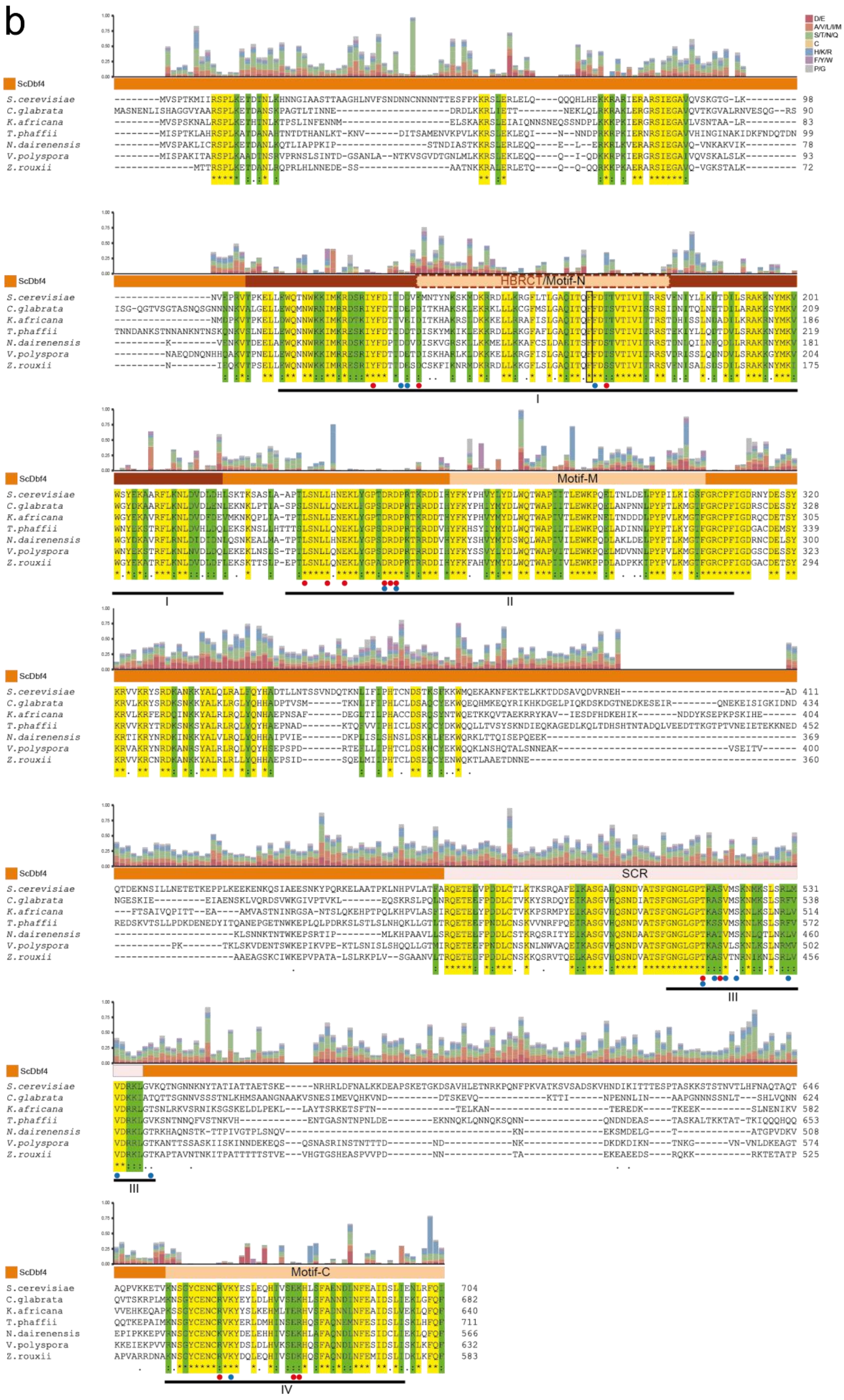
b
D/E
A/V/L/I/M
S/T/N/Q
C
H/K/R
F/Y/W
P/G
ScDbf4
S.cerevisiae
C.glabrata
K.africana
T.phaffii
N.dairenensis
V.polyspora
Z.rouxii
HBRCT/Motif-N
Motif-M
SCR
Motif-C
I
II
III
IV

Supplementary Figure 5. Multiple sequence alignment of Cdc7 and Dbf4 proteins showing transition probabilities for each residue.

Cdc7 and Dbf4 proteins were aligned using Clustal Omega. Highly conserved residues are coloured with a vertical yellow line and indicated by the symbol (*), highly similar residues are coloured with a vertical green line and indicated by the symbol (:), and residues with similar properties are indicated by the symbol (.). The predicted probability of transition of the initial standard protein residue to another amino acid type is shown. The probability of transition is represented as a stacked bar coloured by the type of amino acid. **a.** Aligned Cdc7 from *S. cerevisiae*, *Schizosaccharomyces pombe*, *Drosophila melanogaster, Danio rerio, Xenopus laevis, Gallus gallus, Mus musculus* and *Homo sapiens*. Cdc7 domain boundaries, kinase inserts and important motifs are highlighted above the alignment. **b.** Aligned Dbf4/Dbf4-type domain-containing proteins from the budding yeast species: *S. cerevisiae*, *Candida glabrata*, *Kazachstania africana*, *Tetrapisispora phaffii*, *Naumovozyma dairenensis*, *Zygosaccharomyces r ouxii* and *Vanderwaltozyma polyspora.* The Dbf4 protein residues which form contacts with MCM2-7 via backbone or side chain interactions are indicated by a blue or red circular dot, respectively. The black bar indicates the four (I-IV) distinct regions of Dbf4 which are at the interface with MCM2-7. Dbf4 F165 is highlighted with a black box. scDbf4 (orange horizontal bar), harbors the following domains: α-helix-BRCA1 C-terminal (HBRCT) domain, motif-N, motif-M and motif-C (named based on the positions along the polypeptide chain) and substrate coordinating region (SCR). The domain boundary of the SCR was determined from both structural data and the multiple sequence alignment shown.

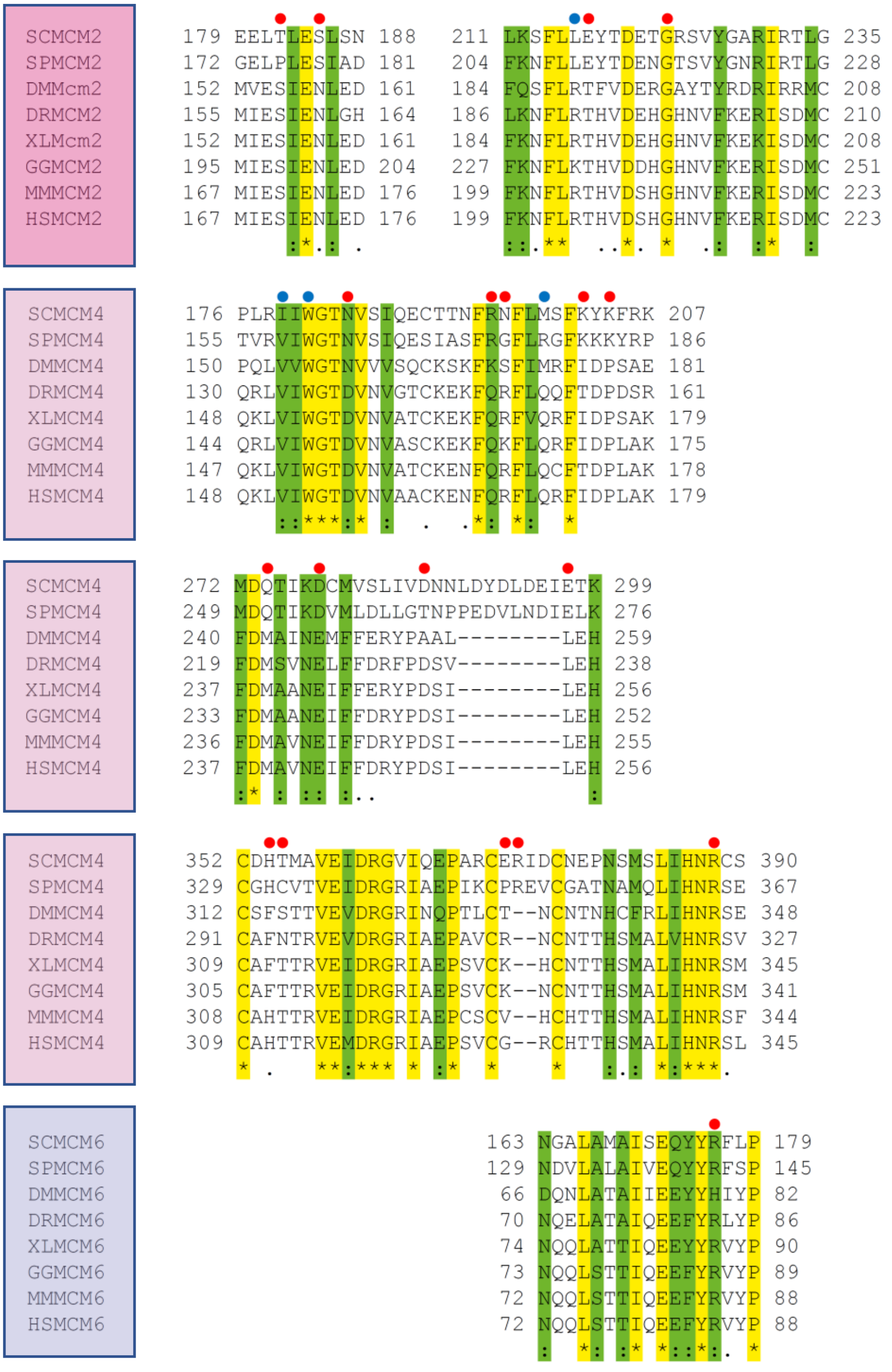

Supplementary Figure 6. MCM2-7 sequence alignment. Multiple sequence alignment of different regions of Mcm2, Mcm4 and Mcm6. The multiple sequence alignment was generated with various species using Clustal Omega. Highly conserved residues are coloured with a vertical yellow line and indicated by the symbol (*), highly similar residues are coloured with a vertical green line and indicated by the symbol (:), and residues with similar properties are indicated by the symbol (.). The residues of Mcm subunits that form interactions with Dbf4, in the MD-(ATPγS) state III atomic model, via backbone or side chain interactions are labelled by a blue dot or red dot, respectively. SC: *S. cerevisiae*, SP: *S. pombe*, DM: *D. melanogaster*, DR: *D. rerio*, XL: *X. laevis*, GG: *G. gallus*, MM: *M. musculus*, HS: *H. sapiens*.

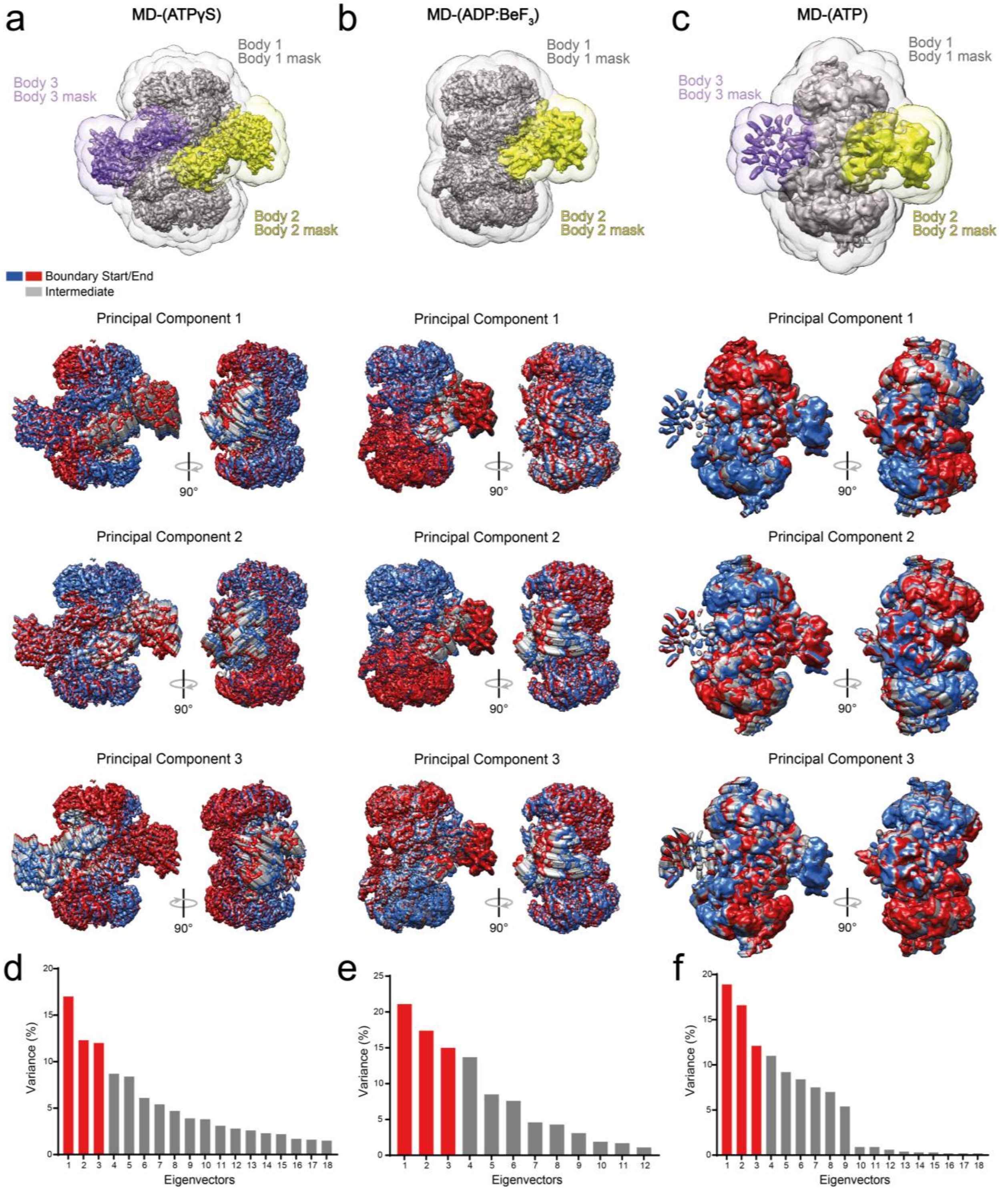

Supplementary Figure 7. Multi-body refinement and flexible analysis of MD complexes in the presence of different nucleotides. a-c. 3D auto-refined maps, of (**a**) MD-(ATPγS), (**b**) MD-(ADP:BeF_3) and (**c**) MD-(ATP) and associated segmented loose soft masks used for multi-body refinement and flexible analysis in RELION. Flexible analysis of the multi-body refinement of each different data set reveals the dynamic movements of DDK relative to the DH. The three main principal components are shown for each structure. **d-f.** The first 3 eigenvectors (red colored bars) explain 41.2%, 53.5% and 47.5% of the variance in the MD-(ATPγS), MD-(ADP:BeF_3) and MD-(ATP) data, respectively.

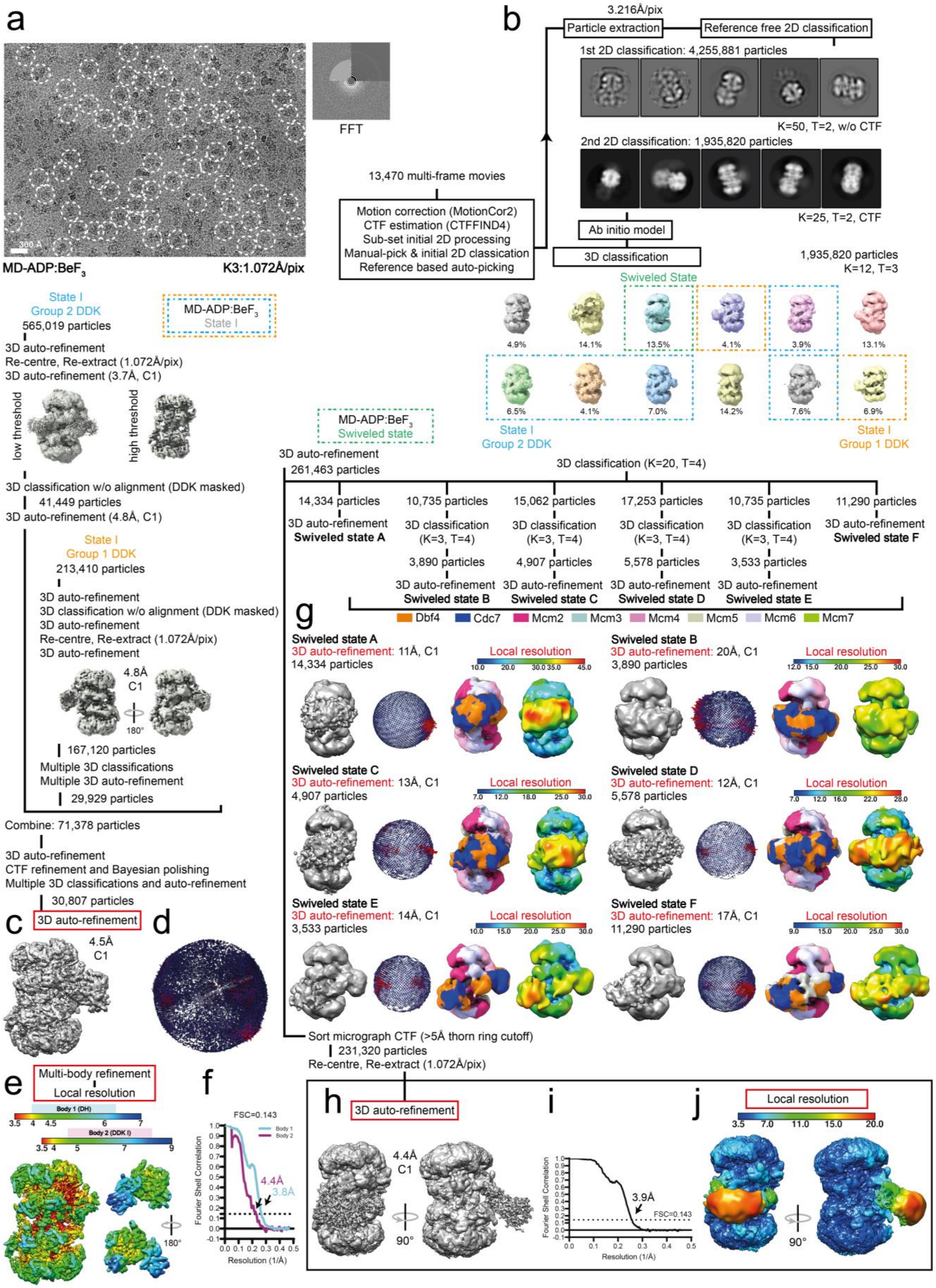
a
FFT
300 Å
MD-ADP:BeF3
K3:1.072Å/pix
b
3.216Å/pix
Particle extraction
Reference free 2D classification
1st 2D classification: 4,255,881 particles
K=50, T=2, w/o CTF
2nd 2D classification: 1,935,820 particles
K=25, T=2, CTF
13,470 multi-frame movies
Motion correction (MotionCor2)
CTF estimation (CTFFIND4)
Sub-set initial 2D processing
Manual-pick & initial 2D classication
Reference based auto-picking
Ab initio model
3D classification
1,935,820 particles
K=12, T=3
Swiveled State
4.9%
14.1%
13.5%
4.1%
3.9%
13.1%
6.5%
4.1%
7.0%
14.2%
7.6%
6.9%
State I
Group 2 DDK
State I
Group 1 DDK
State I
Group 2 DDK
565,019 particles
MD-ADP:BeF3
State I
3D auto-refinement
Re-centre, Re-extract (1.072Å/pix)
3D auto-refinement (3.7Å, C1)
low threshold
high threshold
3D classification w/o alignment (DDK masked)
41,449 particles
3D auto-refinement (4.8Å, C1)
State I
Group 1 DDK
213,410 particles
3D auto-refinement
3D classification w/o alignment (DDK masked)
3D auto-refinement
Re-centre, Re-extract (1.072Å/pix)
3D auto-refinement
4.8Å
C1
180°
167,120 particles
Multiple 3D classifications
Multiple 3D auto-refinement
29,929 particles
Combine: 71,378 particles
3D auto-refinement
CTF refinement and Bayesian polishing
Multiple 3D classifications and auto-refinement
30,807 particles
c
3D auto-refinement
4.5Å
C1
d
e
Multi-body refinement
Local resolution
Body 1 (DH)
3.5 4 4.5 6 7
Body 2 (DDK I)
3.5 4 5 7 9
180°
f
FSC=0.143
Body 1
Body 2
4.4Å
3.8Å
Fourier Shell Correlation
Resolution (1/Å)
MD-ADP:BeF3
Swiveled state
3D auto-refinement
261,463 particles
3D classification (K=20, T=4)
14,334 particles
3D auto-refinement
Swiveled state A
10,735 particles
3D classification
(K=3, T=4)
3,890 particles
3D auto-refinement
Swiveled state B
15,062 particles
3D classification
(K=3, T=4)
4,907 particles
3D auto-refinement
Swiveled state C
17,253 particles
3D classification
(K=3, T=4)
5,578 particles
3D auto-refinement
Swiveled state D
10,735 particles
3D classification
(K=3, T=4)
3,533 particles
3D auto-refinement
Swiveled state E
11,290 particles
3D auto-refinement
Swiveled state F
g
Dbf4
Cdc7
Mcm2
Mcm3
Mcm4
Mcm5
Mcm6
Mcm7
Swiveled state A
3D auto-refinement: 11Å, C1
14,334 particles
Local resolution
Swiveled state B
3D auto-refinement: 20Å, C1
3,890 particles
Local resolution
Swiveled state C
3D auto-refinement: 13Å, C1
4,907 particles
Local resolution
Swiveled state D
3D auto-refinement: 12Å, C1
5,578 particles
Local resolution
Swiveled state E
3D auto-refinement: 14Å, C1
3,533 particles
Local resolution
Swiveled state F
3D auto-refinement: 17Å, C1
11,290 particles
Local resolution
Sort micrograph CTF (>5Å thorn ring cutoff)
231,320 particles
Re-centre, Re-extract (1.072Å/pix)
h
3D auto-refinement
4.4Å
C1
90°
i
3.9Å
FSC=0.143
Fourier Shell Correlation
Resolution (1/Å)
j
Local resolution
3.5 7.0 11.0 15.0 20.0
90°

Supplementary Figure 8. Cryo-EM image processing work-flow and 3D reconstruction of the MD-(ADP:BeF_3) complex.

a. Representative cryo-EM micrograph and corresponding fast Fourier transform (FFT). The micrograph features monodisperse particles of MD-(ADP:BeF_3) which are indicated by dotted circles. Scale bar is also shown. **b.** Image data processing work-flow. **c.** 3D auto-refined map of MD-(ADP:BeF_3) state I at 4.5Å mean resolution. **d.** Euler angle particle distribution of map shown in (**c**). **e.** Multibody-body auto-refined MD-(ADP:BeF_3) state I maps of the DH and DDK. The MD-(ADP:BeF_3) state I map was split into two separate bodies for multi-body refinement and the resulting local resolution estimation of the two different bodies is shown. The front and back view of the body featuring mainly DDK regions is shown. **f.** Gold-standard Fourier Shell Correlation plot of masked multi-body MD-(ADP:BeF_3) maps. **g.** 3D auto-refined maps and local resolution estimation of six alternative swiveled structural states of the MD-(ADP:BeF_3) complex. The 3D auto-refined map, euler angle particle distribution, local resolution filtered map colored (using key indicated) according to manual fitting of complex subunits and local resolution filtered map are shown. **h.** 3D auto-refined map of MD-(ADP:BeF_3) swiveled state at 4.4Å mean resolution. **i.** Gold-standard Fourier Shell Correlation plot of post-processed MD-(ADP:BeF_3) swiveled state map with a loose soft mask and a B-factor of -30 applied. **j.** Local resolution estimation of the MD-(ADP:BeF_3) swiveled state map. The Euler angle particle distribution plots shown were generated using the RELION bild file output (blue bar represents the presence of a particle 2D view at a defined angle and red bar indicates a relatively high number of particles with that angle view). Resolution values shown are in angstroms.

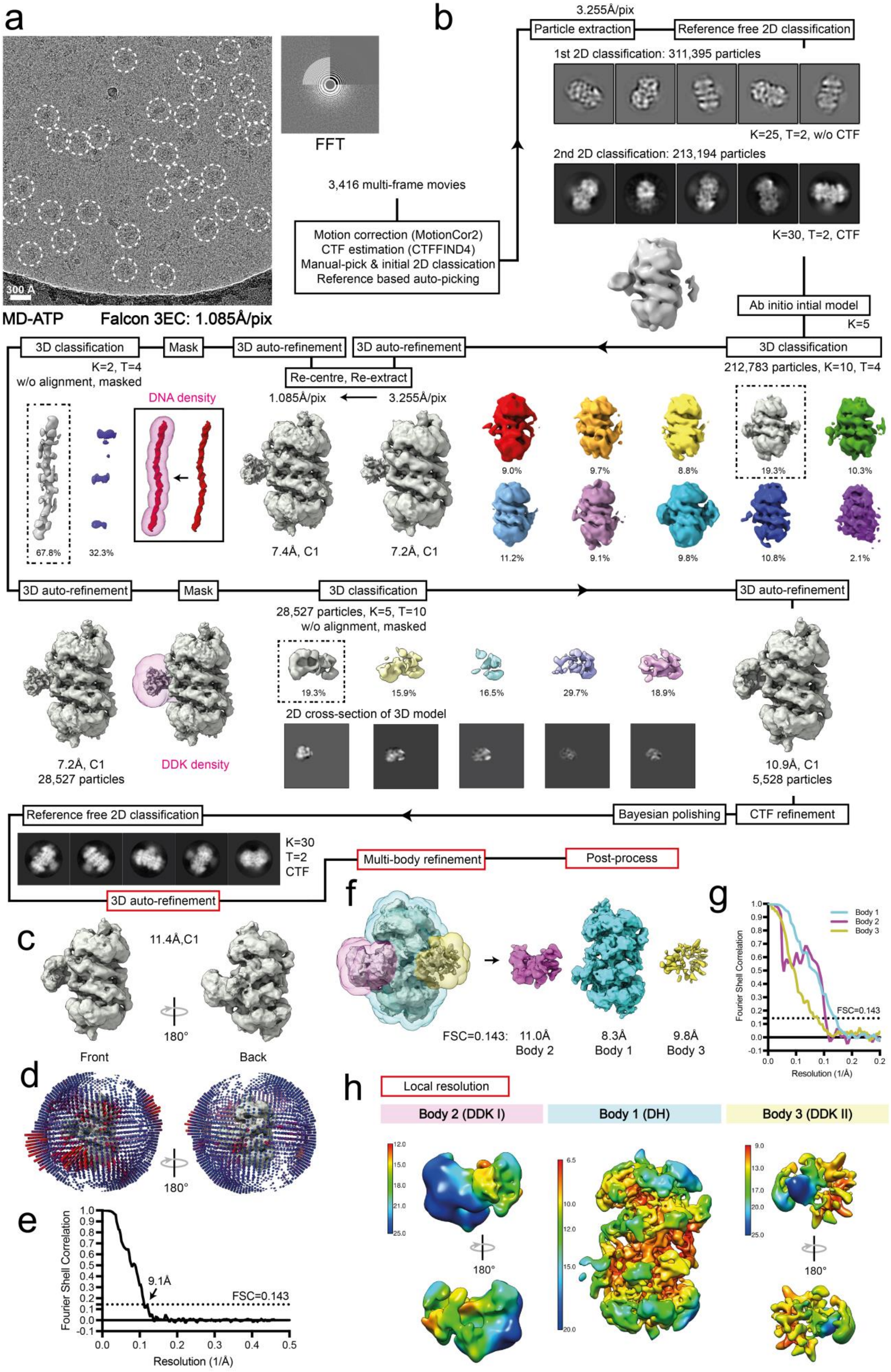
a
MD-ATP
Falcon 3EC: 1.085Å/pix
FFT
300 Å
b
3.255Å/pix
Particle extraction
Reference free 2D classification
1st 2D classification: 311,395 particles
K=25, T=2, w/o CTF
2nd 2D classification: 213,194 particles
K=30, T=2, CTF
3,416 multi-frame movies
Motion correction (MotionCor2)
CTF estimation (CTFFIND4)
Manual-pick & initial 2D classication
Reference based auto-picking
Ab initio intial model
K=5
3D classification
212,783 particles, K=10, T=4
3D auto-refinement
3D auto-refinement
Re-centre, Re-extract
1.085Å/pix
3.255Å/pix
Mask
3D classification
K=2, T=4
w/o alignment, masked
DNA density
67.8%
32.3%
7.4Å, C1
7.2Å, C1
9.0%
9.7%
8.8%
19.3%
10.3%
11.2%
9.1%
9.8%
10.8%
2.1%
3D auto-refinement
Mask
3D classification
28,527 particles, K=5, T=10
w/o alignment, masked
3D auto-refinement
19.3%
15.9%
16.5%
29.7%
18.9%
2D cross-section of 3D model
7.2Å, C1
28,527 particles
DDK density
10.9Å, C1
5,528 particles
Reference free 2D classification
Bayesian polishing
CTF refinement
K=30
T=2
CTF
Multi-body refinement
Post-process
3D auto-refinement
c
11.4Å,C1
180°
Front
Back
d
180°
e
Fourier Shell Correlation
9.1Å
FSC=0.143
Resolution (1/Å)
f
FSC=0.143:
11.0Å
Body 2
8.3Å
Body 1
9.8Å
Body 3
g
Body 1
Body 2
Body 3
Fourier Shell Correlation
FSC=0.143
Resolution (1/Å)
h
Local resolution
Body 2 (DDK I)
Body 1 (DH)
Body 3 (DDK II)
180°
180°

Supplementary Figure 9. Cryo-EM image processing work-flow and 3D reconstruction of the MD-(ATP) complex.

a. Representative cryo-EM micrograph and corresponding fast Fourier transform (FFT). The micrograph features monodisperse particles of MD-(ATP) which are indicated by dotted circles. Scale bar is also shown. **b.** Image data processing work-flow. **c.** 3D auto-refined map of MD-(ATP) at 11.4Å mean resolution. **d.** Euler angle particle distribution generated using the RELION bild file output (blue bar represents the presence of a particle 2D view at a defined angle and red bar indicates a relatively high number of particles with that angle view). **e.** Gold-standard Fourier Shell Correlation plot of post-processed MD-(ATP) map with a loose soft mask and a B-factor of -50 applied. **f.** The MD-(ATP) map split into three separate bodies for multi-body refinement. The bodies and the masks used are shown. **g.** Gold-standard Fourier Shell Correlation plot of masked multi-body MD-(ATP) maps. **h.** Local resolution estimation of the three different bodies of MD-(ATP). The front and back views of the bodies featuring mainly DDK regions are shown. Resolution values shown are in angstroms.

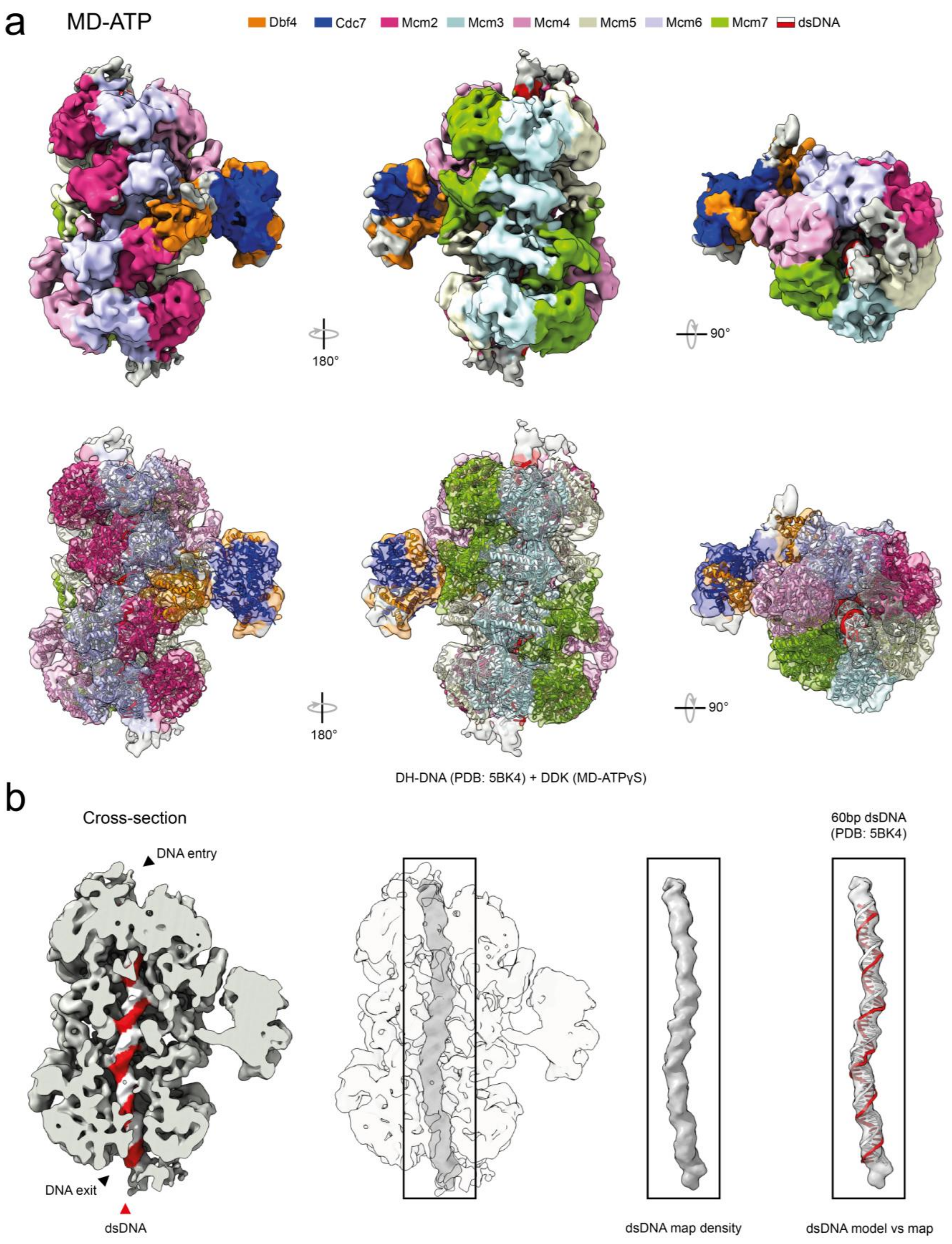

Supplementary Figure 10. Structure of DDK bound to the MCM2-7 double hexamer in the presence of ATP. a. Side and top views of the cryo-EM map of the MCM2-7-DDK (MD) complex in the presence of ATP. DH at 8.3Å mean resolution and DDK at 11.0Å mean resolution. The atomic models of the DH-DNA (PDB:5BK4) and DDK (MD-(ATPγS)) were docked into the cryo-EM multi-body maps of MD-(ATP). **b.** MD-(ATP) complexed with DNA. DNA is found at the center of the DH. Comparison of segmented DNA density from the MD-(ATP) map against the atomic DNA model from PDB:5BK4 reveals no differences in DNA shape or direction.

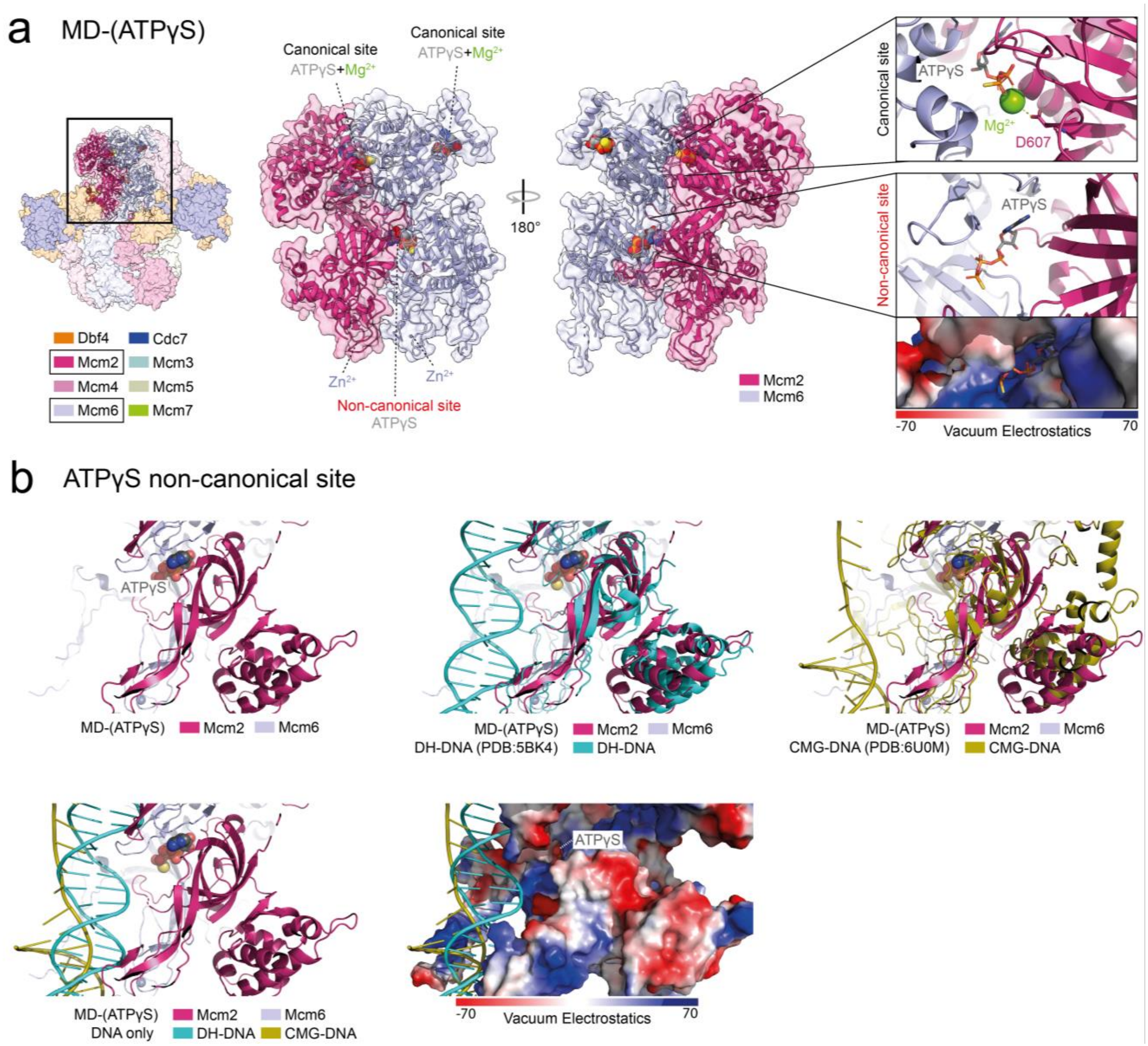

Supplementary Figure 11. Analysis of a non-canonical second ATPγS binding site between Mcm2 and Mcm6 within the MD-(ATPγS) complex.

a. Space-filling and cartoon view of the MD-(ATPγS) state III atomic model and focus on the Mcm2:Mcm6 interface. The Mcm2:Mcm6 interface featured an unexpected second ATPγS molecule at a non-canonical binding site. The ATPγS molecule was found to be less tightly sandwiched between Mcm2:Mcm6 than in canonical binding sites and the site featured a positively charged surface. **b.** Zoomed view of the ATPγS non-canonical binding site and comparison with DH-DNA and CMG-DNA structures. The dsDNA within the DH appears near the non-canonical binding site, suggesting this region may facilitate interaction with dsDNA. The ssDNA within the CMG, however rests further away from the site, suggesting the site is exclusive to dsDNA. The superimposed structures are colored according to the key and ATPγS is represented as a space-filling model.

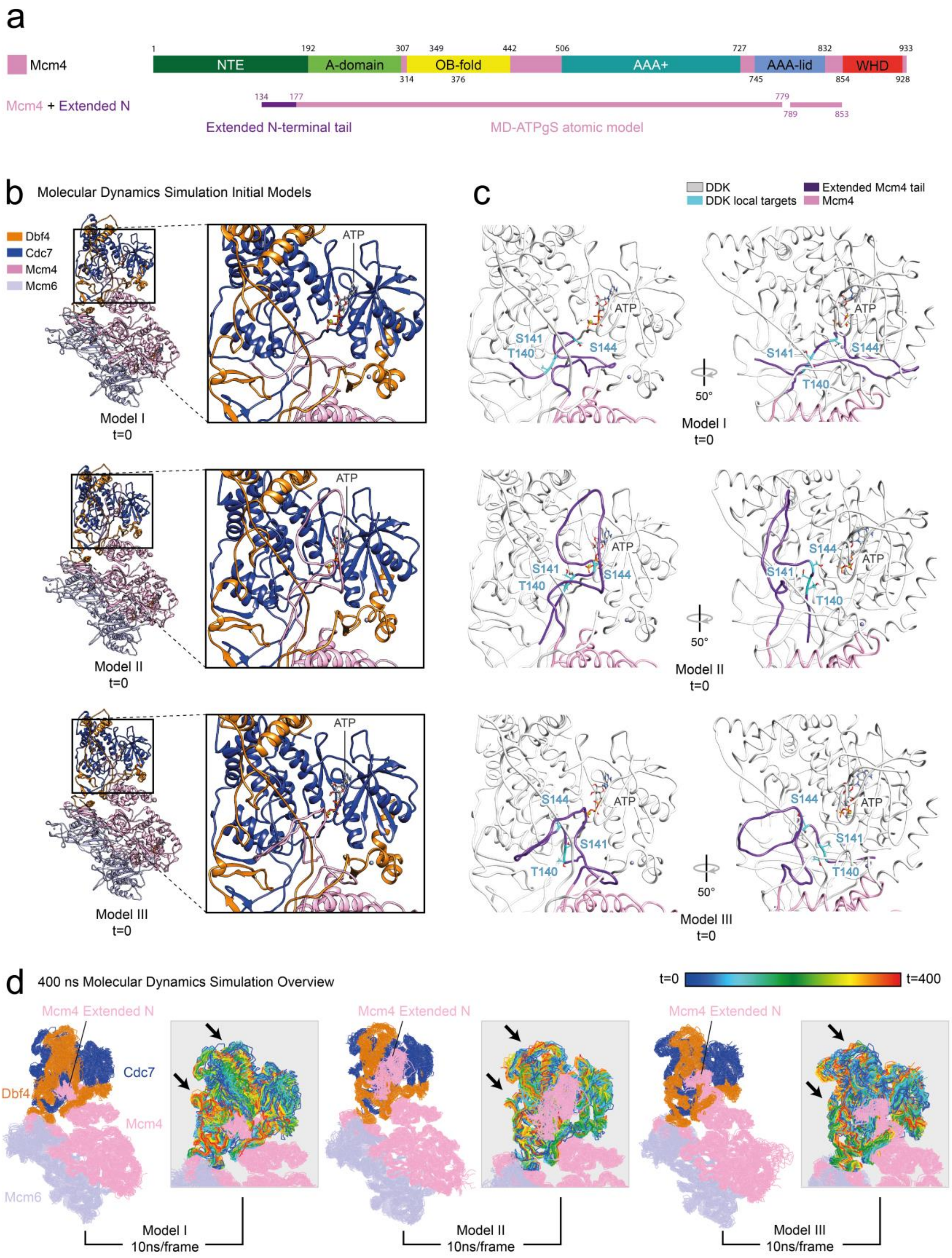
a
Mcm4
1
192
307
349
442
506
727
832
933
NTE
A-domain
OB-fold
AAA+
AAA-lid
WHD
314
376
745
854
928
Mcm4 + Extended N
134
177
779
789
853
Extended N-terminal tail
MD-ATPgS atomic model
b
Molecular Dynamics Simulation Initial Models
Dbf4
Cdc7
Mcm4
Mcm6
ATP
Model I
t=0
Model II
t=0
Model III
t=0
c
DDK
DDK local targets
Extended Mcm4 tail
Mcm4
S141
T140
S144
50°
d
400 ns Molecular Dynamics Simulation Overview
t=0
t=400
Mcm4 Extended N
Cdc7
Dbf4
Mcm4
Mcm6
Model I
10ns/frame
Model II
10ns/frame
Model III
10ns/frame

Supplementary Figure 12. Mcm4 N-terminal tail extension and molecular dynamics simulation overview.

a. Schematic diagram showing the extended N-terminal tail region of the Mcm4 (aa. 134-176) modelled using Rosetta (see Methods). Refer to Supplementary. Fig 4 for domain abbreviations. **b.** Zoom onto the initial models (model I-III), featuring different Mcm4 tail poses, used for molecular dynamics simulations. The Mcm4(N)-Mcm6-DDK models feature: an N-terminal extended Mcm4, Mcm6, Cdc7 and Dbf4 without the HBRCT domain. **c.** Focused view on the Mcm4 extended tail poses in each of the models. The serine and threonine DDK target residues along the tail and near the Cdc7 active site are indicated in cyan. **d.** Snapshots showing an overview of the GROMACS molecular dynamics simulation for each model. Frames of each model trajectory were sampled every 10 ns during the 400 ns simulation. The regions of each protein subunit within the superimposed frames are represented and the respective successive order of the frames for the DDK subunits are represented by rainbow colors (blue (0 ns) to red (400ns)). The black arrows indicate the regions of DDK that display the most global movement. The segment of Dbf4 that supports the Cdc7 C-lobe appears to display relatively more movement compared to the rest of DDK.

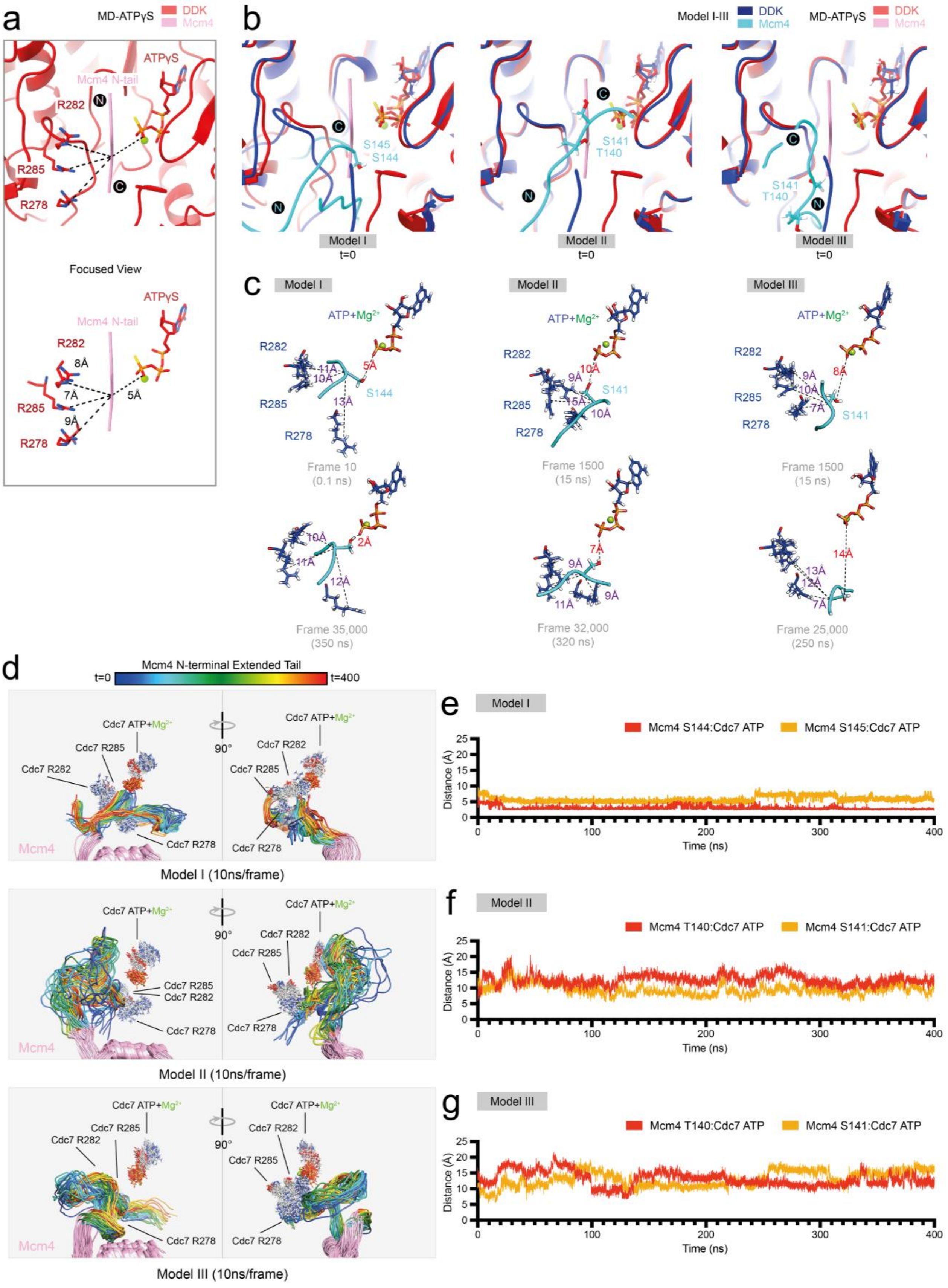

a
MD-ATPγS
DDK
Mcm4
ATPγS
Mcm4 N-tail
R282
R285
R278
N
C
Focused View
ATPγS
Mcm4 N-tail
R282
8Å
7Å
5Å
R285
9Å
R278
b
Model I-III
DDK
Mcm4
MD-ATPγS
DDK
Mcm4
S145
S144
Model I
t=0
S141
T140
Model II
t=0
S141
T140
Model III
t=0
c
Model I
ATP+Mg2+
R282
11Å
10Å
5Å
R285
S144
13Å
R278
Frame 10
(0.1 ns)
10Å
2Å
11Å
12Å
Frame 35,000
(350 ns)
Model II
ATP+Mg2+
R282
9Å
10Å
S141
R285
15Å
10Å
R278
Frame 1500
(15 ns)
7Å
9Å
11Å
9Å
Frame 32,000
(320 ns)
Model III
ATP+Mg2+
R282
9Å
8Å
R285
10Å
7Å
S141
R278
Frame 1500
(15 ns)
14Å
13Å
12Å
7Å
Frame 25,000
(250 ns)
d
Mcm4 N-terminal Extended Tail
t=0
t=400
Cdc7 ATP+Mg2+
Cdc7 R285
Cdc7 R282
90°
Cdc7 R278
Mcm4
Model I (10ns/frame)
Model II (10ns/frame)
Model III (10ns/frame)
e
Model I
Mcm4 S144:Cdc7 ATP
Mcm4 S145:Cdc7 ATP
Distance (Å)
Time (ns)
f
Model II
Mcm4 T140:Cdc7 ATP
Mcm4 S141:Cdc7 ATP
Distance (Å)
Time (ns)
g
Model III
Mcm4 T140:Cdc7 ATP
Mcm4 S141:Cdc7 ATP
Distance (Å)
Time (ns)

Supplementary Figure 13. Analysis and comparison of different Mcm4 extended N-tail trajectory models during a 400 ns molecular dynamics simulation

a. Zoomed view of the MD-(ATPγS) state III atomic model DDK active site and a focused view featuring the Mcm4 tail surrounded by Cdc7 R278, R282, R285 and ATPγS. The distance measurements between the backbone of the Mcm4 N-tail (CA atom) and the arginine residues (NE atom) or ATPγS (O3G atom) is indicated. **b.** Comparison of the different Mcm4 extended N-tail trajectory models (I-III) against the MD-(ATPγS) atomic model. **c.** Focused view of the DDK active site, showing the distance measurement between the nearest DDK Mcm4 target residue (OG atom) and Cdc7 ATPγS (O3G atom). **d.** Snapshots of model trajectory, sampled every 10 ns, during a 400 ns simulation. The successive order of the models is represented by rainbow colors (red to blue). The long flexible Mcm4 tail adopts multiple conformations throughout the simulation in all the models. **e-g.** Plot of distance measurement between the Mcm4 N-terminal tail (backbone CA atom) and Cdc7 ATPγS (O3G atom) throughout the entire simulation.

Supplementary Table 1. **Phosphorylation sites on MCM2-7 and Dbf4**.
Phosphorylation sites detected in all of three experiments are shown as +.

Protein	Position	PEP	Localization probability	Dbf4		
				WT	ΔN	SCR-G
DBF4	40	7.19E-185	1	+	+	+
DBF4	228	1.69E-16	0.99816	+	+	+
DBF4	235	2.04E-38	0.999698	+	+	+
DBF4	397	0.0094433	0.914762	-	+	+
DBF4	400	1.23E-09	1	-	+	+
DBF4	413	8.86E-11	1	-	+	+
DBF4	418	1.23E-07	0.999932	+	+	+
DBF4	424	2.82E-05	0.83599	-	+	-
DBF4	440	1.19E-08	1	+	+	+
DBF4	467	0.0014561	1	+	+	+
DBF4	473	5.09E-48	1	+	+	+
DBF4	482	0.0007366	0.999963	+	+	+
DBF4	496	1.31E-167	1	+	+	-
DBF4	501	2.16E-55	0.999937	+	+	-
DBF4	506	1.55E-08	0.999998	+	+	-
DBF4	507	1.55E-08	0.999994	+	+	-
DBF4	549	2.09E-07	0.999707	-	-	+
DBF4	551	4.04E-31	0.998231	-	+	+
DBF4	554	2.77E-39	0.911306	-	+	-
DBF4	581	0.0007897	0.999199	+	+	+
DBF4	585	0.0007875	0.989137	+	+	-
DBF4	620	0.0031949	0.988769	+	+	+
DBF4	621	0.005838	0.905227	-	+	+
MCM2	105	9.54E-06	0.983084	+	+	+
MCM2	107	9.54E-06	0.999998	+	+	+
MCM2	170	0	1	+	+	+
MCM2	725	0	1	+	-	+
MCM3	859	0.0005746	0.849302	+	+	-
MCM3	864	2.81E-19	0.998459	+	+	+
MCM4	52	2.82E-32	1	+	+	+
MCM4	56	3.04E-85	1	+	+	+
MCM4	68	2.74E-55	0.991699	-	+	+
MCM4	69	9.65E-16	0.835348	+	-	-
MCM4	75	2.86E-30	0.914681	+	-	+
MCM4	82	0.0001345	1	+	-	+
MCM4	87	6.14E-05	1	+	+	+
MCM4	112	0.0068063	0.932423	+	-	-
MCM4	118	0.0023802	0.99071	+	+	+
MCM4	119	0.0023802	0.872041	+	+	+

MCM4	140	0.0002194	0.999669	+	+	+
MCM4	141	0.0002194	0.999669	+	+	+
MCM4	144	4.56E-07	0.906055	+	+	+
MCM4	154	0.0239047	1	+	-	+
MCM4	171	2.43E-224	0.96727	+	+	-
MCM6	31	1.48E-53	0.74791	+	-	-
MCM6	47	9.36E-46	0.811268	+	+	+
MCM6	48	9.36E-46	0.811268	+	+	+
MCM6	49	9.36E-46	0.811268	+	+	+
MCM6	54	4.48E-22	0.806819	+	+	+
MCM6	56	4.48E-22	0.806819	+	+	+
MCM6	58	1.60E-20	0.760109	-	+	-
MCM6	75	4.41E-241	1	+	+	+
MCM6	78	2.04E-137	0.999823	+	+	+
MCM6	232	2.02E-12	0.867325	+	-	+
MCM7	175	1.08E-111	0.887763	-	-	+

Supplementary Table 2. Analysis of the interaction interface between Dbf4 and Cdc7 based on the MD-(ATPγS) state III atomic model. Polar contacts are coloured light blue and hydrophobic contacts are coloured yellow.

Cdc7		Dbf4	
Domain	Residue	Residue	Domain
I	Cdc7 E32/D108	Dbf4 R701	motif-C
II-VII	Backbone of Cdc7 V80	Dbf4 N663	motif-C
II-VII	Cdc7 T81	Dbf4 N510	SCR
II-VII	Backbone of Cdc7 T81	Backbone of Dfb4 E662	motif-C
II-VII	Cdc7 S83/R86	DBf4 E662	motif-C
II-VII	Cdc7 Y95	Dbf4 N699	motif-C
II-VII	Cdc7 R111	Dbf4 D687/D694	motif-C
II-VII	Cdc7 D114	Dbf4 H680	motif-C
II-VII	Cdc7 K138	Backbone of Dbf4 Y346/Q345	motif-M to SCR (connecting region)
KI-II	Backbone of Cdc7 L261	Dbf4 Q273	motif-M
KI-II	Backbone of Cdc7 Y265	Backbone of Dbf4 H259	motif-N to -M (connecting region)
KI-II	Backbone of Cdc7 K267	Backbone of Dbf4 D257	motif-N to -M (connecting region)
KI-II	Backbone of Cdc7 K267	Dbf4 K525	SCR
KI-II	Cdc7 R271	Backbone of Dbf4 Q273	motif-M
KI-II	Backbone of Cdc7 R272	Backbone of Dbf4 A276	motif-M
KI-II	Backbone of Cdc7 I273	Dbf4 S518	SCR
VIII-X	Cdc7 R316	Backbone of Dbf4 G302/I301/R306	motif-M
KI-III	Backbone of Cdc7 G349	Backbone of Dbf4 L271	motif-M
KI-III	Backbone of Cdc7 G351	Backbone of Dbf4 Y269	motif-M
KI-III	Backbone of Cdc7 E353	Backbone of Dbf4 Y267	motif-M
KI-III	Cdc7 E353	Dbf4 H265	motif-M
KI-III	Backbone of Cdc7 S355	Backbone of Dbf4 H265	motif-M
KI-III	Backbone of Cdc7 I358	Dbf4 Y296	motif-M
KI-III	Cdc7 E380	Backbone of Dbf4 G305	motif-M
KI-III	Cdc7 T385	Backbone of Dbf4 F304	motif-M
KI-III	Backbone of Cdc7 F386	Backbone of Dbf4 S374	motif-M to SCR (connecting region)
KI-III	Cdc7 Y389	Backbone of Dbf4 D330	motif-M to SCR (connecting region)
KI-III	Cdc7 E394	Dbf4 R341	motif-M to SCR (connecting region)
KI-III	Cdc7 Y431	Dbf4 Q345	motif-M to SCR (connecting region)
KI-III/VIII-X	Surface of Cdc7	Dbf4 F309/P308/L299/I301	motif-M
KI-III	Surface of Cdc7	Dbf4 V266/L268/I278/I279/L281/W283	motif-M
II-VII	Surface of Cdc7	Dbf4 A692/I693/L696	motif-C

Supplementary Table 3. Analysis of the interaction interface between Dbf4 and MCM2-7 based on the MD-(ATPγS) state III atomic model. Polar contacts are coloured light blue and hydrophobic contacts are coloured yellow.

Interaction interface	Dbf4	MCM2-7
I	Dbf4 Y127	Mcm2 E217
	Backbone of Dbf4 D132/D133	Mcm2 T182
	Dbf4 E135	Mcm4 S185
	Backbone of Dbf4 F166	Backbone of Mcm2 L216
	Dbf4 F165	Mcm2 surface pocket
	Dbf4 T168	Mcm2 G223
II	Dbf4 L234 and L238	Mcm6 surface
	Dbf4 E241	Mcm6 R176
	Dbf4 D248	Mcm4 R388
	Backbone of Dbf4 D248	Mcm4 T355
	Dbf4 R249	Mcm4 E372
	Dbf4 D250	Mcm4 R373
	Backbone of Dbf4 D250	Mcm4 H354
III	Dbf4 T515	Mcm4 D286
	Backbone of Dbf4 T515	Mcm4 R195
	Backbone of Dbf4 A517	Mcm4 D278
	Dbf4 S518	Mcm4 E297
	Backbone of Dbf4 V519	Mcm4 E297
	Backbone of Dbf4 S521/V519	Mcm4 Q274
	Backbone of Dbf4 L530	Backbone of Mcm4 W181
	Backbone of Dbf4 V532	Backbone of Mcm4 I179
	Backbone of Dbf4 V538	Mcm4 N184
IV	Dbf4 R665	Mcm4 D286
	Backbone of Dbf4 K667	Mcm4 N196
	Dbf4 E678	Mcm4 K202/K204
	Dbf4 K679	Backbone of Mcm4 M199

Supplementary Table 4. **Plasmid list**

Plasmid	Description	Source
pCS313	pESC-TRP MBP-Dbf4 Cdc7	Herrera et al., 2015
pCS1323	pESC-TRP MBP-Dbf4 Δ110 Cdc7	This study
pCS1324	pESC-TRP MBP-Dbf4 Δ220 Cdc7	This study
pCS1325	pESC-TRP MBP-Dbf4 ΔN motif Cdc7	This study
pCS1327	pESC-TRP MBP-Dbf4 SCR-G Cdc7	This study

Supplementary Table 5. Cryo-EM data collection parameters

Sample	MD-(ATPγS)	MD-(ADP:BeF_3)	MD-(ATP)
**Data collection parameters**			
**Microscope**	Titan Krios	Titan Krios	Titan Krios
**Magnification**	81,000x	81,000x	75,000x
**Voltage (kV)**	300	300	300
**Camera**	Gatan K3	Gatan K3	Falcon 3EC
**Imaging mode**	Super-resolution	Super-resolution	Counting
**Shots per hole**	3	3	2
**Total electron exposure (e–/Å^2)**	45.9	48.4	50.0
**Total number of frames**	54	40	19
**Defocus range (μm)**	-1.0 to -3.0	-1.7 to -3.7	-1.6 to -3.2
**Automated data collection software**	EPU	EPU	EPU
**Pixel size (Å)**	0.530	0.536	1.085

Supplementary Table 6. Summary of cryo-EM data processing of MD-(ATPγS) state I-III

Data processing	MD-(ATPγS) state I	MD-(ATPγS) state II	MD-(ATPγS) state III
**Number of multi-frame movies**	9,909		
**Symmetry imposed**	C1	C1	C1/C2
**Initial auto-picked particles (no.)**	2,572,593		
**Initial particle images (no.)**	258,684	138,647	1,235,868
**Final particle images (no.)**	12,800	22,459	73,093
**Map resolution (Å)**			
**FSC threshold (0.143) unmasked**	7.1	4.3	3.7
**FSC threshold (0.143) masked**	4.2	3.7	3.2
**Multi-body map**	-	Body 1: DH<br>Body 2: DDK I	Body 1: DH<br>Body 2: DDK I<br>Body 3: DDK II
**Local resolution range (Å)**	3.5 - 10.0	DH: 3.0 - 5.0<br>DDK I: 5.0 - 9.0	DH: 2.9 - 4.0<br>DDK I: 3.5 - 4.0<br>DDK II: 3.5 - 4.0
**Applied B-factor (Å^2)**	0	DH: -70<br>DDK I: -50	DH: -85<br>DDK I: -90<br>DDK II: -100

Supplementary Table 7. Summary of cryo-EM data processing of MD-(ADP:BeF3) state I and swivelled state

Data processing	MD-(ADP:BeF_3) state I	MD-(ADP:BeF_3) swiveled	MD-(ADP:BeF_3) swiveled state					
			A	B	C	D	E	F
**Number of multi-frame movies**	13,470							
**Symmetry imposed**	C1							
**Initial auto-picked particles (no.)**	4,255,881							
**Initial particle images (no.)**	778,429	261,463						
**Final particle images (no.)**	30,807	231,320	14,334	3,890	4,907	5,578	3,533	11,290
**Map resolution (Å)**								
**FSC threshold (0.143) unmasked**	4.1	4.4	11	20	13	12	14	17
**FSC threshold (0.143) masked**	3.6	3.9	9	18	9	9	12	13
**Multi-body map**	Body 1: DH<br>Body 2: DDK I	-	-	-	-	-	-	-
**Local resolution range (Å)**	DH: 3.5 - 6.0<br>DDK I: 4.0 - 7.0	3.5 - 20.0	10 - 45	12 - 25	7 - 30	7 - 28	10 - 25	9 - 25
**Applied B-factor (Å^2)**	DH: -30<br>DDK I: -50	-30	0	0	0	0	0	0

Supplementary Table 8. Summary of cryo-EM data processing of MD-(ATP)

**Data processing**	**MD-(ATP)**
**Number of multi-frame movies**	3,416
**Symmetry imposed**	C1
**Initial auto-picked particles (no.)**	311,395
**Initial particle images (no.)**	213,194
**Final particle images (no.)**	5,528
**Map resolution (Å)**	
**FSC threshold (0.143) unmasked**	11.4
**FSC threshold (0.143) masked**	9.1
**Multi-body map**	Body 1: DH<br>Body 2: DDK I<br>Body 3: DDK II
**Local resolution range (Å)**	DH: 6.5-15.0<br>DDK I: 12.0-25.0<br>DDK II: 9.0-25.0
**Applied B-factor (Å^2)**	Body 1: -100<br>Body 2: -50<br>Body 3: +100

Supplementary Table 9. Summary of structure validation statistics.

		MD-(ATPγS) state III	MD-(ADP:BeF3) state I
Initial model used (PDB code)		6YA7, 3QBZ, 6EYC, 6YAT	6YA8, 3QBZ, 6EYC, 6YAT
**Model**			
Composition (#)			
	Chains	18	15
	Atoms	74358 (Hydrogens: 0)	67420 (Hydrogens: 0)
	Residues	Protein: 9290 Nucleotide: 0	Protein: 8455 Nucleotide: 0
	Water	0	0
	Ligands	MG: 14	MG: 14
		AGS: 12	BEF: 7
		ZN: 14	ZN: 12
		ADP: 4	ADP: 13
Bonds (RMSD)			
	Length (Å) (# > 4σ)	0.010 (162)	0.008 (112)
	Angles (°) (# > 4σ)	1.315 (138)	1.051 (29)
MolProbity score		1.79	1.79
Clash score		11.96	11.49
Ramachandran plot (%)			
	Outliers	0.00	0.00
	Allowed	3.19	3.31
	Favored	96.81	96.69
Rama-Z (Ramachandran plot Z-score, RMSD)			
	whole (N = 9150)	-0.49 (0.09)	0.56 (0.09)
	helix (N = 3584)	0.41 (0.09)	1.56 (0.09)
	sheet (N = 1592)	-0.92 (0.12)	-0.38 (0.14)
	loop (N = 3974)	-0.61 (0.10)	-0.31 (0.10)
Rotamer outliers (%)		0.34	0.64
Cβ outliers (%)		0.34	0.05
Peptide plane (%)			
	Cis proline/general	0.0/0.0	0.0/0.0
	Twisted proline/general	0.0/0.0	0.0/0.0
CaBLAM outliers (%)		1.55	1.46
ADP (B-factors)			
	Iso/Aniso (#)	74358/0	67420/0
	Protein	1.24/112.22/30.74	9.99/149.96/66.43
	Ligand	12.06/136.68/68.98	13.70/224.89/69.88
Occupancy			
	Mean	1.00	1.00
	occ = 1 (%)	100.00	100.00
	0 < occ < 1 (%)	0.00	0.00
	occ > 1 (%)	0.00	0.00
**Model vs. Data**			
	CC (mask)	0.80	0.77
	CC (box)	0.69	0.74
	CC (peaks)	0.66	0.67
	CC (volume)	0.78	0.77
	Mean CC for ligands	0.73	0.73
