# Supplementary material for: The structural basis of Cdc7-Dbf4 kinase dependent targeting and phosphorylation of the MCM2-7 double hexamer
Source: Nat Commun. 2022 May 25;13:2915. doi: 10.1038/s41467-022-30576-1 (PMC9133112; doi:10.1038/s41467-022-30576-1)
Supplement: Supplementary file 2 — Description of Additional Supplementary Files [file 41467_2022_30576_MOESM2_ESM.pdf]

Supplementary Movie 1. DDK recruitment to the MCM2-7 DH.

The cryo-EM structures of the MCM2-7-DDK (MD) complex in the presence of either ATPγS or ADP:BeF_3 show the recruitment mechanism of DDK to the MCM2-7 DH and the different DDK binding modes. The MD-(ATPγS) state I-III maps reveal initial DDK anchoring to Mcm2 via the Dbf4 HBRCT domain, followed by DDK docking in an Mcm4 DDK targeted conformation. The MD-(ADP:BeF_3) state I and swiveled state map reveal the different DDK binding modes, explaining DDK targeted phosphorylation of Mcm4 and Mcm2/6.

Supplementary Movie 2. Flexible analysis of MD-(ATPγS) state III multi-body refinement.

The MD-(ATPγS) state III map was split into three bodies (separating the MCM2-7 DH and each of the two DDK subunits) and subjected to multi-body refinement. The distinct movements of DDK relative to the DH are shown.

Supplementary Movie 3. Molecular dynamics simulation of Mcm4(N)-Mcm6-DDK model I.

The Mcm4(N)-Mcm6-DDK model I structure, which features an extended Mcm4 N-terminal tail (aa. 134-176), was simulated for a total of 400 ns. The GROMACS molecular dynamics simulation is shown with different views and different frame steps (either 100 or 500 frame steps during the 40,000-frame simulation).

Supplementary Movie 4. Molecular dynamics simulation of Mcm4(N)-Mcm6-DDK model II.

The Mcm4(N)-Mcm6-DDK model II structure, which features an extended Mcm4 N-terminal tail (aa. 134-176), was simulated for a total of 400 ns. The GROMACS molecular dynamics simulation is shown with different views and different frame steps (either 100 or 500 frame steps during the 40,000-frame simulation).

Supplementary Movie 5. Molecular dynamics simulation of Mcm4(N)-Mcm6-DDK model III.

The Mcm4(N)-Mcm6-DDK model III structure, which features an extended Mcm4 N-terminal tail (aa. 134-176), was simulated for a total of 400 ns. The GROMACS molecular dynamics simulation is shown with different views and different frame steps (either 100 or 500 frame steps during the 40,000-frame simulation).
