# Supplementary material for: The structural basis of Cdc7-Dbf4 kinase dependent targeting and phosphorylation of the MCM2-7 double hexamer
Source: Nat Commun. 2022 May 25;13:2915. doi: 10.1038/s41467-022-30576-1 (PMC9133112; doi:10.1038/s41467-022-30576-1)
Supplement: Supplementary file 8 — Reporting Summary [file 41467_2022_30576_MOESM8_ESM.pdf]

nature portfolio

Corresponding author(s): Christian Speck

Last updated by author(s): Apr 29, 2022

Reporting Summary

Nature Portfolio wishes to improve the reproducibility of the work that we publish. This form provides structure for consistency and transparency in reporting. For further information on Nature Portfolio policies, see our Editorial Policies and the Editorial Policy Checklist.

Statistics

For all statistical analyses, confirm that the following items are present in the figure legend, table legend, main text, or Methods section.

n/a	Confirmed	
☐	☒	The exact sample size (n) for each experimental group/condition, given as a discrete number and unit of measurement
☒	☐	A statement on whether measurements were taken from distinct samples or whether the same sample was measured repeatedly
☐	☒	The statistical test(s) used AND whether they are one- or two-sided<br>*Only common tests should be described solely by name; describe more complex techniques in the Methods section.*
☒	☐	A description of all covariates tested
☒	☐	A description of any assumptions or corrections, such as tests of normality and adjustment for multiple comparisons
☒	☐	A full description of the statistical parameters including central tendency (e.g. means) or other basic estimates (e.g. regression coefficient) AND variation (e.g. standard deviation) or associated estimates of uncertainty (e.g. confidence intervals)
☒	☐	For null hypothesis testing, the test statistic (e.g. F, t, r) with confidence intervals, effect sizes, degrees of freedom and P value noted<br>*Give P values as exact values whenever suitable.*
☒	☐	For Bayesian analysis, information on the choice of priors and Markov chain Monte Carlo settings
☒	☐	For hierarchical and complex designs, identification of the appropriate level for tests and full reporting of outcomes
☒	☐	Estimates of effect sizes (e.g. Cohen's d, Pearson's r), indicating how they were calculated

Our web collection on statistics for biologists contains articles on many of the points above.

Software and code

Policy information about availability of computer code

Data collection	LC-MS/MS was performed for proteomics data collection. EPU (version 2) software was used for TEM data collection.
Data analysis	MotionCor2 (version 1.4.0), CTFFIND4 (version 4.1.14) and RELION (version 3.1.3) for Cryo-EM image processing. Coot (version 0.9.6), SWISS model (web-based integrated service), UCSF Chimera (version 1.15), UCSF ChimeraX (version 1.3), PSIPRED (web-based integrated service), the auto-sharpen program in Phenix for atomic model building and the combine focused maps program in Phenix. Molprobity (version 4.1) for structure validation. MaxQuant (version 2.1.0.0) and Perseus (version 1.6.15.0) for Mass spectrometry data processing. Clustal Omega (web-based integrated service) for sequence alignment. Rosetta (version 3.2) was used for ab initio protein structure prediction and GROMACS (version 2021.2) for molecular dynamics simulation and trajectory analysis.

For manuscripts utilizing custom algorithms or software that are central to the research but not yet described in published literature, software must be made available to editors and reviewers. We strongly encourage code deposition in a community repository (e.g. GitHub). See the Nature Portfolio guidelines for submitting code & software for further information.

Data

Policy information about availability of data

All manuscripts must include a data availability statement. This statement should provide the following information, where applicable:

- Accession codes, unique identifiers, or web links for publicly available datasets
- A description of any restrictions on data availability
- For clinical datasets or third party data, please ensure that the statement adheres to our policy

The EM maps and associated atomic models of MD-(ATPγS) state III and MD-(ADP:BeF3) state I reported in this study have been deposited to the Electron Microscopy Data Bank (EMDB) and the RCSB Protein Data Bank (PDB) under accession numbers EMD-13619/PDB: 7PT6 and EMD-13620/PDB: 7PT7, respectively.

The EM maps of MD-(ATPγS) state I-II and additional maps of state I-III have been deposited under the accession numbers: EMD-13644, EMD-13640, EMD-13631, EMD-13635, EMD-13629, EMD-13624, EMD-13621, EMD-13622, EMD-13623. The EM maps of MD-(ADP:BeF3) swiveled states and additional maps of state I have been deposited under the accession numbers: EMD-13647, EMD-13645, EMD-13646, EMD-13648, EMD-13649, EMD-13650, EMD-13651, EMD-13652, EMD-13653, EMD-13655. The EM maps of MD-(ATP) have been deposited under the accession numbers: EMD-13656, EMD-13657, EMD-13658, EMD-13659. Proteomics data have been deposited to PRIDE under the accession number: PXD031315 (https://www.ebi.ac.uk/pride/). Other data that support the study are available from the corresponding author upon reasonable request.

Field-specific reporting

Please select the one below that is the best fit for your research. If you are not sure, read the appropriate sections before making your selection.

- [x] Life sciences
- [ ] Behavioural & social sciences
- [ ] Ecological, evolutionary & environmental sciences

For a reference copy of the document with all sections, see nature.com/documents/nr-reporting-summary-flat.pdf

Life sciences study design

All studies must disclose on these points even when the disclosure is negative.

Sample size	No statistical methods were used to predetermine sample size, as much data as possible was collected during allocated instrument session time. We collected 2,572,593 particles from 9,772 micrographs for MD-ATPγS, 4,255,881 particles from 13,470 micrographs for MD-ADP:BeF3 and 311,395 particles from 3,416 micrographs for MD-ATP.
Data exclusions	Data was excluded at different stages of image processing. Particles that did not meet the image resolution quality criteria or could not be averaged were excluded from the final structures, based on 2D averages and 3D map classification results. The method of data exclusion is common practice in the cryo-EM field to allow for the generation of interpretable 3D models.
Replication	All attempts to replicate results were successful. Biochemical experiments were repeated at least two or three times as biological replicates.
Randomization	Experiments were not randomized due to the nature of the data not involving patients or animals. The experiments were not grouped and needed to be preformed in a particular order.
Blinding	Blinding is not applicable to this research, which does not entail randomization and does not involve patients or animals. The experiments required essential visual inspection of the data quality during data screening and data collection and so no blinding could be performed.

Reporting for specific materials, systems and methods

We require information from authors about some types of materials, experimental systems and methods used in many studies. Here, indicate whether each material, system or method listed is relevant to your study. If you are not sure if a list item applies to your research, read the appropriate section before selecting a response.

Materials & experimental systems

n/a	Involved in the study	
x		Antibodies
x		Eukaryotic cell lines
x		Palaeontology and archaeology
x		Animals and other organisms
x		Human research participants
x		Clinical data
x		Dual use research of concern

Methods

n/a	Involved in the study	
x		ChIP-seq
x		Flow cytometry
x		MRI-based neuroimaging
